# Low-Dose Yellow Fever Vaccine in Adults in Africa

**DOI:** 10.1056/NEJMoa2407293

**Published:** 2025-02-20

**Authors:** Derick Kimathi, Aitana Juan-Giner, Ndeye S. Bob, Benedict Orindi, Maria L. Namulwana, Antoine Diatta, Stanley Cheruiyot, Gamou Fall, Moussa Dia, Mainga M. Hamaluba, Dan Nyehangane, Henry K. Karanja, John N. Gitonga, Daisy Mugo, Donwilliams O. Omuoyo, Mwatasa Hussein, Elizaphan Oloo, Naomi Kamau, Jackline Wafula, Josephine Bendera, Namanya Silvester, James Mwavita, Musiimenta Joshua, Jane Mwendwa, Collins Agababyona, Caroline Ngetsa, Nalusaji Aisha, Felix Moki, Titus Buluku, Marianne Munene, Juliet Mwanga-Amumpaire, Julius Lutwama, John Kayiwa, Eunice Kamaara, Alan D. Barrett, Pontiano Kaleebu, Philip Bejon, Amadou A. Sall, Rebecca F. Grais, George M. Warimwe, for the NIFTY Investigators

**Affiliations:** 1 Kenya Medical Research Institute–Wellcome Trust Research Programme, Kilifi, Kenya; 2 Epicentre, Paris; 3 Institut Pasteur de Dakar, Dakar, Senegal; 4 Epicentre Mbarara Research Centre, Mbarara, Uganda; 5 Uganda Virus Research Institute (UVRI), Entebbe; 6 Moi University, Eldoret, Kenya; 7 Sealy Institute for Vaccine Sciences, University of Texas Medical Branch, Galveston; 8 Department of Pathology, University of Texas Medical Branch, Galveston; 9 Medical Research Council− UVRI and the London School of Hygiene and Tropical Medicine, Uganda Research Unit, Entebbe, Uganda; 10 Nuffield Department of Medicine, University of Oxford, Oxford, United Kingdom

## Abstract

**Background:**

Yellow fever vaccine is highly effective with a single dose, but vaccine supply is limited. The minimum dose requirements for seroconversion remain unknown.

**Methods:**

In this double-blind, randomized, noninferiority trial in Uganda and Kenya, we assigned adults with no history of yellow fever vaccination or infection to receive vaccination with the Institut Pasteur de Dakar 17D-204 yellow fever vaccine at a standard dose (13,803 IU) or at a fractional dose of 1000 IU, 500 IU, or 250 IU. The primary outcome was seroconversion at 28 days after vaccination with each fractional dose as compared with the standard dose, evaluated in a noninferiority analysis. Seroconversion was defined as an antibody titer at day 28 that was at least four times as high as the antibody titer before vaccination, as measured by a plaque reduction neutralization test. We conducted noninferiority analyses in the per-protocol and intention-to-treat populations. Noninferiority was shown if the lower boundary of the 95% confidence interval for the difference in the incidence of seroconversion between the fractional dose and the standard dose was higher than −10 percentage points.

**Results:**

A total of 480 participants underwent randomization (120 participants in each group). The incidence of seroconversion was 98% (95% confidence interval [CI], 94 to 100) with the standard dose. The difference in the incidence of seroconversion between the 1000-IU dose and the standard dose was 0.01 percentage points (95% CI, −5.0 to 5.1) in the intention-to-treat population and −1.9 percentage points (95% CI, −7.0 to 3.2) in the per-protocol population; the corresponding differences between the 500-IU dose and the standard dose were 0.01 percentage points (95% CI, −5.0 to 5.1) and −1.8 percentage points (95% CI, −6.7 to 3.2), and those between the 250-IU dose and the standard dose were −4.4 percentage points (95% CI, −9.4 to 0.7) and −6.7 percentage points (95% CI, −11.7 to 1.6). A total of 111 vaccine-related adverse events were reported: 103 were mild in severity, 7 were moderate, and 1 was severe. The incidence of adverse events was similar in the four groups.

**Conclusions:**

A yellow fever vaccination dose as low as 500 IU was noninferior to the standard dose of 13,803 IU for producing seroconversion within 28 days. (Funded by the European and Developing Countries Clinical Trials Partnership and the Wellcome Trust; NIFTY ClinicalTrials.gov number, NCT04059471.)

Yellow fever is a mosquito-borne viral zoonotic illness that is endemic in Africa and South America.^1^ Yellow fever out-breaks are most commonly reported in Africa, where more than 100,000 severe cases of yellow fever, with a case fatality of 20 to 60%, are estimated to occur annually.^2,3^

The 17D live-attenuated yellow fever vaccine is safe and effective; a single dose provides lifelong immunity.^4^ However, the process of manufacturing the yellow fever vaccine is laborious and difficult to scale up rapidly, factors that lead to persistent challenges with regard to vaccine availability.^5^

The World Health Organization (WHO) recommends the use of fractional doses of the vaccine as a dose-sparing strategy during outbreaks when there are vaccine shortages.^6^ This policy is supported by evidence from trials evaluating the immunogenicity and safety of one fifth of a standard dose of vaccine produced by each of the four WHO-prequalified vaccine manufacturers in healthy adults and children and in persons infected by the human immunodeficiency virus (HIV).^7–10^ However, because the potency of doses can vary substantially according to manufacturer and batch,^7^ the generalizability of these data may be limited.

A dose-finding trial that was carried out in Brazil with the use of the 17DD vaccine substrain showed that a dose of 587 IU resulted in seroconversion in 96.9% of the participants (95% confidence interval [CI], 92.4 to 99.2), and a dose of 158 IU resulted in seroconversion in 88.5% of the participants (95% CI, 81.5 to 93.6).^11^ This trial involved young men with a mean age of 19.4 years, and hence further data are needed.

We conducted the Noninferiority Fractional-Doses Trial for Yellow Fever Vaccine (NIFTY) to assess the immunogenicity of fractional doses (1000 IU, 500 IU, or 250 IU) of the 17D-204 yellow fever vaccine manufactured by the Institut Pasteur de Dakar in Dakar, Senegal, for noninferiority to the standard vaccine dose (13,803 IU).

## Methods

### Trial Design and Participants

This double-blind, randomized, noninferiority trial was conducted at the Kenya Medical Research Institute−Wellcome Trust Research Programme Clinical Trials Facility in Kilifi County, Kenya, and the Epicenter Mbarara Research Centre in Mbarara City, Uganda. We conducted community meetings to present the trial design, the rationale for conducting the trial of fractional doses, the expectations for participation, and the possible effects that could result from the data generated by the trial. Volunteers were eligible for inclusion if they were between 18 and 59 years of age; were either HIV-negative on a standard serologic screening test or HIV-positive, receiving treatment, and in clinically stable condition with a CD4 count of more than 200 cells per microliter^12^; were not pregnant or lactating; had no history of wild-type yellow fever virus infection or vaccination with yellow fever vaccine; and were able to adhere to the trial procedures.^13^

The participants were randomly assigned to receive vaccination with yellow fever vaccine at a full standard dose (13,803 IU) or at a fractionated dose of 1000 IU, 500 IU, or 250 IU. Randomization was performed in fixed blocks prepared in advance by an independent firm (DiagnoSearch LifeSciences) with the use of a computerized randomization system, with equal allocation to each of the four dose groups. The various dose levels were prepared according to the manufacturer’s instructions (see the Supplementary Methods section and Table S1 in the Supplementary Appendix, available with the full text of this article at NEJM.org). Details regarding the conduct of the trial are provided in the protocol, available at NEJM.org.

### Trial Oversight

Oversight of the trial was provided by the University of Oxford. The trial protocol was reviewed and approved by the Oxford Tropical Research Ethics Committee, the Kenya Medical Research Institute Scientific and Ethics Review Unit, Mbarara University of Science and Technology Research Ethics Committee, and the Uganda National Council of Sciences and Technology. Regulatory approval was obtained from the Kenya Pharmacy and Poisons Board and the Uganda National Drug Authority. The authors vouch for the accuracy and completeness of the data and for the fidelity of the trial to the protocol. The funders had no role in the trial design, data collection and analysis, preparation of the manuscript, or decision to submit the manuscript for publication.

### Trial Procedures and Follow-up

All the vaccine doses were administered subcutaneously in a volume of 0.5 ml with the use of autodisable syringes with a 25-gauge, 0.75-in. needle and a 45-degree injection angle. For assessment of immunogenicity, blood samples were collected at baseline, at 10 days and 28 days after vaccination, and then at 1 year and 2 years after vaccination. For viremia monitoring, blood samples were collected at baseline and day 10, and participants were randomly assigned to have one additional blood-sampling visit at day 2, 3, 4, 5, 6, or 7 (i.e., 80 participants on each day) for assessment of post-vaccination viremia. Blood was processed to obtain serum that was stored at −80°C until assayed.

### Laboratory Assessments

The plaque reduction neutralization test (PRNT) assays for this trial were conducted according to well-described standardized methods at the WHO-accredited regional yellow fever reference laboratory at the Institut Pasteur de Dakar.^14,15^ Seroconversion was defined as antibody titers on a 50% PRNT (PRNT_50_) assay that were at least four times as high as the titers before vaccination. Postvaccination viremia was assessed with the use of a standard reverse-transcriptase−polymerase-chain-reaction (RT-PCR) assay at the WHO-accredited regional yellow fever reference laboratory at the Uganda Virus Research Institute, Entebbe, Uganda.

### Sample Size

The primary outcome was seroconversion at 28 days after vaccination with each fractional dose as compared with the standard dose, evaluated in a noninferiority analysis. Noninferiority was shown if the lower boundary of the 95% confidence interval of the difference in the incidence of seroconversion was greater than −10 percentage points.

Assuming a 95% incidence of seroconversion, we estimated that 120 participants per dose group would provide the trial with 90% power to determine noninferiority at a margin of −10 percentage points and at a one-sided alpha level of 0.025. The noninferiority margin was based on the public health consequences of waning population immunity (see the Supplementary Methods).^16^

### Statistical Analysis

Analyses were performed in the intention-to-treat population (which included all participants who underwent randomization) and in the per-protocol population (which included participants who underwent randomization and were seronegative for yellow fever at baseline and had blood samples obtained at baseline and 28±3 days after vaccination). Secondary outcome analyses included assessing (with the use of a PRNT_90_ assay) the noninferiority of the proportion of participants who had seroconversion, determining geometric mean titers and geometric mean factor increases in titers (i.e., the geometric mean of the ratios of postvaccination titers to prevaccination titers) on PRNT_50_ and PRNT_90_ assays, and assessing the incidence of seroconversion on a PRNT_90_ assay in the intention-to-treat and per-protocol populations. When data were available for analysis, exclusions from the per-protocol analysis were made only on the basis of baseline characteristics and not on the basis of postran-domization events.

## Results

### Participants

**Figure 1:**
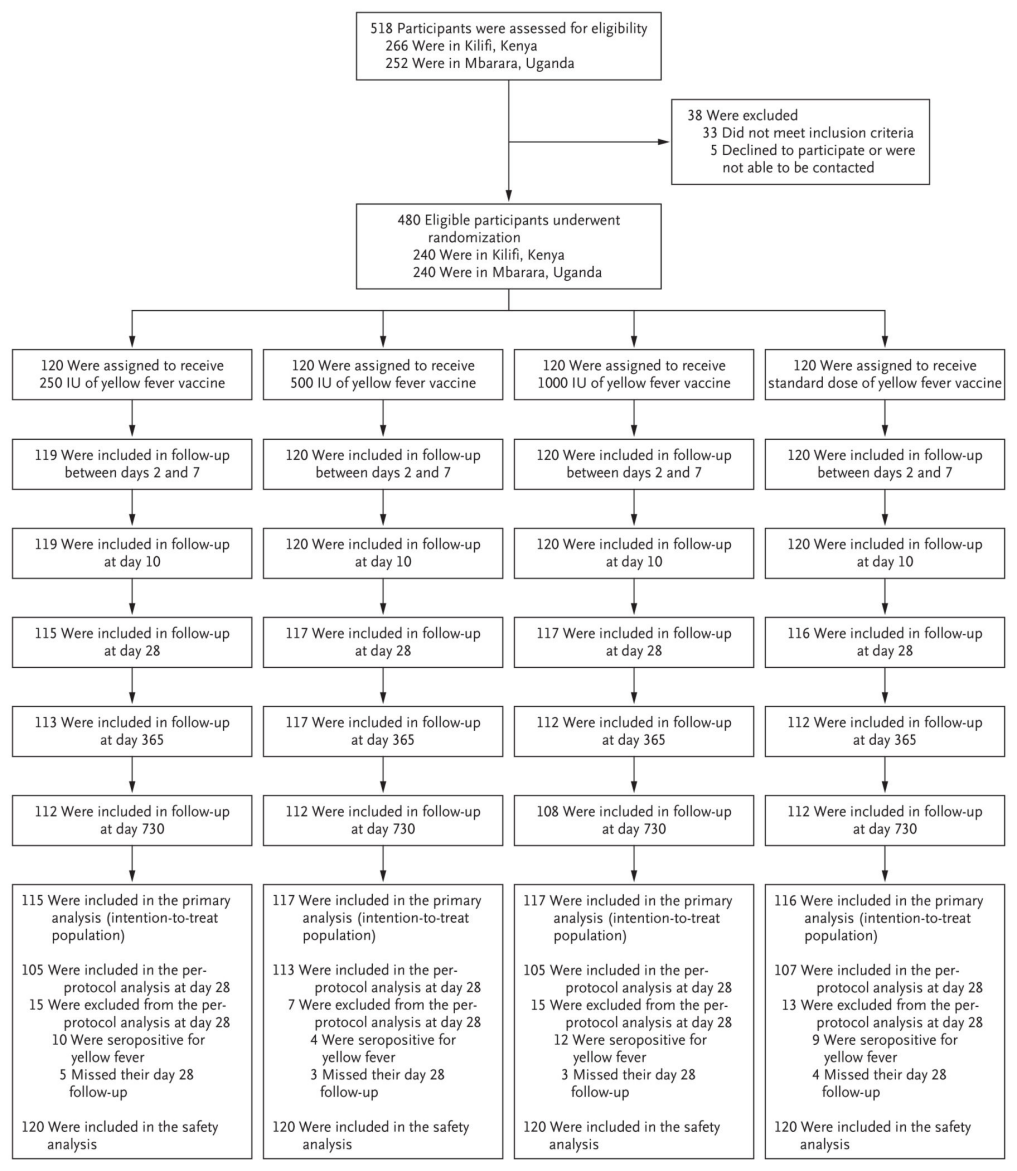
Screening, Enrollment, Randomization, Administration of Vaccine, and Follow-up.

The trial was conducted between November 11, 2019, and April 4, 2022. A total of 518 volunteers underwent screening; 33 were ineligible, 5 declined to participate, and 480 were enrolled and randomly assigned to receive a dose of yellow fever vaccine — a full standard dose of 13,803 IU (120 participants) or a fractionated dose of 1000 IU, 500 IU, or 250 IU (120 participants each) (Fig. 1). Of the total, 479 participants (approximately 100%) completed the follow-up visit on day 10, 465 participants (97%) completed the postvaccination visit on day 28, and 444 participants (93%) completed the last trial follow-up visit at day 730.

The primary intention-to-treat analysis included 115 participants in the 250-IU group, 117 in the 500-IU group, 117 in the 1000-IU group, and 116 in the standard-dose group. Missing outcome values in the intention-to-treat and per-protocol populations were the result of 15 missed visits (which accounted for 3% of the participants) randomly distributed across the four trial groups. Additional exclusions from the per-protocol population were attributed to prevaccination yellow fever antibody positivity (in 35 participants). This exclusion was for a prerandomization covariate that was not known at the time of randomization but which complicated the definition of seroconversion (the primary outcome).

The mean (±SD) age at enrollment was 39.7±11.5 years, the majority of the participants (296 [62%]) were women, and 38 (8%) were HIV-positive at baseline. The characteristics of the participants at baseline were similar across the trial groups (Table 1 and Table S6), and the participants were considered to be representative of the general adult population in the trial locations (Table S7).

### Immunogenicity

As of 28 days after vaccination, the incidence of seroconversion was higher than 90% (on a PRNT_50_ assay) in all four groups (Table 2). From day 28 onward, the consistent pattern was that the lowest level of seroconversion was observed in the 250-IU group; seroconversion was at similar levels in the 500-IU, 1000-IU, and standard-dose groups. However, seroconversion at 10 days was lower with the 500-IU and 1000-IU doses than with the standard dose. A similar pattern of results was seen in a comparison of geometric mean titers (Table 2).

**Figure 2:**
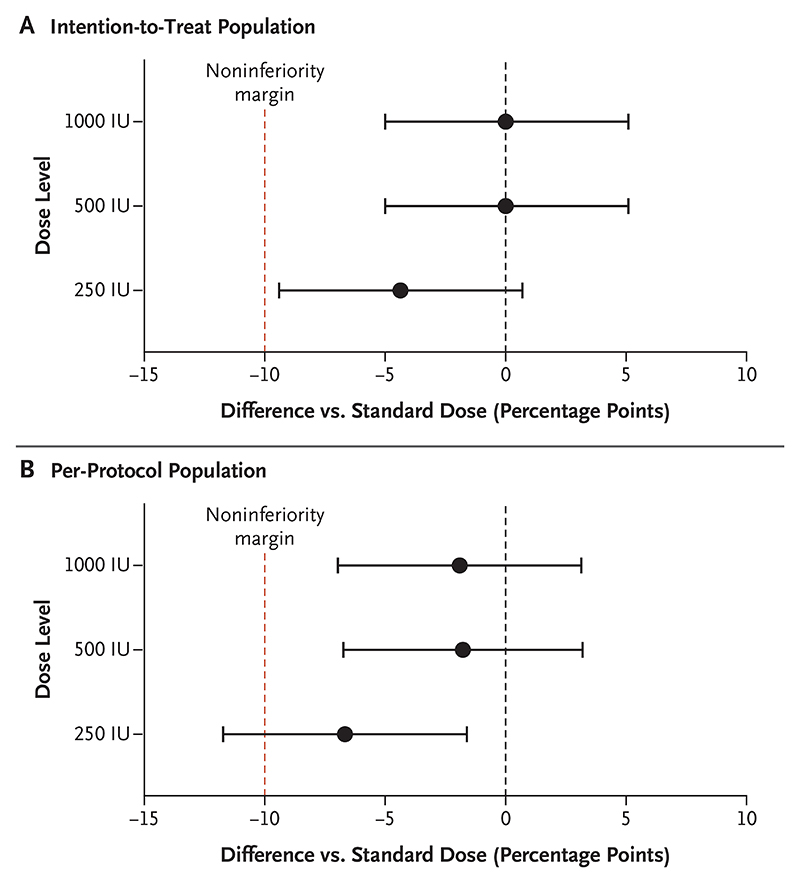
Seroconversion 28 Days after Vaccination with Fractional Doses of Yellow Fever Vaccine as Compared with Standard Dose. Shown are the differences in the incidence of seroconversion with fractional doses of 250 IU, 500 IU, and 1000 IU, as compared with the standard dose (13,803 IU), in the intention-to-treat population (Panel A) and the per-protocol population (Panel B). Seroconversion was defined as an antibody titer on a 50% plaque reduction neutralization assay that was at least four times as high as prevaccination titers. Horizontal bars indicate 95% confidence intervals.

The difference in the incidence of seroconversion between the factional doses and the standard dose at 28 days was −4.4 percentage points (95% CI, −9.4 to 0.7) with the 250-IU dose, and 0.01 percentage points (95% CI, −5.0 to 5.1) with both the 500-IU and 1000-IU doses in the intention-to-treat analysis (Fig. 2A and Fig. S1). Thus, noninferiority was met for the 250-IU, 500-IU, and 1000-IU doses. In the per-protocol analysis, differences in the incidence of seroconversion between the fractional doses and the standard dose were −1.9 percentage points (95% CI, −7 to 3.2) with the 1000-IU dose, −1.8 percentage points (95% CI, −6.7 to 3.2) with the 500-IU dose, and −6.7 percentage points (95% CI, −11.7 to 1.6) with the 250-IU dose (Fig. 2B). Therefore, the 250-IU dose did not meet the noninferiority criteria in the per-protocol analysis at 28 days. Similar conclusions were reached with the use of a PRNT_90_ assay in the intention-to-treat and the perprotocol populations (Tables S2 and S3).

Antibody responses in all the dose groups, as shown by geometric mean titers, peaked 28 days after vaccination (Fig. S2). Peak geometric mean titers were similar in the groups that received the standard dose, 1000-IU dose, or 500-IU dose but were lower in the group that received the 250-IU dose. Although the high geometric mean titers were maintained as of 365 days after vaccination, by 730 days after vaccination, geometric mean titers had decreased to between one fourth and one sixth of the peak titer value. The lowest geometric mean titers were recorded 10 days after vaccination. Results similar to those of the geometric mean titers were observed for geometric mean factor increases (Table 2). The geometric mean titers and geometric mean factor increases were consistently lower on a PRNT_90_ assay than on a PRNT_50_ assay (Fig. S2 and Table S2). Antibody responses in the per-protocol population were similar to those in the intention-to-treat population (Table S3).

Of the total trial population, 12 participants (2.5%; 95% CI, 1.3 to 4.3) were positive for yellow fever vaccine viremia between days 2 and 10 — one participant was positive on day 4, one on day 5, five on day 6, one on day 7, and four (one of whom had high-level viremia) on day 10 (Table S4). Geometric mean titers of subsequent responses on PRNT_50_ assays were higher among participants who previously had viremia (geometric mean titer ratios at 28 days, 2.1 [95% CI, 1.3 to 3.3]; at 365 days, 2.2 [95% CI, 0.9 to 5.2]; and at 730 days, 1.9 [95% CI, 0.8 to 4.5]) (Fig. S3). All the participants who had viremia were from Mbarara, and all had received fractional doses (Table S5). The risk of postvaccination viremia was higher among men than among women. There was no apparent association between viremia and vaccine dose or baseline characteristics (Table S5).

### Safety

A total of 546 adverse events in 274 participants were reported as of 28 days after vaccination — 66 (55%) of the participants in the 250-IU group reported 122 adverse events, 75 (63%) of the participants in the 500-IU group reported 163 adverse events, 68 (57%) of the participants in the 1000-IU group reported 136 adverse events, and 65 (54%) of the participants in the standard-dose group reported 125 adverse events (Table 3). Severe adverse events occurred in 3 participants (<1%; 1 in the 500-IU group and 2 in the 1000-IU group). The remaining participants with adverse events reported them as being mild (244 participants [51%]) or moderate (89 participants [19%]) in severity. The site investigators determined that 30 of 122 adverse events (25%) in the 250-IU group, 26 of 163 (16%) adverse events in the 500-IU group, 29 of 136 adverse events (21%) in the 1000-IU group, and 26 of 125 adverse events (21%) in the standard-dose group were related to the trial vaccine (Table 3).

The most common adverse events were headache (77 of 546 events [14%]), upper respiratory tract infection (44 [8%]), gastritis (43 [8%]), dizziness (41 [8%]), myalgia (28 [5%]), cough (21 [4%]), and fatigue (20 [4%]) (Table S9). There were 11 serious adverse events reported throughout the trial. These events included sinusitis, myocardial infarction, sepsis, malaria, cerebral hemorrhage, umbilical hernia, cerebrovascular accident, hypoglycemia, accidental death, and anemia (Table S10). A total of 10 of the 12 participants with viremia reported an adverse event; one participant with viremia (in the 1000-IU group) reported a serious adverse event but recovered without sequelae. The distribution of the adverse events was similar across viremia status (Table S8).

## Discussion

The primary outcome analysis showed that at 28 days after vaccination, doses of 500 IU and 1000 IU were noninferior to the standard vaccine dose (13,803 IU) with respect to seroconversion. The 250-IU dose met the noninferiority criterion in the intention-to-treat analyses, with a lower boundary for the difference of −9.4 percentage points (i.e., just above the noninferiority margin of −10 percentage points), but did not meet the noninferiority criterion in per-protocol analyses. The intention-to-treat analysis included participants with antibodies to yellow fever virus at baseline (35 participants), whereas the per-protocol analysis did not. The inclusion of participants with antibodies to yellow fever virus at baseline may have caused dilution of the estimates of noninferiority of the fractional dose, given the difficulties of determining seroconversion in such participants. Hence, we conclude that noninferiority criteria were shown for the 500-IU dose and not for the 250-IU dose, although the possible reduction in immunogenicity with the 250-IU dose may be considered to be acceptable in the context of an emergency outbreak response with substantial vaccine shortage.

The current recommended minimum dose is 1000 IU, but this recommendation was made on the basis of limited data obtained decades ago, and most vaccine doses in circulation are substantially in excess of this threshold. Previous data from Brazil (substrain 17DD) had indicated that immunogenicity is reduced at doses below 587 IU.^11^ However, these data were not formally analyzed for noninferiority and may not be generalizable to adults in Africa or vaccines from other substrains.

The findings from our trial provide support to the WHO recommendation that one fifth of a standard dose of vaccine be considered for use in epidemics, provided that the standard dose in the vaccine used is 500 IU or higher. Furthermore, vaccine manufacturers may consider it cost effective to reduce the potency of batches released and hence formally expand the vaccine stock that can be produced by existing manufacturing facilities. Based on the standard dose used in the present trial, this reduction in potency would have amounted to an increase in the number of doses by a factor of 27,^7^ and based on the lowest-potency vaccine stock tested in our previous trial, this would have amounted to an increase in stock by a factor of 13. However, allowances will need to be made for reductions in potency caused by storage, and hence further manufacturer development processes will be needed for determining an appropriate potency threshold for lyophilized doses.

For the potential gains in vaccine stock production to be fully realized, a reassessment of the long-standing process of releasing very-high-potency batches would be required. Criteria for the release of batches would need to be applied in a manner that does not create an unintended consequence of incentivizing very high potency, including a revised minimum potency, robust tests of potency, and appropriate tests for stability. Informal consultation that includes manufacturers, regulators, and researchers could inform updated guidelines. Furthermore, governments and international funders will need to confirm their willingness to expand emergency stock and routine vaccinations so that manufacturers receive support in maintaining the current production capacity of biologic material, investing in modifying practice, and scaling up the process of filling and packaging vials of vaccine for distribution. In addition, further clinical data are needed regarding the use of fractional doses in the pediatric population, as well as the longterm efficacy of the vaccine dose that is administered.

As in previous studies,^7–9^ our trial showed that lower doses led to considerably lower neutralization titers and lower levels of seroconversion at day 10, even though these differences were erased by the peak antibody responses seen at day 28. It is unlikely that these effects would be important in routine vaccination practice or in outbreak control, given that the intrinsic and extrinsic incubation periods are 5 days and 13 days, respectively. However, these factors may be of consideration in travel medicine, although seroconversion was 61% with a 500-IU dose as compared with 85% with a standard dose after 10 days, and this difference may be judged to be an acceptable risk given the short duration of the difference.^16^

Our trial showed that postvaccination viremia was infrequent, in contrast with somewhat higher proportions of viremia seen in other trials, ranging from 11% in studies in Brazil^17^ to 82% in studies in France.^18^ Factors that could explain the variability in prevalence include the frequency and timing of sampling, prevalence of HIV infection, and the substrain and dose of vaccine used.

Our trial has limitations, including with regard to statistical power. As with previous trials, our trial was powered to establish noninferiority at a potential reduction of 10 percentage points in the incidence of seroconversion at 28 days after vaccination, and this margin was informed by previous modeling studies of requirements for herd immunity.^16^ Nevertheless, we cannot exclude lower margins of noninferiority that may be relevant in some circumstances. Given recommendations that one dose be regarded as providing lifelong immunity, longer-term follow-up to determine the duration of responses is needed. Finally, our data may apply only to the 17D-204 vaccine substrain manufactured by Institut Pasteur de Dakar.

## Supplementary Material

appendix

Protocol

## Figures and Tables

**Table 1:** Characteristics of the Participants at Baseline.*

Characteristic	250-IU Dose (N = 120)	500-IU Dose (N = 120)	1000-IU Dose (N = 120)	Standard Dose (N = 120)
Age — yr	40.2±11.0	38.5±11.7	40.4±11.6	39.8±11.9
Sex — no. (%)				
Female	78 (65)	68 (57)	71 (59)	79 (66)
Male	42 (35)	52 (43)	49 (41)	41 (34)
Temperature — °C	36.4±0.5	36.4±0.5	36.3±0.4	36.4±0.5
Seropositive for yellow fever — no. (%)	10 (8)	4 (3)	12 (10)	9 (8)
Reported previous flavivirus infection — no. (%)†	0	0	0	0
Reported previous medical illness — no. (%)	48 (40)	54 (45)	44 (37)	56 (47)
HIV-positive — no. (%)†	8 (7)	12 (10)	5 (4)	13 (11)
Median CD4+ cell count (IQR) — cells/*μ*l	653 (533−712)	654 (428−884)	943 (709−951)	613 (480−758)

* Plus−minus values are means ±SD. The standard dose of yellow fever vaccine was 13,803 IU. Complete data for all baseline characteristics were available for all the participants. IQR denotes interquartile range.

† Of the total number of participants who were positive for human immunodeficiency virus (HIV) infection, 35 had World Health Organization (WHO) clinical stage 1 disease, and 3 had WHO clinical stage 2 disease.

**Table 2:** Seroconversion and Geometric Mean Titer in Fractional and Standard Doses of Yellow Fever Vaccine at Days 10, 28, 365, and 730 after Vaccination (Intention-to-Treat Population).*

Dose	Participants with Seroconversion	Incidence of Seroconversion (95% CI)	Difference vs. Standard Dose (95% CI)	GMT (95% CI)	GMT Ratio (95% CI)^†^
	*no./total no. vaccinated*	*percent*	*percentage points*		
Day 10					
250 IU	60/119	50 (42 to 59)	−34.6 (−48.7 to −20.5)	20.6 (15.5 to 27.4)	0.21 (0.13 to 0.33)
500 IU	73/120	61 (52 to 69)	−24.2 (−38.2 to −10.1)	30.1 (22.0 to 41.3)	0.30 (0.19 to 0.49)
1000 IU	73/120	61 (52 to 69)	−24.2 (−38.2 to −10.1)	36.1 (26.0 to 50.0)	0.36 (0.22 to 0.59)
Standard dose	102/120	85 (77 to 90)	Reference	99.6 (69.1 to 143.7)	Reference
Day 28					
250 IU	108/115	94 (88 to 97)	−4.4 (−9.4 to 0.7)	1303 (878 to 1934)	0.84 (0.52 to 1.35)
500 IU	115/117	98 (94 to 100)	0.01 (−5.0 to 5.1)	1773 (1337 to 2352)	1.14 (0.78 to 1.69)
1000 IU	115/117	98 (94 to 100)	0.01 (−5.0 to 5.1)	2182 (1661 to 2865)	1.41 (0.96 to 2.06)
Standard dose	114/116	98 (94 to 100)	Reference	1550 (1183 to 2030)	Reference
Day 365					
250 IU	104/113	92 (85 to 96)	−7.1 (−12.8 to −1.3)	1112 (753 to 1641)	0.88 (0.55 to 1.41)
500 IU	114/117	97 (92 to 99)	−1.7 (−7.4 to 4.0)	1556 (1148 to 2110)	1.24 (0.83 to 1.84)
1000 IU	109/112	97 (92 to 99)	−1.8 (−7.5 to 4.0)	1580 (1182 to 2112)	1.25 (0.85 to 1.85)
Standard dose	111/112	99 (95 to 100)	Reference	1259 (969 to 1636)	Reference
Day 730					
250 IU	94/112	84 (76 to 90)	−11.6 (−20.6 to −2.6)	221 (148 to 329)	0.61 (0.36 to 1.04)
500 IU	104/112	93 (86 to 97)	−2.7 (−11.7 to 6.3)	440 (299 to 648)	1.21 (0.72 to 2.05)
1000 IU	98/108	91 (84 to 95)	−4.8 (−13.9 to 4.3)	373 (254 to 549)	1.03 (0.61 to 1.74)
Standard dose	107/112	96 (90 to 98)	Reference	362 (254 to 517)	Reference

* Antibody titers were determined with the use of 50% plaque reduction neutralization assays. At each time point, seroconversion was defined as a neutralizing antibody titer that was at least four times as high as that at baseline. The widths of the confidence intervals were not adjusted for multiplicity and may not be used in place of hypothesis testing except for the day-28 and safety outcomes.

† The geometric mean titer (GMT) ratio is calculated by dividing the GMT of the fractional dose by the GMT of the standard dose.

**Table 3:** Summary of All Adverse Events.*

Variable	250-IU Dose (N = 120)	500-IU Dose (N = 120)	1000-IU Dose (N = 120)	Standard Dose (N = 120)	Total (N = 480)
Adverse events					
No. reported	122	163	136	125	546
Participants with adverse event — no. (%)†	66 (55)	75 (62)	68 (57)	65 (54)	274 (57)
Serious adverse events					
No. reported	2	0	5	4	11
Participants with serious adverse events — no. †	2	0	5	4	11
Severity — no./total no. of events (%)					
Mild	92/122 (75)	120/163 (74)	105/136 (77)	98/125 (78)	415/546 (76)
Moderate	30/122 (25)	42/163 (26)	29/136 (21)	27/125 (22)	128/546 (23)
Severe	0	1/163 (1)	2/136 (1)	0	3/546 (1)
Life-threatening	0	0	0	0	0
Severity — no. of participants (%)‡					
Mild	62 (52)	63 (52)	60 (50)	59 (49)	244 (51)
Moderate	19 (16)	31 (26)	19 (16)	20 (17)	89 (19)
Severe	0	1 (1)	2 (2)	0	3 (1)
Life-threatening	0	0	0	0	0
Adverse event related to vaccination — no./total no. of events (%)§					
Related	30/122 (25)	26/163 (16)	29/136 (21)	26/125 (21)	111/546 (20)
Probably related	15/122 (12)	25/163 (15)	17/136 (12)	13/125 (10)	70/546 (13)
Not related	77/122 (63)	112/163 (69)	90/136 (66)	86/125 (69)	365/546 (67)

* Adverse events were reported throughout the duration of the trial.

† Participants who had one or more adverse events or serious adverse events were counted only once.

‡ Participants were counted only once within each severity grade or relatedness category.

§ The relatedness of an adverse event to trial vaccination was assessed by the site investigators.
