# Supplementary material for: Low-Dose Yellow Fever Vaccine in Adults in Africa
Source: N Engl J Med. Author manuscript; Available in PMC 2025 Mar 9. (PMC7617464; doi:10.1056/NEJMoa2407293)
Supplement: appendix [file EMS203456-supplement-appendix.pdf]

Supplementary Appendix

Supplement to: Kimathi D, Juan-Giner A, Bob NS, et al. Low-dose yellow fever vaccine in adults in Africa. N Engl J Med 2025;392:788-97. DOI: 10.1056/NEJMoa2407293

This appendix has been provided by the authors to give readers additional information about the work.

Supplementary Appendix

Low-Dose Yellow Fever Vaccine in Adults in Africa

Derick Kimathi, PhD[1*], Aitana Juan-Giner, MSc[2*], Ndeye S. Bob, PhD[3*], Benedict Orindi, PhD[1], Maria L. Namulwana, MBChB[4], Antoine Diatta, PharmD[3], Stanley Cheruiyot, BSc[1], Gamou Fall, PhD[3], Moussa Dia, MSc[3], Mainga M. Hamaluba, MD[1], Dan Nyehangane, MSc[4], Henry K. Karanja, MSc[1], John N. Gitonga, BSc[1], Daisy Mugo, BSc[1], Donwilliams O. Omuoyo, BSc[1], Mwatasa Hussein, MSc[1], Elizaphan Oloo, Dip[1], Naomi Kamau, BSc[1], Jackline Wafula, MSc[1], Josephine Bendera, KCSE[1], Namanya Silvester, MBChB[4], James Mwavita, KCSE[1], Musiimenta Joshua, BNS[4], Jane Mwendwa, BSc[1], Collins Agababyona, MA[4], Caroline Ngetsa, MSc[1], Nalusaji Aisha, BMLS[4], Felix Moki, Dip[1], Titus Buluku, Dip[1], Marianne Munene, MSc[1], Juliet Mwanga-Amumpaire, PhD[4], Julius Lutwama, PhD[5], John Kayiwa, MSc[5], Eunice Kamaara, PhD[6], Alan D. Barrett, PhD[7], Pontiano Kaleebu, PhD[5,8], Philip Bejon, PhD[1,9], Amadou A. Sall, PhD[3], Rebecca F. Grais, PhD[2,], George M. Warimwe, PhD[1,9,**] for the NIFTY team

[1] Kenya Medical Research Institute (KEMRI) – Wellcome Trust Research Programme, Kilifi, Kenya; [2] Epicentre, Paris, France; [3] Institut Pasteur Dakar, Senegal; [4] Epicentre Mbarara Research Centre, Uganda; [5] Uganda Virus Research Institute (UVRI), Entebbe, Uganda; [6] Moi University, Eldoret, Kenya; [7] Sealy Institute for Vaccines Sciences and Department of Pathology, University of Texas Medical Branch, Galveston, TX, USA; [8] Medical Research Council-UVRI and The London School of Hygiene & Tropical Medicine, Uganda Research Unit, Entebbe, Uganda; [9] Nuffield Department of Medicine, University of Oxford, UK

Table of Contents

Supplementary Methods 3

Figure S1. Non-inferiority of seroconversion of fractional doses compared with standard dose for intention-to-treat population (PRNT50) at days 10, 28, 365 and 730 post-vaccination.........6

Figure S2. Intention-to-treat analysis: Geometric mean titers by PRNT50 (A) and PRNT90 (B) are shown by dose level. 7

Figure S3. $PRNT_{50}$ titers for viremic and non-viremic participants. $PRNT_{50}$ titers for participants who were viremic or non-viremic between days 2 to 10 postvaccination are shown. Horizontal lines represents geometric means. 8

Table S1: Back-titration of vaccine potency following reconstitution of lyophilized vaccine as per manufacturer's instructions 9

Table S2. Intention-to-treat analysis: Seroconversion and geometric mean fold increase by $PRNT_{90}$ in fractional and standard doses of yellow fever vaccine at days 10, 28, 365 and 730 post-vaccination. 10

Table S3. Per-protocol analysis: Seroconversion and geometric mean fold increase by $PRNT_{50}$ in fractional and doses of yellow fever vaccine at days 10, 28, 365 and 730 post-vaccination. 12

Table S4. Number tested, number viremic and their respective viremia levels........................14

Table S5. Association of viremia status with dose and participant characteristics..................15

Table S6. Baseline characteristics for the per-protocol population 16

Table S7. Representativeness of the study participants 17

Table S8. Distribution of adverse events by viremia status...18

Table S9. Adverse events (preferred terms) by study group listed from the most common 19

Table S10. Serious adverse events 22

References 23

Supplementary Methods

Vaccine preparation and procedures

The potency of vaccine batches produced by Institut Pasteur de Dakar ranges between 3.50 to 5.10 log_{10} IU/dose. A single lot of standard lyophilized 10-dose vaccine vials, containing 13803 IU per dose, was used. The lot used for this trial was at the median potency (4.14 log_{10} IU/dose i.e. 13803 IU/dose) of batches produced by the manufacturer. The lyophilized product was reconstituted in vaccine diluent to produce the various dose levels as per manufacturer's instructions (see Table S1). This process was masked from the study team, and handled by the unblinded team, which included vaccinating nurses and a vaccination supervisor (pharmacist and the senior nurse) who had no further role in the study. Lyophilized 10-dose vaccine vials of the same lot of vaccine were also shipped to the UK National Institute for Biological Standards and Control (NIBSC), a WHO Collaborating Centre, who independently confirmed, by back-titration, that the manufacturer's vaccine reconstitution instructions resulted in the intended dose levels for the study (Table S1). The back-titrations at NIBSC were done while participant enrolment at the study sites was ongoing. An independent medical monitor had access to unblinded data throughout the trial. The prepared vaccines were labelled and kept at 2-8°C until administration and discarded after six hours from the time of dilution, if not used. All the vaccine doses were administered subcutaneously as 0.5mls at a 45° injection angle using auto-disable syringes with a 25G × 3/4" needle size.

Sample size considerations

Different approaches have been taken to fixing non-inferiority margins, with many trials arbitrarily using 5% or 10% [1]. The United States Food and Drug Administration guidance is that the margin includes a clinical judgement on acceptable differences [2], and here we based our decision on a modelling study that assessed the public health consequence of reductions in seroconversion rate to select a margin that would be consistent with preserved herd immunity [3]. Sample size calculation was performed using *art2bin* on Stata version 15 (StataCorp, College Station,TX) and cross-checked using Power Analysis & Sample Size software (PASS) version 14. Sample size

calculation and simulations based on these assumptions yielded a sample size of 100 participants per arm and we increased this by 20% to account for participants lost to follow-up, any participants whose results would be unevaluable and participants with pre-existing antibodies against YF virus at baseline. Thus, we used a total sample size of 480 for the four vaccine dose groups. No adjustment for multiplicity of testing was a-priori specified for outcomes other than the 28-day outcome.

Statistical analysis

The primary outcome analysis was a two-group comparison of the rate of seroconversion, defined as a four-fold or greater rise in $PRNT_{50}$ titer between day 0 and day 28 samples, for each of the lower doses and the standard dose. Any PRNT value below the Limit of Quantification (LOQ) (i.e., <1:10) was converted to half the LOQ. Thus a 4 -fold increase for a subject who was negative (i.e. below 1:10) at baseline, is a titer of 20. The highest serial dilution was 20480. Titers above 1:20480 were designated as >20480. We summarized the number and percentage of participants seroconverting together with their associated Agresti-Coull 95% confidence intervals [4]. A non-inferiority test using Dunnett's test was performed for the difference in seroconversion rates in the standard dose and each of the lower doses using two-sided 95% CIs. Non-inferiority was defined as a lower bound of the 95% CI for the difference in seroconversion rate being greater than −10%. Secondary outcome analyses included using $PRNT_{90}$ GMTs in the ITT and PP populations. The GMT, GMFI, and GMT ratios and GMFI ratios of fractional doses to the standard dose were estimated at days 10, 28, 365 and 730 after vaccination. We assessed the association of viremia status with dose level and baseline characteristics (listed in Table 1) using an exact Poisson regression analysis as we experienced computational challenges with a log-binomial model [5]. We also compared GMTs between viremia status at days 28 and 365 using Welch's t-test on log-transformed titers.

Safety data for all adverse events up to 28 days post-vaccination were summarized as numbers and as proportions, and were presented according to whether they were related, probably related or not related to vaccination and by YF vaccine dose group. They were also presented according to severity. The proportion of participants in each vaccine dose group reporting any local reaction

were compared using the chi-squared test. All serious adverse events were described in detail for each participant. The widths of the confidence intervals were not adjusted for multiplicity and may not be used in place of hypothesis testing except for the primary and safety outcomes. Generally, our study had acceptably low attrition rates with only 3% of participants [6] missing day 28 visits for unknown reasons. There were no missing values for baseline characteristics and covariates. Together with the likelihood-based regression, our findings are assumed valid under the missing at random mechanism [7]. All analyses were performed using Stata version 15 (StataCorp, College Station, TX). Plots were generated using GraphPad Prism version 9.4.0 (GraphPad Software, San Diego, California USA).

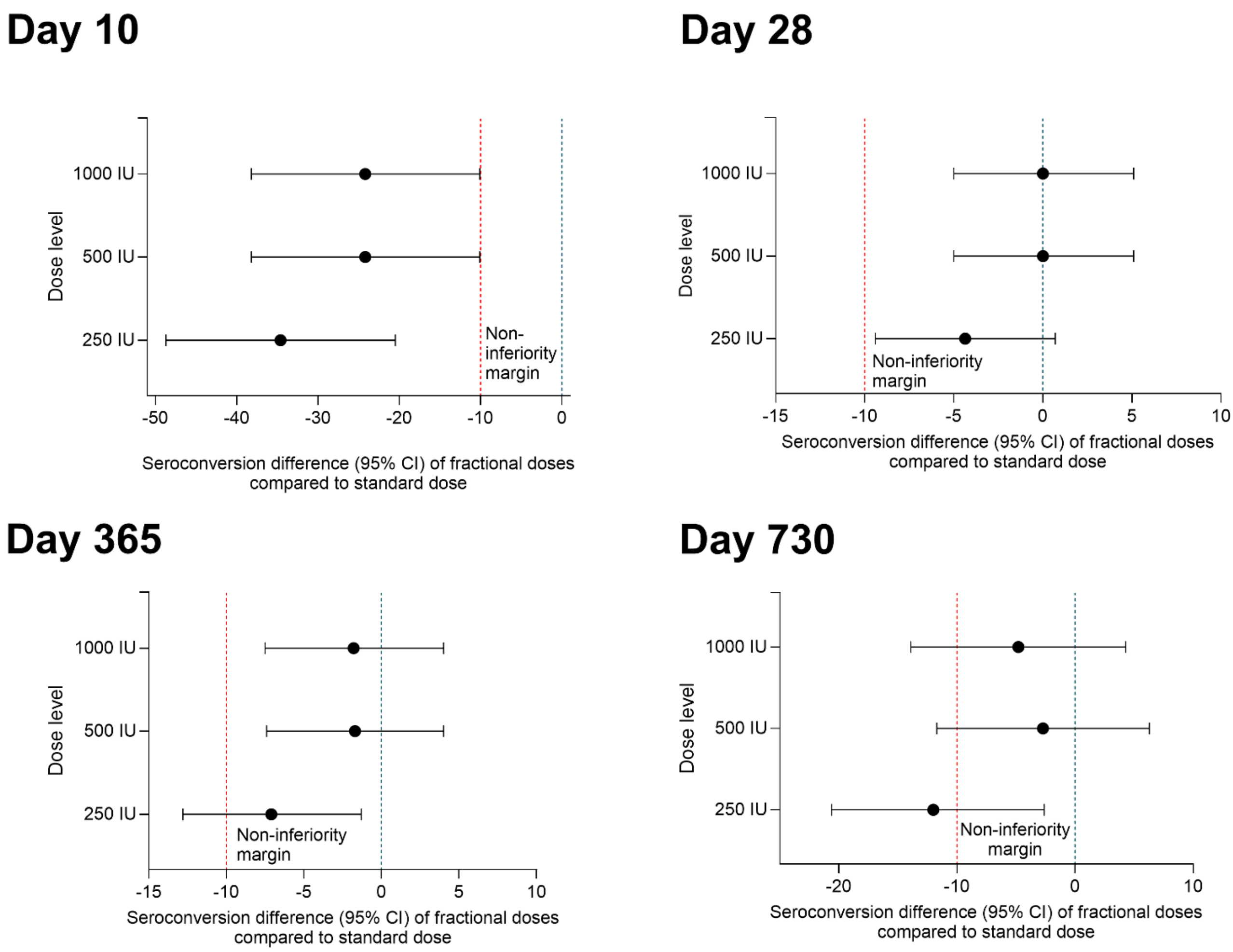

Figure S1. Non-inferiority of seroconversion of fractional doses compared with standard dose for intention-to-treat population (PRNT50) at days 10, 28, 365 and 730 post-vaccination. The widths of the confidence intervals were not adjusted for multiplicity and may not be used in place of hypothesis testing except for the Day 28 and safety outcomes.

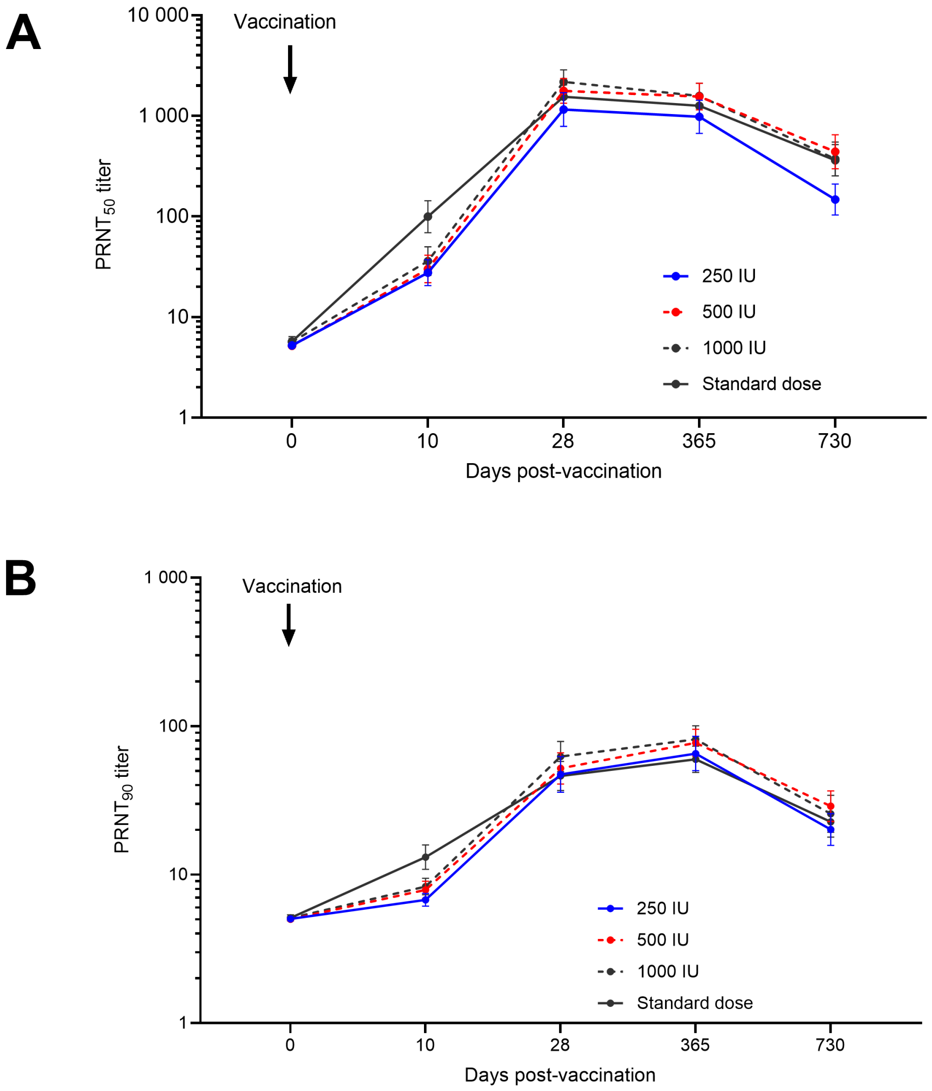

Number in ITT analysis

250 IU	120	119	115	113	112
500 IU	120	120	117	117	112
1000 IU	120	120	117	112	108
Standard dose	120	120	116	112	112

Figure S2. Intention-to-treat analysis: Geometric mean titers by PRNT50 (A) and PRNT90 (B) are shown by dose level. The bars are 95% confidence intervals. The widths of the confidence intervals were not adjusted for multiplicity and may not be used in place of hypothesis testing except for the Day 28 and safety outcomes.

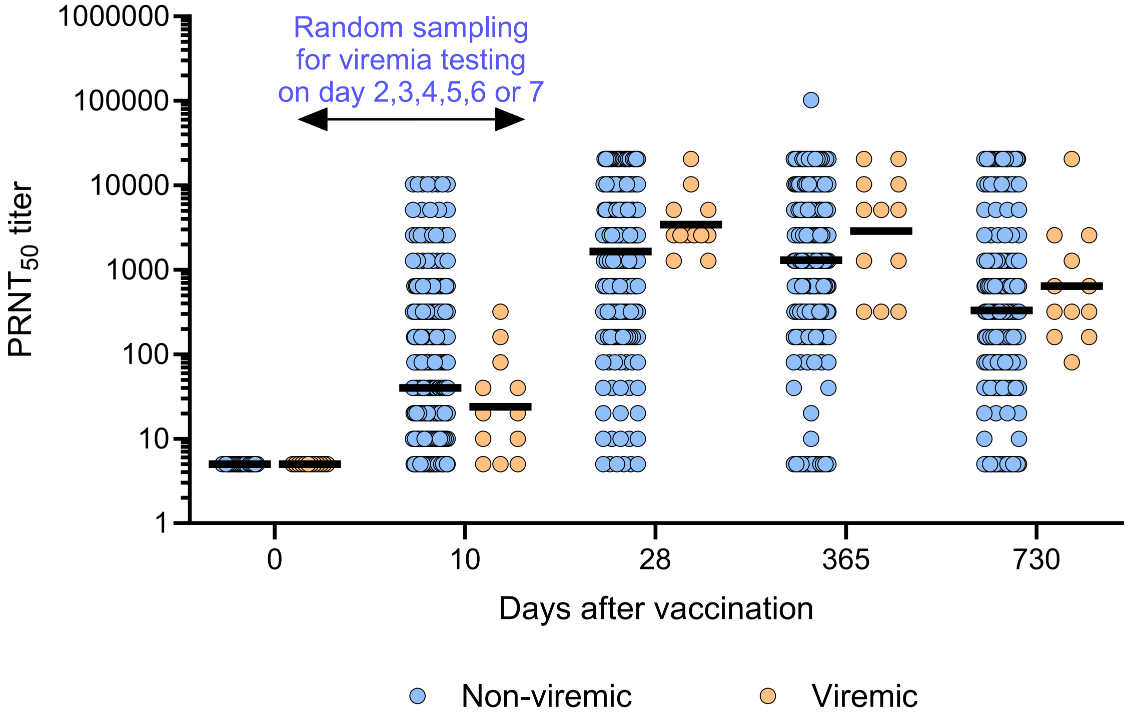

Figure S3. PRNT$_{50}$ titers for viremic and non-viremic participants. PRNT$_{50}$ titers for participants who were viremic or non-viremic between days 2 to 10 postvaccination are shown. Horizontal lines represents geometric means.

Table S1: Back-titration of vaccine potency following reconstitution of lyophilized vaccine as per manufacturer's instructions

Group allocation	IPD results		NIBSC results	
	IU/dose	LogIU/dose	IU/dose	LogIU/dose
**Standard dose**	13803	4.14	19498	4.29
**1000 IU**	1022	3.01	1022	3.01
**500 IU**	502	2.70	457	2.66
**250 IU**	251	2.40	235	2.37

All the vaccine doses were administered subcutaneously as 0.5mls. A single lyophilized vaccine lot was used for this study. The table shows the potencies following back-titration of the reconstituted vaccine by group allocation. The standard dose was made by reconstituting the lyophilized product in 5ml vaccine diluent, as per manufacturer's instructions. To make 1000 IU per dose, 0.5ml of the reconstituted standard vaccine vial was added to 6.25ml of vaccine diluent. This resulted in 1000 IU per 0.5ml dose. To make 500 IU per dose, 0.2ml of the reconstituted standard vaccine vial was added to 5.30ml of vaccine diluent. This resulted in 500 IU per 0.5ml dose. To make 250 IU per dose, 0.1ml of the reconstituted standard vaccine vial was added to 5.40ml of vaccine diluent. This resulted in 250 IU per 0.5ml dose. IPD - Institut Pasteur Dakar; NIBSC - UK National Institute for Biological Standards and Control.

Table S2. Intention-to-treat analysis: Seroconversion and geometric mean fold increase by $PRNT_{90}$ in fractional and standard doses of yellow fever vaccine at days 10, 28, 365 and 730 post-vaccination.

Dose level	Total (n)	Seroconversion*			Geometric mean titer (95% CI)**	Geometric mean titer ratio (fractional: standard)**	Geometric mean fold increase in titer (95% CI)**	Geometric mean fold increase in titer ratio: fractional to standard (95% CI)**
		n	% (95% CI)**	Difference (fractional-standard)**				
Day 10								
250 IU	119	11	9 (5 to 16)	-25.76 (-37.41 to -14.1)	6.8 (6.1 to 7.5)	0.52 (0.42 to 0.64)	1.3 (1.2 to 1.5)	0.52 (0.43 to 0.65)
500 IU	120	21	18 (12 to 25)	-17.5 (-29.13 to -5.87)	7.9 (6.9 to 9)	0.60 (0.48 to 0.76)	1.6 (1.4 to 1.8)	0.62 (0.49 to 0.77)
1000 IU	120	18	15 (10 to 23)	-20 (-31.63 to -8.37)	8.3 (7.3 to 9.4)	0.63 (0.51 to 0.79)	1.6 (1.4 to 1.9)	0.64 (0.51 to 0.8)
Standard dose	120	42	35 (27 to 44)	Reference	13.1 (10.9 to 15.8)	Reference	2.6 (2.1 to 3.1)	Reference
Day 28								
250 IU	115	87	76 (67 to 83)	-6.24 (-17.92 to 5.43)	47 (36 to 62)	1.03 (0.72 to 1.46)	9 (7 to 12)	1.04 (0.73 to 1.49)
500 IU	117	97	83 (75 to 89)	1.01 (-10.61 to 12.63)	52 (41 to 66)	1.12 (0.81 to 1.56)	10 (8 to 13)	1.15 (0.83 to 1.6)
1000 IU	117	105	90 (83 to 94)	7.85 (-3.77 to 19.47)	62 (49 to 79)	1.35 (0.98 to 1.86)	12 (10 to 16)	1.36 (0.98 to 1.88)
Standard dose	116	95	82 (74 to 88)	Reference	46 (37 to 58)	Reference	9 (7 to 11)	Reference
Day 365								
250 IU	113	96	85 (77 to 90)	-5.22 (-14.26 to 3.82)	65 (50 to 85)	1.09 (0.78 to 1.52)	13 (10 to 17)	1.11 (0.8 to 1.56)
500 IU	117	109	93 (87 to 97)	2.98 (-5.98 to 11.95)	77 (63 to 95)	1.29 (0.97 to 1.72)	15 (13 to 19)	1.32 (0.99 to 1.77)
1000 IU	112	106	95 (89 to 98)	4.46 (-4.6 to 13.52)	81 (66 to 101)	1.36 (1.02 to 1.82)	16 (13 to 20)	1.37 (1.02 to 1.84)
Standard dose	112	101	90 (83 to 95)	Reference	60 (49 to 73)	Reference	12 (9 to 14)	Reference

Day 730								
250 IU	112	64	57 (48 to 66)	-3.57 (-18.79 to 11.64)	20 (16 to 26)	0.89 (0.63 to 1.24)	4 (3 to 5)	0.90 (0.64 to 1.27)
500 IU	112	77	69 (60 to 77)	8.04 (-7.18 to 23.25)	29 (23 to 37)	1.28 (0.92 to 1.78)	6 (5 to 7)	1.31 (0.93 to 1.83)
1000 IU	108	68	63 (54 to 71)	2.25 (-13.11 to 17.6)	26 (19 to 34)	1.13 (0.79 to 1.64)	5 (4 to 7)	1.14 (0.79 to 1.65)
Standard dose	112	68	61 (51 to 69)	Reference	23 (18 to 29)	Reference	4 (3 to 6)	Reference

*Seroconversion is defined as a ≥4-fold increase in neutralizing antibody titer at each timepoint from baseline. ** The widths of the confidence intervals were not adjusted for multiplicity and may not be used in place of hypothesis testing except for the Day 28 and safety outcomes.

Table S3. Per-protocol analysis: Seroconversion and geometric mean fold increase by $PRNT_{50}$ in fractional and doses of yellow fever vaccine at days 10, 28, 365 and 730 post-vaccination.

Dose level	Total (n)	Seroconversion*			Geometric mean titer (95% CI)**	Geometric mean titer ratio (fractional: standard)**	Geometric mean fold increase in titer (95% CI)**	Geometric mean fold increase in titer ratio: fractional ÷ standard (95% CI)**
		n	% (95% CI)**	Difference (fractional-standard)**				
Day 10								
250 IU	109	57	52 (43 to 62)	-35.57 (-50.02 to -21.12)	20.3 (15 to 27.4)	0.19 (0.12 to 0.31)	4.1 (3 to 5.5)	0.19 (0.12 to 0.31)
500 IU	116	71	61 (52 to 70)	-26.18 (-40.44 to -11.92)	30.6 (22.2 to 42.2)	0.29 (0.17 to 0.47)	6.1 (4.4 to 8.4)	0.29 (0.17 to 0.47)
1000 IU	108	69	64 (54 to 72)	-23.5 (-38.01 to -8.98)	36.6 (25.6 to 52.3)	0.34 (0.20 to 0.58)	7.3 (5.1 to 10.5)	0.34 (0.2 to 0.58)
Standard dose	111	97	87 (80 to 92)	Reference	106.6 (72.8 to 156.1)	Reference	21.3 (14.6 to 31.2)	Reference
Day 28								
250 IU	105	98	93 (87 to 97)	-6.67 (-11.72 to -1.61)	1190 (781 to 1813)	0.72 (0.44 to 1.18)	238 (156 to 363)	0.72 (0.44 to 1.18)
500 IU	113	111	98 (93 to 100)	-1.77 (-6.73 to 3.2)	1861 (1400 to 2473)	1.13 (0.77 to 1.66)	372 (280 to 495)	1.13 (0.77 to 1.66)
1000 IU	105	103	98 (93 to 100)	-1.9 (-6.96 to 3.15)	2171 (1615 to 2917)	1.32 (0.89 to 1.96)	434 (323 to 583)	1.32 (0.89 to 1.96)
Standard dose	107	107	100 (96 to 100)	Reference	1648 (1262 to 2153)	Reference	330 (252 to 431)	Reference
Day 365								
250 IU	104	95	91 (84 to 96)	-8.65 (-14.65 to -2.66)	994 (657 to 1502)	0.79 (0.48 to 1.29)	199 (131 to 300)	0.79 (0.48 to 1.29)
500 IU	113	110	97 (92 to 99)	-2.65 (-8.53 to 3.22)	1577 (1160 to 2143)	1.25 (0.83 to 1.88)	315 (232 to 429)	1.25 (0.83 to 1.88)
1000 IU	101	98	97 (91 to 99)	-2.97 (-9.01 to 3.07)	1562 (1142 to 2137)	1.23 (0.81 to 1.87)	312 (228 to 427)	1.23 (0.81 to 1.87)
Standard dose	103	103	100 (96 to 100)	Reference	1266 (956 to 1675)	Reference	253 (191 to 335)	Reference
Day 730								

250 IU	102	84	82 (74 to 89)	-14.76 (-23.63 to -5.9)	206 (134 to 316)	0.56 (0.32 to 0.98)	41 (27 to 63)	0.56 (0.32 to 0.98)
500 IU	109	102	94 (87 to 97)	-3.54 (-12.26 to 5.18)	441 (300 to 648)	1.21 (0.71 to 2.04)	88 (60 to 130)	1.21 (0.71 to 2.04)
1000 IU	97	91	94 (87 to 97)	-3.3 (-12.28 to 5.68)	388 (263 to 573)	1.06 (0.63 to 1.8)	78 (53 to 115)	1.06 (0.63 to 1.8)
Standard dose	104	101	97 (91 to 99)	Reference	366 (254 to 527)	Reference	73 (51 to 105)	Reference

*Seroconversion is defined as a ≥4-fold increase in neutralizing antibody titer at each timepoint from baseline. **The widths of the intervals have not been adjusted for multiplicity and the intervals may not be used in place of hypothesis testing for all outcomes other than for the Day 28 outcome for the primary analysis and safety outcomes.

Table S4. Number tested, number viremic and their respective viremia levels

Day	Number tested*	Number viremic	Viral load (copies/μL)
0	480	0	
2	80	0	
3	80	0	
4	80	1	2175
5	79	1	245
6	80	5	390; 608; 1800; 2500; 7912
7	80	1	3372
10	479	4	1531; 3073; 129710; 1469994

*One participant missed day 5 and day 10 visits (see Figure 1)

Table S5. Association of viremia status with dose and participant characteristics

Covariates	Number of participants	Number viremia positive	Risk ratio (95% CI)
Dose			
250 IU	120	3	Reference
500 IU	120	5	1.67 (0.38–8.47)
1000 IU	120	4	1.33 (0.28–7.15)
Standard dose	120	0	0.26 (0–1.71)
Site			
Kilifi	240	0	0.06 (0–0.28)
Mbarara	240	12	Reference
Sex			
Female	296	3	Reference
Male	184	9	4.83 (1.36–22.1)
Age at enrolment in years	480	12	1.02 (0.97–1.08)
Yellow Fever serostatus at baseline			
Negative	445	12	Reference
Positive	35	0	0.76 (0–3.61)
Reported previous medical illness			
No	278	9	Reference
Yes	202	3	0.46 (0.10–1.62)
HIV status at baseline			
Negative	442	11	Reference
Positive	38	1	1.06 (0.05–6.19)

Table S6. Baseline characteristics for the per-protocol population

Characteristic	250 IU	500 IU	1000 IU	Standard Dose
Number in primary per-protocol population	105	113	105	107
Mean age at enrolment (SD), years	40.6 (10.8)	38.4 (11.9)	39.7 (11.7)	39.6 (12)
Sex				
Female	69 (66%)	66 (58%)	62 (59%)	69 (64%)
Male	36 (34%)	47 (42%)	43 (41%)	38 (36%)
Temperature, ºC, mean (SD)	36.4 (0.5)	36.4 (0.5)	36.3 (0.4)	36.4 (0.5)
Seropositive to YF virus at baseline, n (%)*	_	_	_	_
Reported previous flavivirus infection, n (%)	0 (0%)	0 (0%)	0 (0%)	0 (0%)
Reported previous medical illness, n (%)	45 (43%)	52 (46%)	36 (34%)	50 (47%)
HIV positive at baseline, n (%)	7 (7%)	11 (10%)	5 (5%)	12 (11%)
CD4 cell count, median (IQR)	634 (495 ,713)	630 (400 ,894)	943 (709 ,951)	602 (436 ,762)

*Participants who were seropositive to YF virus were excluded from the per-protocol population.

Table S7. Representativeness of the study participants

Category	Detail
**Disease, problem, or condition under investigation**	Yellow fever (YF)
**Special considerations related to**	
**Age**	Individuals of all ages are susceptible to YF [8,9]. During 2023 to February 2024 individuals affected by YF had a mean age of 25 years of age, with 69% aged over 15 years old [10].
**Sex and gender**	It is likely that the transmission cycles seen in Africa put at risk all unprotected individuals, and that imbalances in sex are related to reporting differences. For instance, females were overrepresented in the YF outbreaks reported during 2023 in Africa [10] but a predominance of cases in males has also been observed in other outbreaks [9].
**Geography**	Over 90% of reported YF cases and deaths tend to be in Africa where the disease is endemic in more than 30 countries, including Kenya and Uganda [11].
**Other considerations**	YF surveillance in Africa is suboptimal, meaning that the 130,000 cases and 78,000 thought to occur annually may be an underestimate [11]. Preventive YF vaccination is highly effective and is the primary control strategy recommended by WHO to eliminate YF epidemics [10].
**Overall representativeness of this trial**	The participants enrolled in this study were recruited from the general population in two African countries prone to YF outbreaks. The low baseline seropositivity for YF antibodies suggests the adult population in the trial sites were susceptible to YF infection. WHO recommends routine vaccination of children in all countries in which YF is endemic. When vaccine coverage is not sustained, YF infection recurs, potentially leading to major outbreaks. Emergency mass preventive vaccination campaigns may target both children and adults. Hence, the participants included in the trial were representative of a population that would be targeted by a preventive vaccination campaign.

Table S8. Distribution of adverse events by viremia status

Adverse events	Viremia status		
	Negative	Positive	Total
**Number of participants**	468	12	480
**Number of AEs**	524	22	546
**Number of AEs by severity***			
Mild	399 (76%)	16 (73%)	415 (76%)
Moderate	123 (23%)	5 (23%)	128 (23%)
Severe	2 (<1%)	1 (5%)	3 (1%)
**Number of AEs by relationship to study product***			
Related	106 (20%)	5 (23%)	111 (20%)
Probably related	65 (12%)	5 (23%)	70 (13%)
Not related	353 (67%)	12 (55%)	365 (67%)

*Percentages are based on number of AEs reported for each group.

Table S9. Adverse events (preferred terms) by study group listed from the most common

	Dose level				
Preferred term	250 IU	500 IU	1000 IU	Standard	Total
Headache	17	21	27	12	77
Upper Respiratory Tract Infection	14	8	14	8	44
Gastritis	4	17	11	11	43
Dizziness	9	9	9	14	41
Myalgia	4	10	10	4	28
Cough	4	7	6	4	21
Fatigue	5	2	6	7	20
Nasopharyngitis	4	5	2	4	15
Rhinorrhoea	1	8	1	5	15
Arthralgia	3	4	2	5	14
Vulvovaginal Candidiasis	3	6	2	3	14
Neuropathy Peripheral	3	3	4	2	12
Urinary Tract Infection	5	3	2	2	12
Back Pain	3	1	4	2	10
Pyrexia	0	4	3	2	9
Malaria	2	3	1	2	8
Nausea	2	1	4	1	8
Body Tinea	2	2	2	0	6
Gastroenteritis	1	2	1	2	6
Soft Tissue Injury	1	2	1	1	5
Pregnancy	1	1	0	3	5
Rash	3	1	1	0	5
Gastrooesophageal Reflux Disease	1	2	1	1	5
Tonsillitis	0	1	1	3	5
Conjunctivitis Allergic	3	2	0	0	5
Abdominal Pain	0	3	2	0	5
Tinnitus	1	2	1	0	4
Pharyngitis	0	1	1	1	3
Paraesthesia	2	1	0	0	3
Toothache	0	1	0	2	3
Vision Blurred	0	1	0	2	3
Decreased Appetite	1	2	0	0	3
Dermatitis Atopic	1	2	0	0	3
Pelvic Inflammatory Disease	1	1	0	1	3
Furuncle	2	0	0	0	2
Oral Herpes	0	1	1	0	2

Low Back Pain	1	0	0	1	2
Genital Herpes	0	0	1	1	2
Diarrhoea	0	1	0	1	2
Hypersensitivity	0	2	0	0	2
Chills	1	1	0	0	2
Pruritus	1	0	1	0	2
Lower Abdominal Pain	1	0	0	1	2
Common Cold	2	0	0	0	2
Abscess	0	0	1	1	2
Hypertension	0	0	2	0	2
Vomiting	0	1	0	1	2
Gingivitis	0	0	1	0	1
Torticollis	0	0	1	0	1
Skin Laceration	0	0	0	1	1
Increased Appetite	1	0	0	0	1
Pollakiuria	0	1	0	0	1
Injection Site Reaction	0	0	0	1	1
Rhinitis Allergic	0	1	0	0	1
Aphthous Ulcer	0	0	0	1	1
Syncope	0	1	0	0	1
Diabetic Neuropathy	0	0	1	0	1
Hypersomnia	1	0	0	0	1
Loss Of Libido	0	1	0	0	1
Premenstrual Syndrome	0	0	0	1	1
Chest Injury	0	1	0	0	1
Rash Maculo-Papular	0	0	0	1	1
Allergic Cough	0	0	1	0	1
Scabies	1	0	0	0	1
Dyspepsia	0	1	0	0	1
Stye	0	0	0	1	1
Metrorrhagia	0	0	0	1	1
Helminthic Infection	0	0	0	1	1
Muscular Weakness	1	0	0	0	1
Urethral Discharge Syndrome	0	1	0	0	1
Dysuria	0	1	0	0	1
Vaccination Site Reaction	1	0	0	0	1
Eczema	0	1	0	0	1
Cystitis	0	1	0	0	1
Eye Infection	1	0	0	0	1
Gingival Ulceration	0	0	1	0	1

Neck Pain	0	0	1	0	1
Glossitis	0	1	0	0	1
Neuropathy	1	0	0	0	1
Rhinitis	1	0	0	0	1
Cold	0	1	0	0	1
Haematoma	0	1	0	0	1
Oedema	0	1	0	0	1
Sciatica	1	0	0	0	1
Oesophageal Candidiasis	0	1	0	0	1
Hallucination	1	0	0	0	1
Asthenia	1	0	0	0	1
Swelling Face	0	0	0	1	1
Orchitis	0	0	0	1	1
Bacterial Vaginosis	0	1	0	0	1
Otalgia	0	0	1	0	1
Hyperphagia	0	0	1	0	1
Otitis Media Acute	0	0	0	1	1
Dermatitis	1	0	0	0	1
Palpitations	0	1	0	0	1
Urethritis	0	0	1	0	1
Conjunctivitis	0	0	1	0	1
Urticaria	0	0	0	1	1
Atopy	0	0	0	1	1
Dermatitis Allergic	0	0	0	1	1
Angina Pectoris	0	1	0	0	1
Libido Decreased	0	0	1	0	1
Abdominal Distension	1	0	0	0	1
Ligament Sprain	0	0	0	1	1

Table S10. Serious adverse events

Screening ID	Age (years)	Preferred term	System organ class	Outcome	Relatedness	Study group
K0017	60	Sepsis	Infections and infestations	Recovered without sequelae	No	1000 IU
K0107	53	Cerebral Haemorrhage	Nervous system disorders	Fatal/died	No	1000 IU
K0201	28	Accidental Death	General disorders and administration site	Fatal/died	No	Standard
M0019	58	Sinusitis	Infections and infestations	Recovered without sequelae	No	250 IU
M0030	59	Myocardial Infarction	Cardiac disorders	Fatal/died	No	Standard
M0075	45	Malaria	Infections and infestations	Recovered without sequelae	No	1000 IU
M0118	32	Malaria	Infections and infestations	Recovered without sequelae	No	Standard
M0145	47	Umbilical Hernia	Gastrointestinal disorders	Recovered without sequelae	No	1000 IU
M0160	53	Cerebrovascular Accident	Nervous system disorders	Recovered with sequelae	No	Standard
M0195	53	Hypoglycaemia	Metabolism and nutrition disorders	Fatal/died	No	250 IU
M0203	51	Anaemia	Blood and lymphatic system disorders	Recovered without sequelae	No	1000 IU

References

1. Donken R, de Melker HE, Rots NY, Berbers G, Knol MJ. Comparing vaccines: a systematic review of the use of the non-inferiority margin in vaccine trials. Vaccine 2015;33(12):1426-32.
2. Food and Drug Administration. Non-Inferiority Clinical Trials to Establish Effectiveness: Guidance for Industry. US Department of Health and Human Services, 2016
3. Wu JT, Peak CM, Leung GM, Lipsitch M. Fractional dosing of yellow fever vaccine to extend supply: a modelling study. Lancet 2016;388(10062):2904-2911.
4. Agresti A, Coull BA. Approximate is Better than "Exact" for Interval Estimation of Binomial Proportions. The American Statistician 1998;52(2):119-126.
5. Hirji KF, Mehta CR, Patel NR. Computing Distributions for Exact Logistic Regression. Journal of the American Statistical Association 1987;82(400):1110-1117.
6. Schulz KF, Grimes DA. Sample size slippages in randomised trials: exclusions and the lost and wayward. Lancet 2002;359(9308):781-5.
7. Little RJA, Rubin DB. Statistical Analysis with Missing Data. 3rd ed. 2019.
8. Monath TP. Yellow fever: an update. Lancet Infect Dis 2001;1(1):11-20.
9. WHO. Situation Report: Yellow Fever, 28 October 2016. World Health Organization (http://appswhoint/iris/bitstream/10665/250661/1/yellowfeversitrep28Oct16-engpdf?ua=1)
10. WHO. Yellow fever – African Region (AFRO). 20 March 2024 (https://www.who.int/emergencies/disease-outbreak-news/item/2024-DON510).
11. Garske T, Van Kerkhove MD, Yactayo S, et al. Yellow Fever in Africa: estimating the burden of disease and impact of mass vaccination from outbreak and serological data. PLoS Med 2014;11(5):e1001638.
