# Supplementary material for: Low-Dose Yellow Fever Vaccine in Adults in Africa
Source: N Engl J Med. Author manuscript; Available in PMC 2025 Mar 9. (PMC7617464; doi:10.1056/NEJMoa2407293)
Supplement: Protocol [file EMS203456-supplement-Protocol.pdf]

Protocol

Protocol for: Kimathi D, Juan-Giner A, Bob NS, et al. Low-dose yellow fever vaccine in adults in Africa. N Engl J Med 2025;392:788-97. DOI: 10.1056/NEJMoa2407293

This trial protocol has been provided by the authors to give readers additional information about the work.

Low-dose vaccination for yellow fever in adults

Supplementary Appendix

Contents

1. Original Protocol Version 1.0 23 October 2018……………………….Page 2
2. Final Protocol Version 1.6 05 January 2023………………………….Page 43
3. Protocol summary of changes…………………………………………Page 88
4. Statistical Analysis Plan version 1.0 24 March 2021…………………Page 90

   **There were no changes to the Statistical Analysis Plan*

NON- INFERIORITY FRACTIONAL-DOSES TRIAL FOR YELLOW FEVER VACCINE

1. GENERAL INFORMATION

**Protocol Number:**	
**Trial Registration Number:**	
**Investigational Product(s):**	Yellow Fever Vaccine
**Funder:**	European & Developing Countries Clinical Trials Partnership (EDCTP)
**Tel:**	+31 70 344 0880
**Email:**	info@edctp.org
**Sponsor:**	University of Oxford, UK
**Tel:**	Tel: +254(0)709983549
**Email:**	pbejon@kemri-wellcome.org
**Drug/Product Manufacturer:**	Institut Pasteur de Dakar, Sénégal

Confidentiality Statement

The information contained herein is privileged or confidential and may not be disclosed unless such disclosure is required by applicable laws or regulations. In any event, persons to whom the information is disclosed must be informed that the information is privileged or confidential and may not be further disclosed by them. These restrictions on disclosure will apply equally to all future information supplied to you, which is indicated as privileged or confidential. This confidentiality statement also applies to data generated during the course of the study.

PRINCIPAL INVESTIGATOR'S APPROVAL OF THE PROTOCOL

The undersigned acknowledge possession of and have read the protocol "Non- Inferiority Fractional-Doses Trial for Yellow Fever Vaccine" on the 23rd October 2018. Having fully considered all the information available, the undersigned consider that it is ethically justifiable to give fractional does of Yellow Fever Vaccines to selected participants according to the agreed protocol. I understand that all information concerning fractional doses supplied to me in connection with this study is confidential information. This includes the Clinical Trial Protocol, Case Report Forms and any other preclinical and clinical data provided. I understand that no data are to be made public or published without prior knowledge and written approval by the University of Oxford.

By my signature below, I hereby attest that I have read, understood and agreed to abide by all the conditions, instructions and restrictions contained in this Protocol and in accordance with the most recent Declaration of Helsinki, Good Clinical Practice and all applicable regulatory requirements. I acknowledge that the Sponsor of the study has the right to discontinue the study at any time.

Principal Investigator: **Signature:** **Date:**

Co-Principal Investigator: **Signature:** **Date:**

GLOSSARY OF TERMS AND ABBREVIATIONS:

AE	Adverse Event
CI	Confidence Interval
CRF	Case Report Form
DRC	Democratic Republic of Congo
DSMB	Data and Safety Monitoring Board
EPI	Expanded Programme on Immunization
GCP	Good Clinical Practice
GDPR	General Data Protection Regulation
GMT	Geometric Mean $PRNT_{50}$ Titre
GMFI	Geometric Mean Fold Increase
HIV	Human Immunodeficiency Virus
IB	Investigator Brochure
ICF	Informed Consent Form
ICH	International Conference on Harmonization
ID	Intradermal
IgG	Immunoglobulin G
IP	Institut Pasteur
IRB	Institutional Review Board
ISF	Investigator site file
ITT	Intention to treat
IU	International Units
LOQ	Limit of Quantification
MLD50	Mouse Lethal Dose 50
MSF	Medecins Sans Frontieres, Doctors without borders
NRA	National regulatory authority
OXTREC	Oxford Tropical Research Ethics Committee
PFU	Plaque Forming Units
PI	Principal Investigator
PIS	Patient information sheet
PP	Per protocol

PPB	Pharmacy and Poisons Board
PRNT	Plaque-Reduction Neutralization Test
RAP	Report and Analysis Plan
RKI	Robert Koch Institute
SAE	Serious Adverse Event
SAGE	Strategic Advisory Group of Experts on Immunization
SAP	Statistical Analysis Plan
SC	Subcutaneous
SSL	Secure Sockets Layer
SOP	Standard operating procedure
SUSAR	Suspected unexpected serious adverse reaction
UNICEF	United Nations International Children's Emergency Fund
UVRI	Uganda Virus Research Institute
UNHRO	Uganda National Health Research Organization
WHO	World Health Organization
YEL-AND	Vaccine-associated neurological diseases
YEL-AVD	Vaccine-associated viscerotropic disease
YF	Yellow Fever

Table of Contents

1. GENERAL INFORMATION ... 1
2. LAY SUMMARY ... 6
3. LIST OF INVESTIGATORS ... 7
4. ABSTRACT ... 8
5. INTRODUCTION ... 9
6. NAME AND DESCRIPTION OF THE INVESTIGATIONAL PRODUCT ... 13
7. TRIAL OBJECTIVES AND PURPOSE ... 13
8. TRIAL DESIGN ... 14
9. SELECTION AND WITHDRAWAL OF STUDY PARTICIPANTS ... 21
10. TREATMENT OF STUDY PARTICIPANTS ... 22
11. LAB PROCEDURES ... 24
12. ASSESSMENT OF SAFETY ... 25
13. STATISTICS ... 28
14. DIRECT ACCESS TO SOURCE DATA/DOCUMENTS ... 31
15. QUALITY CONTROL AND QUALITY ASSURANCE ... 31
16. INTELLECTUAL PROPERTY ... 32
17. TIME FRAME/DURATION OF THE TRIAL ... 32
18. ETHICS ... 33
19. ARCHIVING AND RECORD RETENTION ... 35
20. FINANCING AND INSURANCE ... 37
21. TRIAL MANAGEMENT ... 38
22. REPORTING, DISSEMINATION AND NOTIFICATION OF RESULTS ... 38
23. APPENDICES ... 39

2. LAY SUMMARY

Formal Title: Non- inferiority fractional-doses trial for yellow fever vaccine

Lay Title: A trial to determine the safety and immune response elicited by the normal full dose of yellow fever vaccine in comparison to reduced doses of the vaccine in Kenyan and Ugandan adults and children

What is the problem/background?
Yellow fever (YF) is a disease caused by a mosquito-borne flavivirus that is endemic in sub-Saharan Africa and tropical South America. YF virus infection can cause mild or severe illness, leading to jaundice, kidney failure, bleeding and death. The YF vaccine is shown to be very effective for disease control, including prevention of YF outbreaks. However insufficient vaccine is produced for routine use, and whilst a YF vaccine stockpile is reserved for outbreak control, this is frequently depleted. Measures to increase the global supply of YF vaccine are urgently needed.

What questions are we trying to answer?
The World Health Organization (WHO) has recommended consideration of using fractions of standard YF vaccine dose to be able to vaccinate more individuals with a given quantity of vaccine. In this study, we will assess whether the immune response and adverse events occurring after vaccination of adults and children with the standard full dose of YF vaccine are comparable to those observed after vaccination with one of three lower doses of vaccine. Further, to support the implementation of a future YF control strategy using fractional doses of vaccine, we will evaluate the views and perceptions of different stakeholders involved in national or international vaccine policy regarding the use of fractional YF vaccine doses.

Where is the study taking place, how many people does it involve and how are they selected?
The vaccination study will take place in Kilifi, Kenya and Mbarara, Uganda among healthy adults and children who have previously not had the YF vaccine and/or YF infection. In total, 480 adults (240 at each site) and 420 children aged 9 months to 5 years (210 at each site) will be included. To evaluate views and perceptions on the use of fractional YF vaccine doses we will approach and conduct discussions and interviews with various stakeholders in national vaccine policy in Kenya, Uganda, Senegal and other international institutions (see figure 1 on protocol).

What does the study involve for those who are in it?
Participants who have previously not had the YF vaccine and/or YF infection will be screened for any significant health problems. Those found eligible to participate will then be randomized to receive a single dose of the standard full dose of YF vaccine (group 1) or any of the three lower doses (groups 2 to 4). Participants will then need to attend follow up to have blood taken for tests to measure immune response to vaccination, the level YF vaccine in the body, and to be asked about any side effects. Vaccine policy stakeholders included in the study will be requested to participate in interviews and discussions at their convenience.

What are the benefits and risks/costs of the study for those involved?
The risks relate to the possibility of developing an allergic or other reaction upon administration of the YF vaccine. There are no immediate health benefits to individuals participating in this study other than information about their health. If there was a YF outbreak in the future, participants getting the full YF vaccine dose would be expected to be protected and there is a chance that participants who receive the lower dose would be protected against infection. We are not able to issue YF vaccination certificates to participants at the point of vaccination. If the participant travels to endemic areas and needs a YF vaccination certificate for proof of vaccination, they will need to be revaccinated at an authorized health facility in Kenya or Uganda, respectively. However, on completion of the study we will offer to pay for the cost of re-vaccination at an authorized public health facility for any study participant who would like to receive a YF vaccination certificate. No safety concerns are reported with multiple doses of the vaccine.
The vaccine policy stakeholders participating in the study will not be at any risk associated with the vaccine trial. Any costs they incur during the process of the study will be reimbursed as out of pocket expenses.

How will the study benefit society?
If any of the lower YF vaccine doses safely elicits immune response that are comparable to the full vaccine dose, then in effect this finding will substantially increase the number of doses that can be given based on the world's currently available vaccine stock, and thereby enhance our ability to prevent and control YF outbreaks. The results from the interviews and discussions with vaccine policy stakeholders will support the development of a strategy to implement the use of lower YF vaccine doses for disease control.

When does the study start and finish?
The study will start upon receipt of ethical approval and will be completed in 40 months.

3. LIST OF INVESTIGATORS

KEMRI CGMRC Investigators: George Warimwe (PI), Derick Kimathi (Co-PI), Philip Bejon, Sassy Molyneux, Mainga Hamaluba, Henry Karanja, John Gitonga, Marianne Munene, Jackline Wafula

Epicentre, Mbarara Investigators: Patrick Kazooba, Juliet Mwanga, Dan Nyehangane

<u>Collaborators</u>

IP Dakar, Senegal: Amadou Sall, Antoine Diatta

UNHRO, Uganda: Julius Lutwama, John Kayiwa, Pontiano Kaleebu

Epicentre: Rebecca Grais, Aitana Juan-Giner

4. ABSTRACT

In July 2016, the demand for yellow fever (YF) vaccines in response to the large urban outbreaks occurring concurrently and the risk of further spread through Africa and to Asia was larger than the available global supply. In this situation, the World Health Organization (WHO) developed recommendations for the use of fractional doses of YF vaccine as a dose-sparing strategy. These recommendations were based on data from a limited number of clinical trials, none of which had been conducted in Africa. Additional studies were initiated to assess the applicability of fractional doses to all four WHO-prequalified YF vaccines with respect to vaccine immunogenicity in adults and children in Africa, including HIV positive adults. One such study, comparing full standard dose to 1/5th of standard dose of all four WHO-prequalified YF vaccines in adults (Clinicaltrials.gov number: NCT02991495), is currently ongoing at KEMRI CGMRC (see SERU protocol 3452) and Epicentre, Mbarara (UNCST HS 2237) and is designed to answer questions on the use of current stock of YF vaccines with a potency as close as possible to each manufacturers' minimum release. Data from this pragmatic trial will inform a WHO recommendation on using 1/5th of the current standard dose of vaccine for outbreak control. However, since many vials will contain excess YF vaccine such that 1/5th of a vial is likely to be substantially above the current minimum potency requirements, these data may not be scientifically explanatory regarding the minimum dose required for preventive use.

Here, in this new complementary study, we aim to determine the lowest YF vaccine dose that is non-inferior to the current standard full dose among populations in sub-Saharan Africa. The study will be conducted in Kenya (KEMRI CGMRC, Kilifi) and Uganda (Epicentre, Mbarara) with trial participants recruited at both sites, using vaccine from one WHO-prequalified manufacturer (Institut Pasteur de Dakar, Senegal). Our primary aim is to compare the immunogenicity of full standard dose of vaccine with three lower doses in adults, using seroconversion at 28 days post-vaccination, measured by plaque reduction neutralization assay ($PRNT_{50}$), as the endpoint. Adult participants (n=480) will be randomized for vaccination with full standard dose or with approximately 1000, 500 or 250 IU of vaccine (i.e. 4 arms, 1:1:1:1 allocation ratio). Safety and immunogenicity results will then be reviewed by the study DSMB, and the lowest non-inferior dose selected for assessment in a sub-study in children (n=420) in comparison to full standard dose (i.e. 2 arms, 1:1 allocation ratio). Secondary objectives will include immunogenicity at 10 days, 1 year and 2 years post-vaccination, assessment of post-vaccination viremia, assessment of the influence of cross-reactive antibodies to other flaviviruses on vaccine immunogenicity, and occurrence of serious adverse events (SAE). In addition, we will assess the range of views and perceptions of key stakeholders in vaccine policy and implementation on fractional vaccine dose usage during YF epidemics and routine use. We expect these data to inform the implementation of policies relating to low-dose vaccine usage for the control of YF and other diseases (e.g. pneumococcal vaccines) where such strategies are in consideration.

5. INTRODUCTION

5.1. Background Information

Yellow fever (YF) is a disease caused by a mosquito-borne flavivirus that is endemic in sub-Saharan Africa and tropical South America [1]. Ninety percent of YF cases are in Africa. In these settings, YF virus is transmitted by different mosquito genera in three recognized transmission cycles [2]. A sylvatic cycle involves transmission between forest-dwelling mosquitoes (*Haemagogus spp*) and non-human primate reservoirs, with sporadic incidental transmission to humans (e.g. forest workers). An intermediate cycle, occurring only in Africa, involves mosquito transmission between non-human primates and humans, or human-to-human transmission among humans living or working close to forested areas. An urban cycle involves transmission between humans and urban mosquito vectors, primarily *Aedes aegypti*, and occurs when a viraemic person, infected in the sylvatic or intermediate cycle, introduces YF virus to areas with a large non-immune population and *A. aegypti* vectors resulting in disease outbreaks [2].

Infection with YF virus is characterised by a wide range of manifestations, ranging from subclinical infection with mild and non-specific symptoms, to severe, life-threatening illness with jaundice, renal failure and haemorrhage [3]. The first symptoms appear 3-6 days after an infected mosquito bite and present with an abrupt onset of fever, muscle pain, headache, shivering, loss of appetite, and nausea or vomiting. These symptoms are commonly accompanied by congestion of the conjunctivae and face, and bradycardia despite fever. During the symptomatic period, lasting 3-6 days, the patient is usually viraemic, but this is usually followed by a remission period. Approximately 15% of infected individuals then enter a toxic phase with renewed fever, relative bradycardia, nausea, vomiting, epigastric pain, jaundice, oliguria, and a haemorrhagic diathesis. The symptoms and severity of the disease reflect dysfunction of multiple organ systems, including the liver, kidneys, and cardiovascular system [3]. Whilst there is no specific antiviral treatment for YF, a highly effective vaccine that provides lifelong protective immunity is available [4]. For instance, a recent modelling study using YF case reports between 1987 and 2011 estimated that there were 1.3 million YF infections in Africa in 2013 (95% CI 850,000 – 1.8 million), of which 180,000 were severe (95% CI 51,000 – 380,000) and 78,000 were fatal (95% CI 19,000 – 180,000) [5]. Preventive mass vaccination campaigns were estimated to have averted 450,000 cases (95% CI 340,000 – 560,000) and 28,000 deaths (95% CI 7,200 – 62,000), highlighting the importance of vaccination as disease control tool [5]. However, despite the availability of an effective vaccine, incomplete vaccine coverage, below the WHO-recommended 80% coverage [6], leaves some settings prone to outbreaks as has recently occurred in Angola, Democratic Republic of Congo (DRC) and Brazil [7, 8].

5.2. Yellow Fever vaccines

A highly effective vaccine is available for use against YF in adults and children aged ≥9 months [4]. The vaccine is a freeze-dried preparation of live attenuated YF virus strain 17D, which was developed in 1937 and is produced by four WHO-prequalified manufacturers (Table 1) [9]. A single dose of YF vaccine is considered sufficient to confer life-long protective immunity against all seven known genotypes of wild-type YF virus [4]. Protective levels of YF virus neutralizing antibodies are developed in 80-100% vaccine recipients within 10 days after vaccination, and in 99% within a

month [4]. Seroconversion rates appear to be similar regardless of vaccine sub-strain and manufacturer, though vaccine immunogenicity appears to be somewhat lower in children [4]. Two sub-strains of the 17D vaccine are currently used for vaccine production, namely 17D-204 and 17DD. A distinct sub-strain of 17D-204 (i.e. 17D-213) is also in use (Table 1).

The YF vaccine is prepared using various seed strains that were ultimately derived from the 17D strain of YF virus cultured in chicken embryonated eggs [9]. The seed virus is inoculated into 7 to 9 day-old embryonated eggs and after 3-4 days of incubation, infected embryos are aseptically harvested, homogenized and clarified by centrifugation to produce bulk vaccine. Following addition of stabilizers, the diluted bulk vaccine is filled into vials and freeze-dried [9]. As per WHO recommendations, the final vaccine vials/ampoules should contain a minimum of 1000 IU per dose [10]. However, the dose in the final vaccine vials/ampoules usually exceeds the minimum specification substantially to account for potential potency losses during manufacture and the three years shelf-life [10, 11]. The YF vaccine manufacture process is laborious and current capacity to produce increased stock in response to outbreaks is limited [9].

Table 1: Yellow Fever vaccines pre-qualified by WHO (August 2016)

Manufacturer	Commercial Name	Pharmaceutical Form	Sub-strain	Presentation	No. of Doses
Sanofi Pasteur SA	STAMARIL	Lyophilised active component to be reconstituted with excipient diluent before use	17D-204	Vial	10
Bio-Manguinhos/ Fiocruz	Yellow Fever	Lyophilised active component to be reconstituted with excipient diluent before use	17DD	Vial + Ampoule	10
					5
				Two vial set (active + excipient)	50
Institut Pasteur de Dakar	Stabilized Yellow Fever Vaccine	Lyophilised active component to be reconstituted with excipient diluent before use	17D-204	Vial	5
					20
					10
Federal State Unitary Enterprise of Chumakov Institut of Poliomyelitis and Viral Encephalitides	-	Lyophilised active component to be reconstituted with excipient diluent before use	17D-213	Ampoule	2
					5
					10

In 2015, UNICEF estimated the total country forecasts for the period from 2015 to 2017 to be 64 million doses per year, exceeding the availability of vaccines by 42%. UNICEF stated that their annual procurement is able to cover routine immunization requirements, emergency stockpiles and some limited additional campaigns. In 2000, a global shortage of YF vaccine occurred and this led to

the development of the International Coordination Group (ICG) on vaccine provision for YF. The aim of the ICG is to manage and coordinate the provision of emergency vaccine supplies during outbreaks and ensure the best allocation of limited resources(18). In 2000, a stockpile of 2 million doses was reserved for outbreak response. This was increased from 2 to 6 million doses in 2014. However, the stockpile was depleted twice in 2016.

The relatively long-time period required for vaccine production, together with poor epidemiological surveillance and reporting in at-risk countries, makes vaccine need forecasting very difficult. A review conducted by PATH identified a number of factors that limit the production of YF vaccines [12]. The first is related to the small number of YF vaccine manufactures owing, in part, to the absence of a stable demand. Competition for production capacity with other vaccines that are economically more attractive is also an issue. Other limiting factors are related to the production process and include the limited number of Specific Pathogen-Free egg suppliers, gradual depletion of existing seed stocks and a lyophilisation process that can take several days per cycle. There are limited options for developing technological advances that will solve these issues in the near future. The review by PATH concluded that, to address the current insufficient supply of YF vaccine, fractional dosing could be a short to medium term option provided that clinical evidence for non-inferiority, safety and dosages is generated [12].

5.3. Use of fractional doses as a dose-sparing strategy

In July 2016, the demand for YF vaccine in response to the large urban outbreaks occurring concurrently in different parts of Africa and the risk of further spread throughout the continent and to Asia, led WHO to develop recommendations for the use of fractional-dose of YF vaccine as a dose-sparing strategy. Although fractional dosing has recently been used in vaccination campaigns in Kinshasa [13], WHO recommendations were based on a limited number of clinical studies and important data gaps remain. These include the applicability of fractional dosing to all WHO-prequalified vaccines, the persistence of neutralizing antibodies and the performance of fractional doses in young children and in populations in Africa, including those with HIV [11].

Between July 2017 and March 2018, Brazil has had YF outbreaks with reports of over 720 confirmed human cases including over 230 deaths by February 2018. Fractional doses of YF vaccine were used in selected municipalities to respond to the outbreak following a recommendation by WHO and PAHO (http://www.who.int/csr/don/09-march-2018-yellow-fever-brazil/en/).

In 1988, a study showed that the use of 200 plaque-forming units (PFU) induced seroconversion in 100% of participants [14]. However, the vaccine was based on older formulations and we cannot be certain how PFU in that study relate to IU [10]. Two more recent trials have examined the immunogenicity and safety of low doses of YF vaccine. One study examined intradermal (ID) administration of 1/5th of the conventional subcutaneous (SC) dose and showed that this was sufficient to achieve seroconversion in 77 adult participants [15]. In a second, more recent study, YF vaccine was administered by SC route in 900 healthy adult males in de-escalating doses. The study concluded that a dose of ≥587IU was as immunogenic as a 50-fold higher dose (27,476IU/dose), and that these immune responses were sustained over an 8-year period [16, 17].

However, fractional vaccine dosing is compounded by the uncertainty surrounding minimum dose requirements. The current WHO recommendation is that YF vaccine potency should contain not less

than 1,000 IU per dose [10]. A thermostability test is undertaken to demonstrate consistency of production. This should show that the geometric mean infectious titre, following incubation of the final containers at 37°C for 2 weeks, is at least 1,000 IU per dose and not have decreased by more than 1.0 log_{10} IU [10]. The minimum vaccine dose for potency was established in the 1930s and 40s based on experience with lots that varied in titre. The original work was based on mouse median lethal doses (MLD) using intracranial injection. MLD was replaced by assays for PFU, and comparisons between MLD and PFU were used to translate dose requirements. These comparisons varied by manufacturer, and the WHO introduced an international standard to allow doses to be expressed in IU [10]. Therefore, despite the apparent high vaccine effectiveness observed since thresholds were determined in the 1940s, there is uncertainty regarding the precision with which the minimum dose requirements are known.

5.4. Safety considerations for fractional doses

Reactions to YF vaccine are generally mild and include headache, myalgia, malaise and asthenia in around 10-30% of participants during the first few days after vaccination [3, 18]. Serious reactions are rare and include hypersensitivity reactions to egg protein, or to the gelatine used by some manufacturers; vaccine-associated neurological diseases (YEL-AND) and vaccine-associated viscerotropic disease (YEL-AVD), which can vary from multi-organ system failure with limited evidence of hepatitis to a fulminant hepatitis resembling wild-type YF [3, 18]. YEL-AVD is rare (1 in 100,000 vaccinees), occurs within 10 days of a first dose of YF vaccine, and is characterized by severe multi-organ failure. Known risk factors include a history of thymus disease (e.g. thymoma or thymectomy) and age ≥60 years. It has been suggested that the association between serious adverse reactions and primary vaccination may be due to the viremia that primary vaccinees experience following vaccination [18]. The viremia is short-lived, with 17D virus and viral RNA being detectable in the first week post-vaccination, and disappearing with the development of neutralizing antibodies following vaccination [18]. Monkey studies have suggested an inverse relationship between YF vaccine dose and the magnitude and duration of vaccine viremia [18, 19]. However, whilst more studies are needed to assess the impact of low vaccine doses on viremia, a previous fractional dosing trial in adults in Brazil found no association between viremia levels and vaccine dose [16].

5.5. Justification

The ongoing trial (SERU protocol number 3452, MUST-REC reference number 04/01-17) is designed to answer questions on the use of vaccines from current stock with doses as close as possible to the minimum release of each WHO-prequalified YF vaccine manufacturer (Table 1). This trial was prompted by the 2016 DRC outbreak and compares the immunogenicity of standard full dose of YF vaccine to the immunogenicity of $1/5^{th}$ of a current dose using vaccine from all four WHO prequalified manufacturers. Data from the trial will inform WHO recommendations on the use of $1/5^{th}$ of the standard dose of vaccine to produce an immune response. However, standard doses contain excess YF vaccine such that $1/5^{th}$ of a vial is likely to be substantially above the current minimum requirements [11]. Thus, these data may not be scientifically explanatory regarding the

minimum dose required for further practice. This study aims to influence the WHO recommendation for the minimum release dose specifications based on current data. This will be done through regulatory engagements and different pathways to impact as advised by experts within the study consortium.

This study therefore aims to determine the lowest dose (in IU/dose) that is non-inferior to the standard full dose among populations in sub-Saharan Africa. The data generated in this study will provide further confidence in, and inform recommendations for, the use of fractional doses of YF vaccine during epidemics, and potentially, for routine vaccination. In addition, we will assess the range of views and perceptions of key stakeholders in vaccine policy and implementation on fractional vaccine dose usage during YF epidemics and for routine use. We expect these data to inform the implementation of policies relating to low-dose vaccine usage for the control of YF and other diseases (e.g. pneumococcal vaccines) where such strategies are in consideration.

6. NAME AND DESCRIPTION OF THE INVESTIGATIONAL PRODUCT

We will use YF vaccine produced by Institut Pasteur de Dakar, Senegal. IP Dakar is a member of the study consortium and will supply the investigational product. The product will be a freeze-dried preparation of live attenuated YF virus, sub-strain 17D-204. One vial format will be provided. It will contain standard full dose of vaccine for administration in 0.5ml per dose. The standard dose here is defined as the dose produced by the vaccine manufacturer above the minimum release of 1000 IU/dose. A second vial will be prepared from a standard vial to contain vaccine diluted to approximately 1000 IU/dose for administration in 0.5ml per dose. Fractional volumes of this second vial will be administered as per manufacturer's instructions to achieve the 500 IU and 250 IU doses. A single vaccine batch will be used in this study.

7. TRIAL OBJECTIVES AND PURPOSE

7.1. Null hypothesis

Vaccine trial: The rate of seroconversion among vaccinees receiving the 1000, 500 or 250 IU dose of vaccine is lower than that in vaccinees receiving the full standard dose by >5%, as measured by plaque reduction neutralization antibody test ($PRNT_{50}$) 28 days post-vaccination.

Assessment of key vaccine policy stakeholder perceptions: This component of the study will be descriptive.

7.2. Primary objective

To determine the lowest dose (1000, 500 and 250 IU/dose) of YF vaccine that is non-inferior to the full standard dose as measured by seroconversion using the $PRNT_{50}$ assay at 28 days post-vaccination in an adult population.

7.3. Secondary objective(s)

- To describe the geometric mean $PRNT_{50}$ titre (GMT) at 10 days, 28 days, 1 year and at 2 years post-vaccination of the different doses of the YF vaccine.
- To describe the change in $PRNT_{50}$ titre (i.e. the geometric mean fold increase (GMFI) as a continuous variable) between baseline and day 28 after vaccination with the different doses of the YF vaccine.
- To map out key stakeholders' priorities and perceptions regarding a change in policy towards the use of fractional doses of YF vaccine and their potential influence on policy process.
- To assess interference of antibodies to other flaviviruses with YF vaccine immunogenicity.
- To assess post-vaccination control of viremia by vaccine dose.
- To assess the occurrence of adverse events (AE) 28 days after vaccination and serious adverse events throughout the duration of the study.

Once results for the main outcome are obtained, data will be reviewed by the study Data and Safety Monitoring Board (DSMB). The DSMB will then decide if the study should proceed to the second phase where the lowest non-inferior dose, as measured at day 28 in the adult study, will be selected for assessment in children aged 9 months to 5 years. The aim of this sub-study will be to assess the non-inferiority in seroconversion of the fractional dose compared to the full standard dose as measured by $PRNT_{50}$ at 28 days post-vaccination. The procedures described in this protocol include both the study on the adult population and the sub-study in children.

8. TRIAL DESIGN

8.1. Overall Study Design and Plan Description

This will be a parallel group, randomized, controlled, blinded, non-inferiority trial of four different doses of YF vaccine. The study will be conducted at the KEMRI CGMRC in Kilifi, Kenya and at Epicentre in Mbarara, Uganda. Both these sites are already working together in an ongoing study (Clinicaltrials.gov number: NCT02991495).

Adult participants (n=480) will be randomized for vaccination with full standard dose or with 1000, 500 or 250 IU (i.e. 4 arms) with a 1:1:1:1 allocation ratio. Results for the safety and primary outcome of the adult study will then be reviewed by the DSMB, and the lowest non-inferior dose in the adult study selected for assessment in children aged 9 months to 5 years (n=420) in comparison to full standard dose (i.e. 2 arms) with a 1:1 allocation ratio. The determination of the non-inferior dose to use in children will be made by the sponsor in discussion with the study DSMB, vaccine manufacturer and relevant stakeholders, and the final decision communicated to the various regulatory authorities as a notification (i.e. SERU, OXTREC and PPB for the Kilifi site, MUST-REC, UNCST and NDA for the Mbarara site).

Adult vaccinees will be followed up for 2 years, and children for 1 year. There will be no gradual age de-escalation on the basis that there are few safety concerns with the full dose of YF vaccines,

having been used in millions of children worldwide. The study procedures will be similar across both sites and these are summarized in Table 2.

Table 2: Study schedule

Procedure	Screening Day -30*	Day 0	Day 2, 3, 4, 5, 6, 7**	Day 10 (+/- 1 day)	Day 28 (+/- 3 days)	Day 365 (+/-14 days)	Day 730 (+/- 28 days)
Informed Consent	X						
HIV Antibody test	X						
Pregnancy test	X	X			X		
Demography	X	X					
Vital signs	X	X		X	X	X	X
History and Physical exam	X			X	X	X	X
Randomization		X					
Vaccination		X					
Blood sample (Adults, Children)		X (10ml, 6ml)	X (4ml, 4ml)	X (10ml, 6ml)	X (10ml, 6ml)	X (10ml, 6ml)	X (10ml, 0ml)
Cumulative blood volume (Adults, Children)		10ml, 6ml	14ml, 10ml	24ml, 16ml	34ml, 22ml	44ml, 28ml	54ml, 28ml
Adverse events and serious Adverse Event			X	X	X	X	X

*Before vaccination. Screening can occur between 0 and 30 days before vaccination. Screening and vaccination can occur on the same day, but participants that are not recruited within 30 days will be re-screened.

**All participants will be randomized to provide one 4ml blood sample at one of 6 time points (days 2, 3, 4, 5, 6, or 7).

8.2.1. Recruitment

Using existing community engagement strategies developed and successfully implemented for the ongoing trial (Clinicaltrials.gov number: NCT02991495), potential participants will be sensitized, willing volunteers screened, enrolled and vaccinated. During recruitment and consent (see below) we will make it clear that participation will not reliably result in immunity to YF. If participants are subsequently involved in an outbreak or need to travel, unless they have been informed that they received a vaccination that induces immunity in our trial feedback, they should assume that re-vaccination with the full dose YF vaccine is required to be assured of immunity. It should also be noted that the study will not provide certificates of vaccination and these may be required for travel to certain destinations. However, on completion of the study we will offer to pay for the cost of re-vaccination at an authorized public health facility for any study participant who would like to receive a YF vaccination certificate. No safety concerns are reported with multiple doses of the vaccine.

8.2.2. Informed Consent and Screening

Before any study specific procedures are undertaken, a member of the study team (clinician, nurse, counselor or field worker), specially trained for the informed consent process, will go through the consent process and participants will be asked to give their individual consent to participate on an

informed consent form (ICF) developed specifically for the study. The informed consent will be conducted at the study sites, in private rooms.

The informed consent process will ensure that potential participants have an understanding of the potential risks and benefits of participating in the study, the study procedures (including maintaining confidentiality and anonymity) and study assessment schedule, the use of the blood sample and their right to refuse and/or withdraw from the study at any point without affecting any of the other health services or care they receive, and without having to disclose a reason for their refusal or withdrawal.

Participants will be required to read the full consent or receive a full oral explanation (for illiterate participants) in the language of their choice. Participants will be asked individually by a trained study team member if they understand all parts of the consent and will be given the opportunity to ask any questions and seek clarification. Consent will then be obtained. All informed consent documents will be translated into Kiswahili in Kilifi and Runyankore in Mbarara. For illiterate participants, a witness, selected by the potential participant and not related to the study team, will be requested to be present during the process.

For children (9 months to 5 years of age), consent to participate in the study will be requested from parents or guardians. At least one parent, or guardian, will provide written informed consent for her/his child to participate in the study. As with the adult participants, informed consent will be obtained before any studies procedures are undertaken. The child will be withdrawn from the study if the parent or guardian decides it is in the child’s best interest.

During the informed consent process, participants will be given the contact details of a designated study staff member and will be advised to contact the study team (by telephone or in person at the study site) if they have a health problem. Participants will be contacted by phone by a member of the study team to be reminded about the participation in the study and the follow-up visits. The study team will take contact details of each participant at the screening visit. A copy of the consent form and participant information sheet (PIS) will also be provided to the participant before concluding the screening visit.

This study aims to include healthy individuals who have no contraindications for receiving a YF vaccine. All screening procedures will be similar for all participants. Participants will be screened for eligibility by clinical examination, urine-based pregnancy test and blood tests for HIV serology. During the screening visit, a clinician will assess participants and check the inclusion and exclusion criteria and a rapid HIV test will be done according to Government of Kenya guidelines and the Ministry of Health, Uganda guidelines. This will include the implementation of pre-and post-test counseling as specified in national guidelines. Newly diagnosed HIV persons will be linked to comprehensive care according to the guidelines. Relevant demographic information at baseline will also be collected. Antibodies to YF virus at baseline will be used in analysis but will not be an exclusion criterion.

The inclusion criteria will include:

- Individuals aged ≥18 - <60 years of age.

  - For the sub-study, healthy children aged 9 months (minimum age recommended by WHO for YF vaccination) to 5 years whose parents/guardians are willing to consent for their participation into this study.

- Individuals who can provide informed consent to participate in the study
- HIV status
  - HIV negative on serological screening OR
  - HIV positive on serological testing, and no symptoms suggestive of current clinical immunosuppression and CD4 count>200 within the last 6 months.

The exclusion criteria include:

- Known contraindications to YF vaccination such as allergies to egg protein or any component of the vaccine (including gelatin, eggs, eggs products or chicken products), immunodeficiency, known thymus disorder, such as thymoma and myasthenia gravis
- Using corticosteroids or other immunosuppressive therapy
- Thymus disorder, such as thymoma and myasthenia gravis
- Acute febrile disease on the day of vaccination with temperature >37.5 degrees Celsius.
- Previous YF vaccination
- Previous YF infection as determined from history
- Pregnancy (as determined by a urine test on the proposed day of vaccination) and lactating women
- Planning to migrate out of the study areas before the end of the study follow-up
- Planning to travel to a country requiring YF vaccination certificate within the first year after vaccination.

The screening visit may occur a maximum of 30 days before vaccination. Clinical assessments will be repeated if more than 30 days elapse between screening and proposed enrolment. However, screening and vaccination may occur on the same day.

Participants of the qualitative study evaluating vaccine policy stakeholder views and perceptions will be approached for a request to participate in an interview and discussion, scheduled at their convenience. Data collection will only be initiated upon signing an Informed Consent Form.

8.2.3. Randomization / Enrollment

Participants meeting the inclusion and none of the exclusion criteria above will be enrolled in the study. Vaccination may occur on the day of screening, or deferred to a later day, depending on the timing of screening and vaccination days. Randomization will take place when a volunteer with confirmed eligibility criteria attends for vaccination and completes the enrollment pre-vaccination

assessment successfully. This will include an assessment for acute febrile disease, and a urine pregnancy test for female volunteers.

Children who will have received or scheduled to receive EPI vaccination will be vaccinated 4 weeks after or 4 weeks before the EPI vaccination to allow for AE assessment and avoid any immunological interference.

Each adult participant will be randomized to receive one of the four vaccine doses. Computer-generated randomization codes will be prepared in randomization booklets before recruitment starts by a person outside of the study. Participants will be allocated to one of the four treatment arms as per the computer-generated randomization schedule. To minimize vaccine wastage, randomization will be done in block sizes that match with clinic visit days. Allocations will be concealed until a member of the unblinded study team scratches the randomization booklet to reveal the participants' randomization arm. Participants will not be informed of the allocated vaccine dose. The unblinded team will observe aseptic techniques when drawing up vaccine from vials and the barrel of the syringe masked using tape to avoid observation of the volume. Participants will be told that this is to avoid them becoming aware of the vaccination dose before the end of the trial, as the vaccines will be drawn up into different sized syringes. The vaccine will be administered subcutaneously in the deltoid region of either arm and upper thigh for children <1 year, avoiding broken skin or injuries. It is recommended, but not required, that the injection be administered into the non-dominant arm. The unblinded team will not participate in further assessments or follow-up visits and will not reveal dose allocations to participants or other trial personnel. All other members of the study team will be blinded until data are locked.
Children enrolled in the sub-study will be randomized to vaccination with the full standard dose or the lowest non-inferior dose determined after review of the adult day 28 immunogenicity and safety data. Similar randomization procedures as the adult study will be used. The sponsor in discussion with vaccine manufacturer, the study DSMB and relevant stakeholders will make the determination of which lower vaccine dose to use in children and this decision communicated to the various regulatory authorities as a notification (i.e. SERU, OXTREC and PPB for the Kilifi site, MUST-REC, UNCST and NDA for the Mbarara site).

All participants will receive the same information regarding adverse events. Once the vaccine has been administered, the participant will remain under observation for at least thirty minutes to monitor for any immediate reactions. The necessary equipment will be in place to manage any hypersensitivity reactions. Any immediate local and systemic reactogenicity will be recorded and addressed before they leave the clinic. The participant will then be asked to return to the clinic for the next visit. Vaccine vials will be handled as per manufacturer's instructions, with cold storage verified by a temperature tracker. Vaccine vials that are opened and not used within 6 hours will be discarded.

8.2.4. Interim visits

At each scheduled follow-up visit, participants will have a blood sample taken (see Table 2). The participants will all be randomized to have one additional blood sample collected at either day 2, 3 4, 5, 6 or 7 for viremia assessment (Table 2). As the transmission of HIV and other blood borne pathogens can occur through contact with contaminated needles, blood and blood products,

appropriate blood and body fluid precautions will be employed by all personnel involved in drawing of blood, testing, and handling of all specimens for this study, using standard universal precautions. On visits up to day 28 post-vaccination, participants will be asked retrospectively about adverse events and serious adverse events occurring currently or since the previous visit and these will be documented in the CRF.

8.2.5. Unscheduled visits

Participants will be reminded to contact the study team if they experience symptoms of concern related to the expected vaccine reactions between scheduled study assessments. Any interventions required to treat a disease or condition in an enrolled participant will be allowed. Concomitant administration of other vaccines included in the EPI schedule is accepted. All concomitant interventions will be determined by asking the participant at the scheduled visits and recorded in the appropriate CRF pages.

The study team will provide medical care to participants during the study follow-up period for acute illnesses. The study teams will not become responsible for long-standing chronic conditions that were present before vaccination, or that are unrelated to vaccination. Medical care will be provided within the Kenyan and Ugandan Ministry of Health guidelines, respectively.

Data on adverse events and serious adverse events will be collected as described in later sections below. Female participants becoming pregnant during the follow-up period will be referred for ante-natal visits to health facilities of their choice and pregnancies will be followed-up and outcome recorded if the pregnancy is detected within 28 days of vaccination as described in section below.

8.2.6. End of treatment visit

To reduce loss to follow-up at the 12-month (adult and children studies) and 24-month visit (adult study only), the study team will contact participants by phone at around 6 months and 18 months after inclusion to remind them about their participation in the study and about the remaining follow-up visit. The 24-month visit will be the final study visit. A blood sample will be taken, and the participants' general health status recorded.

8.2.7. Long-term follow up

Depending on the results at the end of study visit, we may contact participants to enable long-term follow-up (i.e. >1 year for children and >2 years for adults) of immunogenicity for the different vaccine doses. The long-term follow-up will depend on the initial data from this primary study. An amendment to the protocol with details of any planned long-term follow up will be made and submitted to the various regulatory authorities (i.e. SERU, OXTREC and PPB for the Kilifi site, MUST-REC, UNCST and NDA for the Mbarara site) for review and approval.

8.2.8. Qualitative data collection

Qualitative data will be collected from stakeholders to explore the range of perceptions on the use of fractional YF vaccine doses during epidemics in areas where there is vaccine shortage or for routine use. Data will be collected primarily through semi-structured individual interviews as appropriate, with group discussions organized for sub-national level (health managers and providers expected to deliver fractional doses; see Table 3). Following informed consent, the participants will be invited

for individual or group interviews, with questions asked aimed at stakeholders' priorities and concerns regarding the use of fractional doses and their influence on different stages of the policy process (agenda setting, policy formulation and implementation). The national and sub-national regulatory bodies will be sampled from Kenya, Uganda and Senegal.

Figure 1: Policy analysis framework and key stakeholders

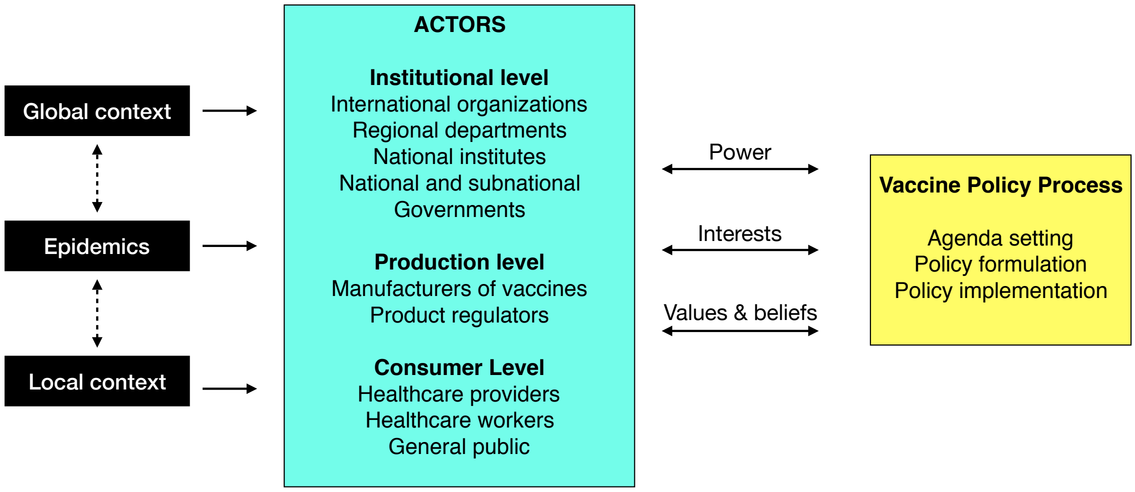

Stakeholders to be sampled in this study

Participant (n)	Affiliation of stakeholder	Method of data collection	Rationale and expertise
**Medical Officer (2)**<br>**Clinical Officer (2)**<br>**Nurse (2)**<br>**Public Health Officer (2)**	Primary Heath Care Providers	FGD, IDI	Implementors of vaccination during epidemics
**County Health Officials (6)**	Implementers of policy	FGD, IDI	Planners of vaccination during epidemics
**PPB (2), NDA (2)**	National Regulatory Authorities	IDI	Regulatory authorities
**Officials (6)**	Vaccine Manufacturers	IDI	YF vaccine manufacturers
**Official (2)**	GAVI, The vaccine Alliance	IDI	Funding of vaccines
**Official (2)**	PATH	IDI	YF strategic demand forecasting
**Official (2)**	SAGE	IDI	Subject experts
**Official (2)**	WHO	IDI	Involved with supply of YF vaccine and interactions with manufacturers, Eliminate Yellow Fever Epidemics (EYE) official
**Researcher/Academic (3)**	Research Institution/University	IDI	Subject experts

*PPB - Pharmacy & Poisons Board, Kenya; NDA - National Drug Authority, Uganda; SAGE - WHO Strategic Advisory Group of Experts on Immunization
IDI - in-depth discussion; FGD - focus group discussion

The framework shown in Figure 1 maps the key stakeholders that are involved and their link to the policy. We will draw our questions and analysis from the stakeholder analysis approach which can be useful to understand actors' perceptions, behavior, intentions, inter-relations and interests in

reference to their past, present positions and future potentials. We will conduct about 25 interviews with the different stakeholders. To ensure accuracy of the data and analysis, we will feedback learning to a sub-group of interviewees as a draft report and seek their views on the key findings and interpretations. Depending on initial data and emerging themes, we may sample more stakeholders at each level of the actors.

9. SELECTION AND WITHDRAWAL OF STUDY PARTICIPANTS

9.1. Description of the population to be studied

The main study will recruit healthy adults living in Kilifi and Mbarara who are willing to participate. The sub-study will recruit healthy children in Kilifi and Mbarara whose parents/guardians are willing to consent their participation in the study.

9.2. Inclusion criteria

- Individuals aged ≥18 - <60 years of age.
  - For the sub-study, children aged between 9 months and 5 years.
- HIV status
  - HIV negative on serological screening OR
  - HIV positive on serological testing, and no symptoms suggestive of current clinical immunosuppression and CD 4 count>200 within the last 6 months.
- Ability to provide informed consent to participate in the study

9.3. Exclusion criteria

- Known contraindications to YF vaccination such as allergies to egg protein or any component of the vaccine (including gelatin, eggs, eggs products or chicken products), immunodeficiency, known thymus disorder, such as thymoma and myasthenia gravis
- Using corticosteroids or other immunosuppressive therapy
- Thymus disorder, such as thymoma and myasthenia gravis
- Acute febrile disease on the day of vaccination with temperature >37.5 degrees Celsius.
- Previous YF vaccination
- Previous YF infection as determined from history
- Pregnancy (as determined by a urine test on the proposed day of vaccination) and lactating women
- Planning to migrate out of the study areas before the end of the study follow-up
- Planning to travel to a country requiring YF vaccination certificate within the first year after vaccination.

9.4. Withdrawal criteria

In accordance with the principles of the current revision of the Declaration of Helsinki, a participant has the right to withdraw from the study at any time and for any reason and is not obliged to give his or her reasons for doing so. In addition, the participant may be withdrawn for any of the following reasons:

- Participant non-compliance with study requirements (for example follow-up visits) despite reminders and attempts to make contact
- Participant moves out of the study area and cannot be traced
- A SAE, which requires discontinuation of the study involvement or results in inability to continue to comply with study procedures

9.5. Managing withdrawals

The study team will continue to follow up all vaccine recipients, with their agreement, until the end of the study wherever possible. The reason for withdrawal will be recorded in the Case Report Form (CRF) if given. If withdrawal is due to a SAE, appropriate follow-up visits or medical care will be arranged, with the agreement of the participant, until the event has resolved, stabilized or a non-study related causality has been established. Following Good Clinical Practice (GCP) guidelines, data on participants who specifically withdraw their consent for use of their data will not be included in the data analysis. However, participants who withdraw from follow up without withdrawing consent for use of their data will be included.

9.6. Replacing withdrawn participants

Participants that withdraw from the study or withdraw their consent will not be replaced.

10. TREATMENT OF STUDY PARTICIPANTS

10.1. Treatments

The investigational product will be one of the four doses of the YF vaccine.

10.2. Identity of Investigational Product

We will use YF vaccine produced by Institut Pasteur de Dakar, Senegal. The product will be a freeze-dried preparation of live attenuated YF virus, sub-strain 17D-204. One vial format will be provided. It will contain standard full dose of vaccine for administration in 0.5ml per dose. A second vial will be prepared from a standard vial to contain vaccine diluted to approximately 1000 IU/dose for administration in 0.5ml per dose. Fractional volumes of this second vial will be administered as per manufacturer's instructions to achieve the 500 IU and 250 IU doses. A single vaccine batch will be used in this study

10.3. Storage

YF vaccine will be stored and transported at a temperature ranging between +2°C and +8°C as per manufacturers' instructions. The vaccine is sensitive to light and needs to be protected from sunlight. The vaccine vials and diluent will be transported together. Vaccine will be reconstituted solely with

the diluent provided by the manufacturer. The diluent provided with the vaccine needs to be between +2°C and +8°C at the time of reconstitution. For this, diluents need to be placed in the refrigerator at least one day (24h) before its use. Reconstituted YF vaccine is heat labile hence vials will be discarded 6 hours after reconstitution. The specific manufacturer storage and reconstitution instructions indicated in the product insert will be followed.

Temperatures in the cold chain will be monitored regularly through the day to avoid any deviations. Where temperature excursions occur, they will be resolved with the sponsor following the manufacturer's recommendations. The reconstitution and discard hours will be recorded in the vaccine administration form. The YF vaccines are attached with a vaccine vial monitor type 14 (VVM14), which means the vaccines can withstand cumulative exposure to 37°C for up to a period of 14 days and still retain potency.

10.4. Dose selection

Standard dose of vaccine will be administered in 0.5ml per dose. For the lower doses, a second vial will be prepared from a standard vial, by the manufacturer, to contain vaccine diluted to approximately 1000 IU/dose for administration in 0.5ml per dose. Fractional volumes of this second vial will be administered as per manufacturer's instructions to achieve the 500 IU and 250 IU doses. A single vaccine batch will be used in this study.

10.5. Timing of Doses

The vaccine will be administered once during the vaccination visit.

10.6. Randomization and Blinding

Each participant will be randomized to receive one of the four vaccine doses. The allocation will be to one of the four treatment arms per a computer-generated randomization schedule. Randomization will be done by randomization booklets with concealed scratch cards, allocated in order of recruitment and opened on the day of vaccination.

The children will be randomized to either full dose or the lowest non-inferior dose of the vaccine. The vaccine dose to be used will be selected based on the safety and immunogenicity results at day 28 post-vaccination from the adult study.

The vaccinating nurse and pharmacist will be unblinded to the allocation of the vaccine doses. They shall not disclose the allocations to any of the team members and they will not be involved with follow up the participants post vaccination. The rest of the trial team will be blinded throughout the study.

10.7. Dispensing Procedures

Study vaccines will be labelled specifically for the study. Vaccines will be reconstituted once the first participant of the day has been randomized to a specific vaccine dose. The pharmacist together with the vaccinating nurse will be responsible for preparation and administration of the vaccines as randomised. They will be responsible for the accompanying documentation.

10.8. Dose Administration

The vaccine dose to be administered will be guided by the randomization process. The doses to be evaluated will be the full standard dose, 1000 IU, 500 IU and 250 IU. After reconstitution according to the manufacturer's instructions the full dose and the 1000 IU will each be in a volume of 0.5ml. For the 500 IU and 250 IU doses, fractional volumes as per instructions from the manufacturer will be administered. Vaccine will be administered subcutaneously. As multi-dose vials will be used, the aliquot number (1,2,3, etc.) will be documented on the CRF. The YF vaccine contains no preservative; hence the repeated manipulation for vaccine reconstitution and withdrawal could lead to a greater risk contamination of the vial contents. The pharmacist and vaccinating nurse will prepare the vaccine using aseptic techniques.

10.9. Unblinding

Unblinding will be done at the end of the trial. All participants will be encouraged to have a YF vaccine regardless of the randomization arm if required for travel or in case of an epidemic. Hence unblinding will not be necessary unless as per DSMB request for SAE review.

10.10. Prior/Concomitant Therapy

Concomitant therapy will be documented. Previous YF vaccine or planned YF uptake in the next 24 months will be noted for exclusion.

11. LAB PROCEDURES

11.1. Screening assessments

HIV serology will be conducted using rapid diagnostic tests following the Governments of Kenya and Uganda guidelines. Pregnancy will be determined using a urine test kit in accordance with manufacturer's instructions.

11.2. Research assessments

Venous blood samples (10mls for adults, 6mls for children) will be collected at baseline, 10 days, 28 days, 1 year and 2 years (adults study only) post-vaccination as outlined in Table 2. For the children's study, study staff skilled in taking blood from children will be responsible for the phlebotomy. The 6ml blood sample volume for children is lower than the limit specified by existing pediatric guidelines, ranging from 1–5% of total blood volume within 24 hours and up to 10% of total blood volume over 8 weeks (22).

Serum will be isolated from blood used for virus neutralization assays ($PRNT_{50}$). PRNT is considered the most sensitive and specific test for quantification of neutralizing antibodies and is the reference method for assessing immune response after vaccination. Peripheral blood mononuclear cells (PBMCs) will also be isolated from the blood samples for assessment of cellular immune responses. Viral RNA will be isolated from serum for detection YF vaccine virus by qRT-PCR [20] on days 0, 2, 3, 4, 5, 6, 7 and 10 (Table 2). For this purpose, all individuals will be sampled on days 0 and 10 and randomized to provide an additional sample on either day 2, 3, 4, 5, 6 or 7. This sparse sampling approach will allow detection and modeling of YF vaccine virus levels in blood, by study arm, whilst minimizing the number of samples taken per individual.

Processing of blood will be done within 24 hours of sample collection at the KEMRI CGMRC immunology lab and at the Epicentre laboratory at the Mbarara University of Science and Technology (MUST) in Mbarara. Samples will be stored at the KEMRI CGMRC and Epicentre Mbarara repositories. The $PRNT_{50}$ assay will be conducted at the WHO approved reference laboratory at Institut Pasteur de Dakar in Senegal using standard techniques. The lab is performing the $PRNT_{50}$ assays for the ongoing trial (Clinicaltrials.gov number: NCT02991495) thus allowing comparability of the data generated in this study with that from the ongoing trial using a single $PRNT_{50}$ assay.

Institut Pasteur de Dakar in Senegal will also be providing the vaccine. However, there will be no conflict of interest, as the vaccine production unit will supply the vaccine while the lab unit will conduct the $PRNT_{50}$ assays, which are two separate entities. Moreover, the lab personnel will be blinded to the vaccine dose allocations.

Baseline samples will be evaluated for presence of antibodies to other flaviviruses and their association with vaccine immunogenicity assessed. Assessment of the cellular immune response to YF (using PBMCs and measurement of cytokines in serum) and neutralising antibodies against other flaviviruses will be done at KEMRI CGMRC.

YF vaccine virus detection by qRT-PCR will be done at the WHO approved reference laboratory at Uganda Virus Reseach Institute (UVRI) in Entebbe. These immunological and molecular assessments will be undertaken alongside training of research assistants at both trial sites. Permission will be sought from SERU and Ugandan ethical committees to allow sample shipment to collaborating research scientists at the WHO approved reference laboratories at Institut Pasteur de Dakar in Senegal, UVRI in Entebbe, Uganda and KEMRI CGMRC, Kenya. All specimens will be shipped in accordance with IATA specimen shipping regulations for infectious materials. Results from the $PRNT_{50}$ and qRT-PCR assays will be provided to KEMRI CGMRC for verification and for data entry into the KEMRI CGMRC database.

We will establish a network for quality control and standardization of $PRNT_{50}$ and qRT-PCR assays including UVRI, Institut Pasteur de Dakar, the National Institute for Biological Standards and Control (NIBSC), UK and the Robert Koch Institute (RKI, Germany). All four institutions have expertise in $PRNT_{50}$ and qRT-PCR assays. We will obtain defined assay standards from RKI and/or NIBSC will share defined standards to confirm the reproducibility of results and distributions will be conducted once per year. If results are not comparable then further exchanges will be planned to harmonize protocols, and consistency in results will be achieved before the clinical trial progresses.

12. ASSESSMENT OF SAFETY

12.1. Adverse Events (AEs)

SAEs and AE will be recorded in all participants up to day 28. Passive assessment of SAEs will continue during the 2-year follow-up and will be reported.

Adverse events of special interest will be monitored. These will include:

- Injection-site local reaction occurring within 7 days following vaccine administration

While the following adverse events are rare, they will be monitored during the study.

- Vaccine-associated viscerotropic disease (YEL-AVD)
- Vaccine -associated neurotropic disease (YEL-AND)

12.2. Definitions and monitoring of AEs

AEs and SAEs will be defined in accordance with the International Conference on Harmonization (ICH) Guidelines for Good Clinical Practice (38):

An adverse event is defined as any untoward medical occurrence in a clinical trial subject to whom a vaccine has been administered; it does not necessarily have a causal relationship with the vaccine/vaccination. Definitions of AEs and grading, when applicable, to be followed in both study sites will be specified in an SOP. The definition of AE indicates a change in the participant’s health status ‘since baseline’ right before the study vaccine is administered. To assess AE, we’ll consider pre-existing conditions and concomitant medication taken prior to vaccination or during the follow-up period.

A serious adverse event is any untoward medical occurrence that:

- Results in death
- Is life threatening: if the participant was at risk of death at the time of the event; it does not refer to an event that hypothetically might have caused death if it were more severe
- Results in persistent or significant disability/incapacity: if the event results in a substantial disruption of the participant's ability to carry out normal life functions. This definition is not intended to include experiences of relatively minor medical significance such as headache, nausea, vomiting, diarrhea, influenza, injection site reactions and accidental trauma (e.g. sprained ankle)
- Requires in-patient hospitalization or prolongation of existing hospitalization: in general, hospitalization signifies that the participant has been detained (usually involving at least 24h stay) at the hospital or emergency ward for treatment that would not have been appropriate in an outpatient setting
- Is a congenital anomaly/birth defect in the offspring of a study participant
- Is an important medical event that may jeopardize the participant or may require intervention to prevent one of the other outcomes listed above should be considered serious.

Hospitalization for either elective surgery related to a pre-existing condition, which did not increase in severity, or frequency following initiation of the study, or for routine clinical procedures (including hospitalization for "social" reasons) are not considered as SAEs. When in doubt as to whether “hospitalization” occurred, or was necessary, the AE will be considered serious. The definition of a routine clinical procedure is a procedure, which may take place during the study period and should not interfere with the study vaccine administration or any of the on-going protocol specific procedures. If anything, untoward occurs during an elective procedure and satisfies any of the criteria for SAE, this will be documented and reported.

The AEs and SAEs will be assessed for linkage to the vaccination at every contact and this will follow standard operating procedures. All AEs and SAEs will be managed according to the standard care by the study team and/or referred to the most appropriate facilities for specialized care.

12.3. Documenting AEs

Both solicited or unsolicited AEs will be recorded on the participant's CRF. The diagnosis, date and time of onset, outcome, severity and relationship to vaccination will be established. Details of any treatment or concomitant interventions will be recorded.

12.4. Reporting Serious Adverse Events (SAEs) and/or Unexpected AEs

The principal investigator (PI) (or designee) is responsible for reporting and providing updates of SAEs/SUSARs to the Sponsor. The sponsor will report SAEs/SUSARs to the vaccine manufacturer. Adverse events will not be reported but will be recorded in the CRFs and summarised in a 6-monthly safety report. The Sponsor (or designee) will coordinate the safety monitoring and reporting in the study. All SAEs identified up to day 28 will be tabulated and reported in summary form for each population. Expedited reporting of individual SAEs will only be undertaken where they are suspected to be causally linked to vaccination and within 28 days of vaccination but will be compliant with the various local requirements as outlined in Table 4. On completion of vaccination of each population (i.e. adults and children), the Sponsor (or designee) will provide summary reports. This report will summarize the SAE data, diagnosis, causality assessment and outcome. Summary reports will be sent to the DSMB members, investigators, ERCs, PPB, NDA and vaccine manufacturer as per their recommendations

Table 4: Expedited reporting matrix

Reported to	Reported by	Timeline
Sponsor (or designee)	PI (or designee)	Within one working day of becoming aware of the vaccine-linked SAE
KEMRI-SERU (Kenya) and MUST-REC and UNCST (Uganda)	PI (or designee)	Within 48 hours of notification for study-related events. Within 10 working days of notification for non-related events.
OXTREC	PI (or designee)	Within the timeline established by their procedures.
PPB (Kenya) and NDA (Uganda)	PI (or designee)	Within 7 calendar days of notification.
DSMB	Sponsor (or designee)	Within one working day of the sponsor becoming aware of the vaccine-linked SAE

12.5. Emergency Procedures

During vaccination, staff trained in basic life support will be available in case of adverse reactions around vaccination. An emergency kit will also be available and checked routinely.

12.6. Pregnancy

Female participants becoming pregnant during the first 28 days following vaccination will be followed until the end of the pregnancy and the outcome will be recorded. This will be reported as an AE as the vaccine is not recommended in pregnant women. The outcome of the pregnancy (live birth, still birth or abortion) will be recorded after birth. Subsequent reports containing follow-up information regarding a pregnancy is not required unless the pregnancy results in a congenital anomaly. The congenital anomaly should be promptly reported as a Serious Adverse Event.

12.7. Procedures for reporting any protocol violation(s)

Protocol violations will be reported to the sponsor, regulatory and ethics committees as specified in their guidelines.

13. STATISTICS

13.1. Determination of sample size

This study will be powered to detect non-inferiority of each lower dose of vaccine (1000 IU, 500 IU, 250 IU) compared to the full standard vaccine dose. Sample size calculations were done using *art2bin* on Stata version 15.

For the adult study, we assumed a 95% seroconversion rate, 90% power, 2.5% alpha for a one-sided test and a non-inferiority margin of 10%, which gave a sample size of 100 per arm. The 10% non-inferiority margin was chosen in consideration of the public health consequence of a loss of protection but a potential increase in vaccine dosages in a situation where vaccine stocks are insufficient to respond to an outbreak. The sample size was increased by 20% to account for: i) losses to follow up and, ii) unevaluable participants with a positive serological response for YF virus at baseline. Thus, a total sample size of 480 will be required for the four vaccine dose groups (i.e. full dose, 1000 IU, 500 IU and 250 IU; Table 5).

For the study in children, we assumed a 90% seroconversion rate (accounting for lower vaccine immunogenicity reported in children [4]), 90% power, 2.5% alpha for a one-sided test and a non-inferiority margin of 10%, which gave a sample size of 190 per arm. This was increased by 10% to account for 5% losses to follow up and 5% unevaluable participants with a positive serological response for YF virus at baseline. This gave a total sample size of 420 (i.e. 210 in the full dose group and 210 in the lower dose group; Table 5).

Analyses of all other efficacy and safety endpoints are secondary outcomes. Therefore, no significance adjustments will be made for multiple comparisons.

Table 5: Planned participant recruitment numbers in Kilifi and Mbarara

Population	Allocation	Participants in Kilifi (n)	Participants in Mbarara (n)	**Total per allocation**
Adults (N=480)	Full dose	60	60	**120**
	1000 IU	60	60	**120**
	500 IU	60	60	**120**
	250 IU	60	60	**120**
Children (N=420)	Full dose	105	105	**210**
	To be determined	105	105	**210**

13.2. Statistical and analytical plans

13.2.1. Data management

A Data Management team will be located at each study site. Data collection will occur at each study site using standardized CRFs transported by designated staff. Data entry clerks will use password-protected computers. Data will be double entered at each study site.

The CRFs in this study will be entered onto an electronic database at each study site. This will be done via secure web interface with data checks used during data entry to ensure data quality. The database will be activated for the study only after successfully passing a formal design and test procedure. Laptops and desktop computers will be used for data entry of paper forms. Management and maintenance of computers will lie with the operational support and data managers at the study site.

13.2.2. Data security, access and backup

The database will be kept in a locked server-room. Only the system administrators have direct access to the server and back-up tapes. A role concept with personal passwords (site investigator, statistician, monitor, administrator etc.) regulates permission for each user to use the system and database, as he/she requires.

All data entered onto the CRFs are transferred to the database using Secure Sockets Layer (SSL) encryption. Each data point has attributes attached to it identifying the user who entered it with the exact time and date. Retrospective alterations of data in the database are recorded in an audit table. Time, table, data field and altered value, and the person are recorded (audit trail). A multi-level back-up will be implemented. Back-ups of the entire system including the database are run internally several times per day and on external tapes once a day. The back-up tapes are stored in a secure location.

13.2.3. Analysis of immunological endpoints

Information for each of the following categories will be presented for the adult study and the children sub-study: number of individuals screened for eligibility; the number and reason of screen failures; the number and percentage of eligible individuals who consent and are randomized; the number and percentage of randomized individuals who receive a vaccine; the number and percentage of vaccinated individuals who complete a day 10 post-vaccination visit; the number and percentage of vaccinated individuals who complete a day 28 visit; the number and percentage of vaccinated individuals who complete a 12 month visit; the number and percentage of vaccinated individuals who complete a 24 month visit; the number and percentage of vaccinated individuals who complete the final analysis period (defined as completing all study activities up to 12 months for the children sub-study, and up 24 months for the adult study); and the number of individuals who discontinue and the reason for discontinuation. Participant demographics and baseline characteristics will be described and compared between vaccine groups using non-parametric tests.

The intention to treat (ITT) population will comprise all randomized participants who received a dose of a study vaccine and that have at least one post-vaccination blood sample. The per protocol (PP) population will include randomized participants who have a blood sample at baseline and 28 days (+/- 3 days) post-vaccination, who are seronegative ($PRNT_{50}$ <1:10) to YF at baseline, and for whom the eligibility criteria were correctly applied. The safety population will include all subjects who received a study vaccine.

The primary analysis will be a pairwise statistical comparison of the rate of seroconversion at day 28 between full dose and each lower dose of vaccine using a non-inferiority test with a margin of non-inferiority of 10% in the PP population. Seroconversion will be defined as a ≥4-fold rise in $PRNT_{50}$ titre between day 0 and day 28 samples. Any $PRNT_{50}$ value reported as below the Limit of Quantification (LOQ) (e.g. <1:10) will be converted to LOQ/2. Thus a 4 rise for a subject who is <1:10 at baseline, is a titre of 20. Each immunogenicity assessment will be a pairwise comparison of the full dose and each lower dose within one study population (i.e. adults or children).

Secondary analyses will include assessment of seroconversion in the ITT population as a whole, on the subset of the ITT population with baseline seropositivity to YF, and in the subset of the PP population with no reported history of flavivirus infection. Geometric mean $PRNT_{50}$ titre (GMT) and GMT fold increase (GMFI) and corresponding 95% confidence intervals (CI) on day 0 and 28 will be calculated. A test of non-inferiority will be performed for the difference in GMT and GMFI between the full dose and each lower dose group. Titres will be graphically represented by reverse cumulative distributions obtained by plotting, for each possible value of the titre (abscissa), the proportion of subjects with a titre greater than this value.

Lower vaccines doses may change the kinetics of antibody response. The assessment of seroconversion rates, GMT, and GMFI 10 days after vaccination in the ITT population will provide important information in the context of low dose vaccine usage in outbreak response. These three immunogenicity outcomes will also be assessed at 1 year and 2 years post-vaccination in the ITT population to confirm a lasting effect of full and low dose vaccination.

Adverse events occurring during the study follow up period will be analysed and compared between groups. This will be a descriptive analysis and will include all AEs up to 28 days post-vaccination, and SAEs that occurred any time during study follow-up.

A detailed statistical analysis plan (SAP) will be provided separately and finalized after the study has started (and before the database lock). This SAP will include all conventions on data, descriptive and statistical analyses to be performed on collected data during the conduct of the study.

13.2.4. Qualitative Data Analysis

Interviews will be conducted in English, audio-taped and transcribed. Audio recordings of the FGDs will be transcribed and the subject identifying information omitted. Analysis will use a thematic framework aiming to identify and categorize the attitudinal, and contextual factors in regard to the policy. The semi-structured in-depth interviews will include topics that influence decision-making in policy. Analysis of qualitative data will follow the framework approach. To ensure that interpretations of quotes are consistent, and that data quality is rigorous and transparent, independent qualitative researchers will code the data; differences between coding will be resolved by group discussion involving other members of the research team. Recurring issues, concepts and patterns will be identified using ground theory and grouped according to thematic areas.

14. DIRECT ACCESS TO SOURCE DATA/DOCUMENTS

The trial team in KEMRI CGMRC and Epicentre, Mbarara will hold source documentation securely. Access will be granted to monitors responsible for quality assurance, for data entry staff and for purposes of medical care. Access will also be granted for audit or inspection by statutory authorities and other relevant persons. Non-study team members will not be granted access. Qualified staff will supervise data collection and entry on a regular basis. Data managers will support onsite data entry teams.

15. QUALITY CONTROL AND QUALITY ASSURANCE

15.1. Monitors and monitoring plan

The study will be conducted in accordance with the current approved protocol, ICH GCP, relevant regulations and standard operating procedures. Monitoring will be performed as per ICH GCP including but not limited to regular visits during the clinical study and a closeout visit. Data will be evaluated for compliance with the protocol and accuracy in relation to source documents. Following written standard operating procedures, the monitors will verify that the clinical study is conducted, and data are generated, documented and reported in compliance with the protocol, GCP and the applicable regulatory requirements. Monitoring will be performed internally at each study site. Moreover, external monitoring visits will be conducted in coordination with the sponsor. Monitors will participate in all key, planned activities, collaborating with implementation of the study. The monitoring plan is detailed in the Study Monitoring SOP that will be prepared prior to the study. The SOPs will cover instructions for monitoring the main aspects of the implementation of the clinical studies, as follows:

- Study authorizations and approvals and communication with the ethics committees
- Duties and Responsibilities of the Investigator/Institution
- Suitable resources
- Medical care for the participants
- Clinical Study compliance with the agreed protocol
- Laboratory aspects
- Informed consent of participants
- Data management, records and reports
- Sponsor 's Responsibilities
- Responsibilities of the Monitors
- Records of GCP training

16. INTELLECTUAL PROPERTY

The vaccine product assessed in the clinical trial is fully licensed and in routine use, and therefore no product-related IP will arise from this trial. In the unexpected event that other findings lead to IP then KEMRI guidelines will be followed in terms of registering and exploiting the IP. However, any intellectual property rights that arise from the work will be safeguarded as per the KEMRI 2015 IPR guidelines and the Industrial Property Act of 2001, sections 32, 58 and 80. The scientific and intellectual contributions of all persons involved in the research will be appropriately acknowledged in all publications and presentations arising from the work.

17. TIME FRAME/DURATION OF THE TRIAL

The total study duration is 40 months. However, study timelines will be determined after receipt of approval from KEMRI SERU, PPB, OXTREC, MUST-REC, UNCST and NDA. Estimated timelines are shown below.

Activity	Time period following ethical approval
Study staff recruitment, procurement etc.	Month 1 - 6
Community engagement and mobilization	Month 1 - 3
Screening, vaccinations and follow up for adult study	Month 3 - 27
Screening, vaccinations and follow up for children sub-study	Month 12 - 30

Sample processing and laboratory assays ($PRNT_{50}$, qRT-PCR)	Month 3 - 39
Qualitative data collection and analysis	Month 6 - 30
Feedback to study participants, complete data analysis and write up	Month 30 – 40

18. ETHICS

In Kenya, ethical approval will be sought from KEMRI SERU, OXTREC and regulatory approval from PPB for Kilifi. In Mbarara, ethical approval will be sought from MUST-REC and UNCST and regulatory approval from the Uganda NDA. Individual consent will be obtained from each of the study participants.

18.1. Human Subjects

i. "First, do no harm."

The YF vaccine to be used is a licensed product. This, and all other licensed YF vaccines are all derived from the 17D YF virus strain. Tens of millions of YF vaccine doses have been used globally. Adverse reactions to the vaccine are generally mild and include headache, myalgia, malaise and asthenia in around 10-30% of participants during the first few days after vaccination.

Serious reactions are very rare and include hypersensitivity reactions generally associated with egg protein but may also implicate the gelatin used by some manufacturers, vaccine-associated neurological diseases (YEL-AND) and vaccine-associated viscerotropic disease (YEL-AVD), which can vary from multi-organ system failure without much evidence of hepatitis to a fulminant hepatitis resembling wild-type yellow fever. YEL-AVD is a very rare event occurring after a 1st dose of YF vaccine. This occurs within 10 days of vaccination and is characterized by severe multi-organ failure. Known risk factors include a history of thymus disease (e.g. thymoma or thymectomy) and age ≥60 years. We will exclude these groups from vaccination.

Monkey studies have suggested an inverse relationship between YF vaccine dose and the magnitude and duration of vaccine viremia [18, 19]. However, whilst more studies are needed to assess the impact of low vaccine doses on viremia, a previous fractional dosing trial in adults in Brazil found no association between viremia levels and vaccine dose [16]. We will monitor post-vaccination viremia as part of this study.

18.2. Community Considerations

We will use existing community engagement strategies to inform communities about the study where we will involve the local sub-national Health Management teams, local administration and the community members. A community engagement plan specific for the study will be developed between the Community Engagement teams and the investigators. Meetings with institutional heads, chiefs, community leaders and community representatives will be organised to explain the study and its aims, and to discuss concerns.

Research findings will be disseminated to participants and the participating communities upon completion of the study using the appropriate networks and specific meetings. Study participants will be encouraged to take a YF vaccine in the context of an epidemic or travel if this occurs before the unblinding of the study results. Once unblinded, subjects will be notified of the vaccine dose received and the implications for protection in future based on their allocated group. However, the study team will not provide individual-level immunogenicity data to participants, as the assays are not validated for individual-level prediction of protection. Further, the study team will not be able to provide a YF vaccination certificate for travel or as proof of protection to those who have received the full dose. However, on completion of the study we will offer to pay for the cost of re-vaccination at an authorized public health facility for any study participant who would like to receive a YF vaccination certificate. No safety concerns are reported with multiple doses of the vaccine.

18.3. Informed Consent

Meetings will be organized with potential volunteers where investigators will discuss the study objectives, including the risks and benefits of participation, and describe the inconvenience and procedures required for participation in detail. All volunteers will sign and date the informed consent form before any study specific procedures are performed in confidential spaces with a trained member of the study team. All informed consent documents will be translated into Kiswahili and Runyankore.

We will emphasize the following:

- Participation in the study is entirely voluntary.
- Declining to participate involves no penalty or loss of medical benefits.
- A volunteer may withdraw from the study at any time.
- A volunteer is free to ask questions at any time to allow him or her to understand the purpose of the study and the procedures involved.
- There is no direct benefit from participating. The benefits will be realized in the long-term for the community by contributing towards the development of understanding the use of these yellow fever vaccines in different ways.
- Volunteers will be compensated for travel, time and inconvenience of participating.

18.4. Compensation

We will reimburse participants for travel required to attend screening and to attend for vaccinations and follow up. Reimbursement for out of pocket expenses will be done in accordance with standard figures determined by the respective institutional (KEMRI CGMRC and Epicentre) community engagement teams based on government recommendations on daily wages for skilled and non-skilled labor in Kenya and Uganda.

18.5. Patient Data Protection/Confidentiality

Clinical records will be kept in locked cabinets in the clinical trials facilities at each of the sites. All immunological and qRT-PCR data will be kept in anonymized databases linked by the study number to clinical data.

18.6. Data Sharing

The study will be conducted across two sites (Kilifi and Mbarara) and anonymised data will be entered into a database maintained by KEMRI CGMRC. Individual-level anonymised data will be shared with the vaccine manufacturer and with medical regulators including the WHO for pre-qualification, regulatory and policy purposes. For wider engagement of stakeholders, including the medical community, summary-level statistical analyses will be shared. Information collected or generated during this study may be anonymised for use to support new research and policies for YF vaccine. Any future research using information from this study will need approval from a local or national expert committee to make sure that the interests of participants and their communities are protected.

18.7. Safety

The study team will provide medical care to participants during the study follow-up period for acute illnesses. The study team will not become responsible for long-standing chronic conditions that were present before vaccination, or those that are unrelated to vaccination, and medical care will be provided within the respective (Kenyan or Ugandan) Ministries of Health guidelines.

18.8. Material Transfer Agreement (if applicable)

A Material Transfer Agreement (MTA) will be developed before shipping of samples to the WHO approved reference laboratories at Institut Pasteur, Dakar in Senegal, UVRI in Entebbe, Uganda, and KEMRI CGMRC, Kenya, and for shipping of standards from NIBSC and RKI. This will include the following information.

- Identification of the provider and recipient
- Identification of the material and the volume of material
- Definition of the trial and how the material will and will not be used.
- Maintenance of confidentiality of background or supporting data or information, if any.

19. ARCHIVING AND RECORD RETENTION

19.1. Overview

A Data Management team will be located at each study site. Data collection will occur at each study site using standardized CRFs. The data managers will lead and support these activities.

19.2. Investigator site file (ISF)

The investigators will maintain appropriate medical and research records for this study, in compliance with ICH E6 GCP, GDPR, regulatory and institutional requirements for the protection of confidentiality of participants. The principal investigators, co-investigators, and clinical research staff will have access to records. The investigators will permit authorized representatives of the Sponsor, and regulatory agencies to examine (and when required by applicable law, to copy) clinical records for the purposes of quality assurance reviews, audits and evaluation of the study safety and progress.

The ISF will be maintained at the study site containing at least the following documents and information:

- Signed protocol and amendments
- CRFs
- Current informed consent form and all revisions
- Current participant information sheet and all revisions
- Any other written information given to the study team
- Financial aspects of the study
- Insurance statement
- All signed agreements/contracts
- Dated and documented approval of ethics committee and regulatory authorities
- Signed CV's of all investigators and any study personnel (updated regularly as changes occur)
- Monitoring reports
- Relevant communication
- Signed informed consent forms
- Signed, dated, and completed CRFs
- SAE reporting
- Notification by Sponsor of safety information
- Annual reports to ethics committee and regulatory authorities
- Participant screening log
- Participant identification code list (mapping patient onto anonymized study ID)
- Participant enrolment log
- Investigational product accountability
- Authorization/signature sheet
- Clinical study report

19.3. Source documents

All protocol required procedures along with information necessary to report the observations and tests described in this protocol are recorded in CRFs. Any entries captured on CRFs that are derived from source documents e.g. hospital record, will have the source documents included as part of the participant's file. Where source documents for specific entries are not available, this must be explicitly mentioned. Any requested information that is not obtained as specified in the protocol should have an explanation noted on the CRF as to why the required information was not obtained.

CRFs will be completed and signed and authorized in a timely and accurate manner by designated study staff within a month of completion of a visit. All data on the CRFs must be legibly recorded in ink. The investigator or a designated, qualified individual must review all CRFs for accuracy and consistency with any relevant source documentation and sign the CRFs upon completion. Any corrections will be made on the CRFs by striking through the incorrect entry with a single line and entering the correct information adjacent to it. The correction will be initialed and dated by the investigator or a designated, qualified individual. Any corrections made after data entry has begun will be notified to data managers for correction of electronic databases.

19.4. Record keeping and retention

The ISF including a copy of the final completed CRFs, as well as all source documentation is retained by the investigator and one copy will be maintained by the Sponsor, who will ensure that it is stored with other study documents, such as the signed informed consent forms, protocol, the investigator's brochure and any protocol amendments, in a secure place following local regulations.

The Sponsor will securely store the final study database with all archive tables for at least 10 years. The Sponsor also keeps the central Trial Master File and interim and final reports both in electronic and in hard copy form for at least 10 years. Sites will archive paper CRFs and study files following local laws.

20. FINANCING AND INSURANCE

20.1. Budget

Item	USD	KES	UGX
Personnel, salaries and benefits disbursements	800,000	80,800,000	2,997,600,000
Patient costs, travel, food and/or supplies	200,000	20,200,000	749,400,000
Equipment	16,000	1,616,000	59,952,000
Community engagement	20,000	2,020,000	74,940,000
Supplies			
Laboratory supplies	220,000	22,220,000	824,340,000
Clinical supplies	100,000	10,100,000	374,700,000
Shipping costs	12,000	1,212,000	44,964,000
Travel and accommodation			
Local	10,000	1,010,000	37,470,000
International	15,000	1,515,000	56,205,000
Trial monitoring & DSMB meetings	20,000	2,020,000	74,940,000
Transportation, vehicle repairs etc.	46,000	4,646,000	172,362,000
Operating expenses postage, printing etc.	12,000	1,212,000	44,964,000
TOTAL	1,471,000	148,571,000	5,511,837,000
15% contingency	220,650	22,285,650	826,775,550
**GRAND TOTAL**	**1,691,650**	**170,856,650**	**6,338,612,550**

20.2. Justification of the Budget

This work has been funded by the European and Developing Countries Clinical Trials Partnership (EDCTP). The budget includes support for all trial related activities, including community engagement. The study will not incur any consultancy fees or additional administrative overheads. Costs for patients and supplies are based on those incurred by similar vaccine trials. We have recruited laboratory research assistants who will be trained on the relevant virological and immunological studies. The clinical staff on the project will undertake Good Clinical Practice training as well as on the job training for running clinical trials.

20.3. Insurance

The vaccine manufacturer is liable for any harm arising from negligent manufacture but has not undertaken to sponsor the trial. The sponsor, University of Oxford, provides insurance to cover the clinical trial participants. KEMRI CGMRC and Epicentre, Mbarara will provide indemnity for any clinical negligence at their respective sites.

21. TRIAL MANAGEMENT

University of Oxford/KEMRI CGMRC takes responsibility for initiating, registering and conduct of the trial, and as such, will be involved in the study design, collection, management and analysis, and interpretation of data, and writing of the report. The Sponsor takes responsibility for ensuring the trial is monitored properly and results made available.

Trial PIs in each site (i.e. KEMRI CGMRC, as covered by this protocol, and Mbarara for Epicentre) will communicate regularly to coordinate on any challenges experienced by either site or necessary amendments requiring ethical review. A DSMB will be convened by the Sponsor and will receive safety data as described above.

22. REPORTING, DISSEMINATION AND NOTIFICATION OF RESULTS

Results will be published in a journal providing an open-access option. Anonymized data on immunogenicity and other trial outcomes will be made available within these publications. We will feedback individual results with clinical relevance to participants in real-time. We will feedback individual randomization arm to participants once the trial has been completed and the study team unblinded. Summaries of the outcomes of the trial will be provided during community meetings in the areas from which participants are recruited.

23. APPENDICES

23.1. Roles of Investigators

INVESTIGATOR	INSTITUTION	ROLE
**George Warimwe**	KEMRI CGMRC	PI. Design, conduct, data quality control and assurance. Analysis, interpretation of results and publication.
**Derick Kimathi**	KEMRI CGMRC	Co-PI, DPhil student- University of Oxford and Study Lead. Design, conduct, recruitment, safety monitoring, data collection, sites coordination, interpretation of results and publication.
**Philip Bejon**	KEMRI CGMRC	Sponsor's representative.
**Sassy Molyneux**	KEMRI CGMRC	Qualitative study, design, analysis, interpretation of results and publication.
**Mainga Hamaluba**	KEMRI CGMRC	Head of Clinical Trials facility, data quality control, assurance and analysis, interpretation of results and publication.
**Henry Karanja**	KEMRI CGMRC	Sample preparation and storage, lab assays and analysis, interpretation of results and publication.
**John Gitonga**	KEMRI CGMRC	Sample preparation and storage, lab assays and analysis, interpretation of results and publication.
**Marianne Munene**	KEMRI CGMRC	Regulatory affairs contact, interpretation of results and publication
**Jackline Wafula**	KEMRI CGMRC	Project management and coordination
**Patrick Kazooba**	Epicentre, Mbarara	Site investigator (Mbarara) and study team lead. Design, conduct, recruitment, safety monitoring, data collection, site coordination, interpretation of results and publication.

**Juliet Mwanga**	Epicentre, Mbarara	Director, MSF Epicentre, Mbarara. Data quality control, assurance and analysis, interpretation of results and publication.
**Dan Nyehangane**	Epicentre, Mbarara	Sample preparation and storage, lab assays and analysis, interpretation of results and publication.

23.2. References

1. Shearer FM, Longbottom J, Browne AJ, Pigott DM, Brady OJ, Kraemer MUG, Marinho F, Yactayo S, de Araujo VEM, da Nobrega AA *et al*: **Existing and potential infection risk zones of yellow fever worldwide: a modelling analysis**. *Lancet Glob Health* 2018, **6**(3):e270-e278.
2. Barrett AD, Higgs S: **Yellow fever: a disease that has yet to be conquered**. *Annu Rev Entomol* 2007, **52**:209-229.
3. Monath TP, Barrett AD: **Pathogenesis and pathophysiology of yellow fever**. *Adv Virus Res* 2003, **60**:343-395.
4. Gotuzzo E, Yactayo S, Cordova E: **Efficacy and duration of immunity after yellow fever vaccination: systematic review on the need for a booster every 10 years**. *Am J Trop Med Hyg* 2013, **89**(3):434-444.
5. Garske T, Van Kerkhove MD, Yactayo S, Ronveaux O, Lewis RF, Staples JE, Perea W, Ferguson NM, Yellow Fever Expert C: **Yellow Fever in Africa: estimating the burden of disease and impact of mass vaccination from outbreak and serological data**. *PLoS Med* 2014, **11**(5):e1001638.
6. Shearer FM, Moyes CL, Pigott DM, Brady OJ, Marinho F, Deshpande A, Longbottom J, Browne AJ, Kraemer MUG, O'Reilly KM *et al*: **Global yellow fever vaccination coverage from 1970 to 2016: an adjusted retrospective analysis**. *Lancet Infect Dis* 2017, **17**(11):1209-1217.
7. World Health Organization (WHO): **Yellow Fever Situation Report**. In.; 2016.
8. Pan American Health Organization (PAHO): **Epidemiological Update: Yellow Fever**. In.; 2018.
9. Beck AS, Barrett AD: **Current status and future prospects of yellow fever vaccines**. *Expert Rev Vaccines* 2015, **14**(11):1479-1492.
10. World Health Organization (WHO): **Recommendations to assure the quality, safety and efficacy of live attenuated yellow fever vaccines (Annex 5)**. In.; 2013.
11. Vannice K, Wilder-Smith A, Hombach J: **Fractional-Dose Yellow Fever Vaccination - Advancing the Evidence Base**. *N Engl J Med* 2018.
12. PATH: **Yellow Fever vaccination: The potential of dose-sparing to increase vaccine supply and availability**. In.; 2013.
13. Ahuka-Mundeke S, Casey RM, Harris JB, Dixon MG, Nsele PM, Kizito GM, Umutesi G, Laven J, Paluku G, Gueye AS *et al*: **Immunogenicity of Fractional-Dose Vaccine during a Yellow Fever Outbreak - Preliminary Report**. *N Engl J Med* 2018.
14. Lopes Ode S, Guimaraes SS, de Carvalho R: **Studies on yellow fever vaccine. III--Dose response in volunteers**. *J Biol Stand* 1988, **16**(2):77-82.
15. Roukens AH, Vossen AC, Bredenbeek PJ, van Dissel JT, Visser LG: **Intradermally administered yellow fever vaccine at reduced dose induces a protective immune**

response: a randomized controlled non-inferiority trial. *PLoS One* 2008, **3**(4):e1993.
16. Martins RM, Maia Mde L, Farias RH, Camacho LA, Freire MS, Galler R, Yamamura AM, Almeida LF, Lima SM, Nogueira RM *et al*: **17DD yellow fever vaccine: a double blind, randomized clinical trial of immunogenicity and safety on a dose-response study**. *Hum Vaccin Immunother* 2013, **9**(4):879-888.
17. de Menezes Martins R, Maia MLS, de Lima SMB, de Noronha TG, Xavier JR, Camacho LAB, de Albuquerque EM, Farias RHG, da Matta de Castro T, Homma A *et al*: **Duration of post-vaccination immunity to yellow fever in volunteers eight years after a dose-response study**. *Vaccine* 2018, **36**(28):4112-4117.
18. Monath TP: **Review of the risks and benefits of yellow fever vaccination including some new analyses**. *Expert Rev Vaccines* 2012, **11**(4):427-448.
19. Fox JP, Penna HA: **Behavior of 17D yellow fever virus in rhesus monkeys: relation to substrain, dose and neural or extraneural inoculation.** *Am J Epidemiol* 1943, **38**(2):152-172.
20. Roukens AH, Soonawala D, Joosten SA, de Visser AW, Jiang X, Dirksen K, de Gruijter M, van Dissel JT, Bredenbeek PJ, Visser LG: **Elderly subjects have a delayed antibody response and prolonged viraemia following yellow fever vaccination: a prospective controlled cohort study**. *PLoS One* 2011, **6**(12):e27753.

DocuSign Envelope ID: 6B9F30A5-D2C8-45EA-9227-845DBD009D50

NON- INFERIORITY FRACTIONAL-DOSES TRIAL FOR YELLOW FEVER VACCINE

1. GENERAL INFORMATION

**Protocol Number:**	**SERU3797**
**Trial Registration Number:**	**NCT04059471**
**Investigational Product(s):**	Yellow Fever Vaccine
**Funder:**	European & Developing Countries Clinical Trials Partnership (EDCTP)
**Tel:**	+31 70 344 0880
**Email:**	info@edctp.org
**Sponsor:**	University of Oxford, UK
**Tel:**	Tel: +254(0)709983549
**Email:**	pbejon@kemri-wellcome.org
**Drug/Product Manufacturer:**	Institut Pasteur de Dakar, Sénégal

Confidentiality Statement

The information contained herein is privileged or confidential and may not be disclosed unless such disclosure is required by applicable laws or regulations. In any event, persons to whom the information is disclosed must be informed that the information is privileged or confidential and may not be further disclosed by them. These restrictions on disclosure will apply equally to all future information supplied to you, which is indicated as privileged or confidential. This confidentiality statement also applies to data generated during the course of the study.

PRINCIPAL INVESTIGATOR'S APPROVAL OF THE PROTOCOL

The undersigned acknowledge possession of and have read the protocol "Non- Inferiority Fractional-Doses Trial for Yellow Fever Vaccine". Having fully considered all the information available, the undersigned consider that it is ethically justifiable to give fractional does of Yellow Fever Vaccines to selected participants according to the agreed protocol. I understand that all information concerning fractional doses supplied to me in connection with this study is confidential information. This includes the Clinical Trial Protocol, Case Report Forms and any other preclinical and clinical data provided. I understand that no data are to be made public or published without prior knowledge and written approval by the University of Oxford.

By my signature below, I hereby attest that I have read, understood and agreed to abide by all the conditions, instructions and restrictions contained in this Protocol and in accordance with the most recent Declaration of Helsinki, Good Clinical Practice and all applicable regulatory requirements. I acknowledge that the Sponsor of the study has the right to discontinue the study at any time.

Principal Investigator: Prof. George Warimwe **Signature:** DocuSigned by: George Warimwe 74835B34EE13491...

Date: 17-Jan-2023

GLOSSARY OF TERMS AND ABBREVIATIONS:

AE	Adverse Event
CI	Confidence Interval
CRF	Case Report Form
DRC	Democratic Republic of Congo
DSMB	Data and Safety Monitoring Board
EPI	Expanded Programme on Immunization
GCP	Good Clinical Practice
GDPR	General Data Protection Regulation
GMT	Geometric Mean $PRNT_{50}$ Titre
GMFI	Geometric Mean Fold Increase
HIV	Human Immunodeficiency Virus
IB	Investigator Brochure
ICF	Informed Consent Form
ICH	International Conference on Harmonization
ID	Intradermal
IgG	Immunoglobulin G
IP	Institut Pasteur
IRB	Institutional Review Board
ISF	Investigator site file
ITT	Intention to treat
IU	International Units
LOQ	Limit of Quantification
MLD50	Mouse Lethal Dose 50
MSF	Médecins Sans Frontières, Doctors without borders
MUST-REC	Mbarara University of Science and Technology – Research and Ethics Committee
NRA	National regulatory authority
NDA	National Drug Authority
OXTREC	Oxford Tropical Research Ethics Committee
PFU	Plaque Forming Units
PI	Principal Investigator

DocuSign Envelope ID: 6B9F30A5-D2C8-45EA-9227-845DBD009D50

PIS	Patient information sheet
PP	Per protocol
PPB	Pharmacy and Poisons Board
PRNT	Plaque-Reduction Neutralization Test
RAP	Report and Analysis Plan
RKI	Robert Koch Institute
SAE	Serious Adverse Event
SAGE	Strategic Advisory Group of Experts on Immunization
SAP	Statistical Analysis Plan
SC	Subcutaneous
SSL	Secure Sockets Layer
SOP	Standard operating procedure
SUSAR	Suspected unexpected serious adverse reaction
UNCST	Uganda National Council of Science and Technology
UNICEF	United Nations International Children’s Emergency Fund
UVRI	Uganda Virus Research Institute
UNHRO	Uganda National Health Research Organization
WHO	World Health Organization
YEL-AND	Vaccine-associated neurological diseases
YEL-AVD	Vaccine-associated viscerotropic disease
YF	Yellow Fever

Table of Contents

1. GENERAL INFORMATION ... 1
2. LAY SUMMARY ... 6
3. LIST OF INVESTIGATORS ... 7
4. ABSTRACT ... 8
5. INTRODUCTION ... 9
6. NAME AND DESCRIPTION OF THE INVESTIGATIONAL PRODUCT ... 13
7. TRIAL OBJECTIVES AND PURPOSE ... 13
8. TRIAL DESIGN ... 14
9. SELECTION AND WITHDRAWAL OF STUDY PARTICIPANTS ... 20
10. TREATMENT OF STUDY PARTICIPANTS ... 22
11. LAB PROCEDURES ... 24
12. ASSESSMENT OF SAFETY ... 26
13. STATISTICS ... 31
14. DIRECT ACCESS TO SOURCE DATA/DOCUMENTS ... 34
15. QUALITY CONTROL AND QUALITY ASSURANCE ... 34
16. INTELLECTUAL PROPERTY ... 35
17. TIME FRAME/DURATION OF THE TRIAL ... 35
18. ETHICS ... 36
19. ARCHIVING AND RECORD RETENTION ... 39
20. FINANCING AND INSURANCE ... 40
21. TRIAL MANAGEMENT ... 41
22. REPORTING, DISSEMINATION AND NOTIFICATION OF RESULTS ... 42
23. APPENDICES ... 42

2. LAY SUMMARY

Formal Title: Non- inferiority fractional-doses trial for yellow fever vaccine

Lay Title: A trial to determine the safety and immune response elicited by the reduced doses of yellow fever vaccine in comparison to full dose of the vaccine in Kenyan and Ugandan adults and children

What is the problem/background?
Yellow fever (YF) is a disease caused by a mosquito-borne flavivirus that is endemic in sub-Saharan Africa and tropical South America. YF virus infection can cause mild or severe illness, leading to jaundice, kidney failure, bleeding and death. The YF vaccine is shown to be very effective for disease control, including prevention of YF outbreaks. However insufficient vaccine is produced for routine use, and whilst a YF vaccine stockpile is reserved for outbreak control, this is frequently depleted. Measures to increase the global supply of YF vaccine are urgently needed.

What questions are we trying to answer?
The World Health Organization (WHO) has recommended consideration of using fractions of standard YF vaccine dose to be able to vaccinate more individuals with a given quantity of vaccine in outbreak situations when there are insufficient doses to vaccinate the population at risk. However, the actual minimal dose of YF vaccine needed to elicit an immune response has not been determined. In this study, we will assess whether the immune response and adverse events occurring after vaccination of adults and children with the standard full dose of YF vaccine are comparable to those observed after vaccination with one of three lower doses of vaccine aiming to establish a minimal dose. Further, to support the implementation of a future YF control strategy using lower doses of vaccine, we will evaluate the views and perceptions of different stakeholders involved in national or international vaccine policy regarding the use of lower doses of the YF vaccine.

Where is the study taking place, how many people does it involve and how are they selected?
The vaccination study will take place in Kilifi, Kenya and Mbarara, Uganda among healthy adults and children who have previously not had the YF vaccine and/or YF infection and have no contraindications for vaccination. In total, 480 adults (240 at each site) and 420 children aged 9 to 12 months (210 at each site) will be included. To evaluate views and perceptions on the use of fractional YF vaccine doses we will approach and conduct discussions and interviews with various stakeholders in national vaccine policy in Kenya, Uganda, Senegal and other international institutions.

What does the study involve for those who are in it?
Participants who have previously not had the YF vaccine and/or YF infection will be screened for any significant health problems. Those found eligible to participate will then be randomized to receive a single dose of the standard full dose of YF vaccine (group 1) or any of the three lower doses (groups 2 to 4). Participants will then be required to attend follow up visits to have blood taken for tests to measure immune response to vaccination, the level YF vaccine in the body, and to be

asked about any side effects. Vaccine policy stakeholders included in the study will be requested to participate in interviews at their convenience.

What are the benefits and risks/costs of the study for those involved?
The risks relate to the possibility of developing an allergic or other reaction upon administration of the YF vaccine. Serious adverse reactions to the vaccine are, however, rare. There are no immediate health benefits to individuals participating in this study other than information about their health. If there was a YF outbreak in the future, participants getting the full YF vaccine dose would be expected to be protected and there is a chance that participants who receive the lower dose would be protected against infection. We are not able to issue YF vaccination certificates to participants at the point of vaccination. If the participant travels to endemic areas and needs a YF vaccination certificate for proof of vaccination, they will be advised to be revaccinated at an authorized health facility in Kenya or Uganda, respectively. However, on completion of the study we will offer participants the possibility to receive a YF vaccination certificate. No safety concerns are reported with multiple doses of the vaccine.
The vaccine policy stakeholders participating in the study will not be at any risk associated with the vaccine trial. Any costs they incur during the process of the study will be reimbursed as out of pocket expenses.

How will the study benefit society?
If any of the lower YF vaccine doses safely elicits immune response that are comparable to the full vaccine dose, then in effect this finding could have an impact on the number of doses that are produced, and substantially increase the number of doses that can be given based on the world's currently available vaccine stock, and thereby enhance our ability to prevent and control YF outbreaks. The results from the interviews and discussions with vaccine policy stakeholders will support the development of a strategy to implement the use of lower YF vaccine doses for disease control.

When does the study start and finish?
The study will start upon receipt of ethical approval and will be completed 40 months later.

3. LIST OF INVESTIGATORS

KEMRI CGMRC Investigators: George Warimwe (PI), Philip Bejon,Sassy Molyneux, Mainga Hamaluba, John Gitonga, Marianne Munene,

Epicentre, Mbarara Investigators: Maria Namulwana, Juliet Mwanga, Dan Nyehangane, Edgar Mulogo

Collaborators:

- **IP Dakar, Senegal**: Amadou Sall, Antoine Diatta
- **UNHRO, Uganda**: Julius Lutwama, John Kayiwa, Pontiano Kaleebu
- **Epicentre, France**: Rebecca Grais, Aitana Juan-Giner

4. ABSTRACT

In July 2016, the demand for yellow fever (YF) vaccines in response to the large urban outbreaks occurring concurrently and the risk of further spread through Africa and to Asia was larger than the available global supply. In this situation, the World Health Organization (WHO) developed recommendations for the use of fractional doses of YF vaccine as a dose-sparing strategy. These recommendations were based on data from a limited number of clinical trials, none of which had been conducted in Africa. Additional studies were initiated to assess the applicability of fractional doses to all four WHO-prequalified YF vaccines with respect to vaccine immunogenicity in adults and children in Africa, including HIV positive adults. One such study, comparing full standard dose to 1/5th of standard dose of all four WHO-prequalified YF vaccines in adults (Clinicaltrials.gov number: NCT02991495), is currently ongoing at KEMRI CGMRC (see SERU protocol 3452) and Epicentre, Mbarara (UNCST HS 2237) and is designed to answer questions on the use of current stock of YF vaccines with a potency as close as possible to each manufacturers' minimum release. Data from this trial will inform a WHO recommendation on using 1/5th of the current standard dose of vaccine for outbreak control. However, since many vials will contain excess YF vaccine such that 1/5th of a vial is likely to be substantially above the current minimum potency requirements, these data may not be scientifically explanatory regarding the minimum dose required for preventive use.

Here, in this new complementary study, we aim to determine the lowest YF vaccine dose that is non-inferior to the current standard full dose among populations in sub-Saharan Africa. The study will be conducted in Kenya (KEMRI CGMRC, Kilifi) and Uganda (Epicentre, Mbarara) with trial participants recruited at both sites, using vaccine from one WHO-prequalified manufacturer (Institut Pasteur de Dakar, Senegal). Our primary aim is to compare the immunogenicity of full standard dose of vaccine with three lower doses in adults, using seroconversion at 28 days post-vaccination, measured by plaque reduction neutralization assay ($PRNT_{50}$), as the endpoint. Adult participants (n=480) will be randomized for vaccination with full standard dose or with approximately 1000, 500 or 250 IU of vaccine (i.e. 4 arms, 1:1:1:1 allocation ratio). Safety and immunogenicity results will then be reviewed by the study DSMB, and the lowest non-inferior dose selected for assessment in a sub-study in children (n=420) in comparison to full standard dose (i.e. 2 arms, 1:1 allocation ratio). Secondary objectives will include immunogenicity at 10 days, 1 year and 2 years post-vaccination, assessment of the influence of cross-reactive antibodies to other flaviviruses on vaccine immunogenicity, assessment of T and B cell immune responses, assessment of virological and immunological kinetics and occurrence of serious adverse events (SAE). In addition, we will assess the range of views and perceptions of key stakeholders in vaccine policy and implementation on lower vaccine dose usage during YF epidemics and routine use. We expect the data from this qualitative study to inform the implementation of policies relating to low-dose vaccine usage for the control of YF and other diseases (e.g. pneumococcal vaccines) where such strategies are in consideration.

5. INTRODUCTION

5.1. Background Information

Yellow fever (YF) is a disease caused by a mosquito-borne flavivirus that is endemic in sub-Saharan Africa and tropical South America [1]. Ninety percent of YF cases are in Africa. In these settings, YF virus is transmitted by different mosquito genera in three recognized transmission cycles [2]. A sylvatic cycle involves transmission between forest-dwelling mosquitoes (*Haemagogus spp*) and non-human primate reservoirs, with sporadic incidental transmission to humans (e.g. forest workers). An intermediate cycle, occurring only in Africa, involves mosquito transmission between non-human primates and humans, or human-to-human transmission among humans living or working close to forested areas. An urban cycle involves transmission between humans and urban mosquito vectors, primarily *Aedes aegypti*, and occurs when a viraemic person, infected in the sylvatic or intermediate cycle, introduces YF virus to areas with a large non-immune population and *A. aegypti* vectors resulting in disease outbreaks [2].

Infection with YF virus is characterised by a wide range of manifestations, ranging from subclinical infection with mild and non-specific symptoms, to severe, life-threatening illness with jaundice, renal failure and haemorrhage [3]. The first symptoms appear 3-6 days after an infected mosquito bite and present with an abrupt onset of fever, muscle pain, headache, shivering, loss of appetite, and nausea or vomiting. These symptoms are commonly accompanied by congestion of the conjunctivae and face, and bradycardia despite fever. During the symptomatic period, lasting 3-6 days, the patient is usually viraemic, but this is usually followed by a remission period. Approximately 15% of infected individuals then enter a toxic phase with renewed fever, relative bradycardia, nausea, vomiting, epigastric pain, jaundice, oliguria, and a haemorrhagic diathesis. The symptoms and severity of the disease reflect dysfunction of multiple organ systems, including the liver, kidneys, and cardiovascular system [3]. Whilst there is no specific antiviral treatment for YF, a highly effective vaccine that provides lifelong protective immunity is available [4]. For instance, a recent modelling study using YF case reports between 1987 and 2011 estimated that there were 1.3 million YF infections in Africa in 2013 (95% CI 850,000 – 1.8 million), of which 180,000 were severe (95% CI 51,000 – 380,000) and 78,000 were fatal (95% CI 19,000 – 180,000) [5]. Preventive mass vaccination campaigns were estimated to have averted 450,000 cases (95% CI 340,000 – 560,000) and 28,000 deaths (95% CI 7,200 – 62,000), highlighting the importance of vaccination as disease control tool [5]. However, despite the availability of an effective vaccine, incomplete vaccine coverage, below the WHO-recommended 80% coverage [6], leaves some settings prone to outbreaks as has recently occurred in Angola, Democratic Republic of Congo (DRC) and Brazil [7, 8].

5.2. Yellow Fever vaccines

A highly effective vaccine is available for use against YF in adults and children aged ≥9 months [4]. The vaccine is a freeze-dried preparation of live attenuated YF virus strain 17D, which was developed in 1937 and is produced by four WHO-prequalified manufacturers (Table 1) [9]. A single dose of YF vaccine is considered sufficient to confer life-long protective immunity against all seven known genotypes of wild-type YF virus [4]. Protective levels of YF virus neutralizing antibodies are developed in 80-100% vaccine recipients within 10 days after vaccination, and in 99% within a

DocuSign Envelope ID: 6B9F30A5-D2C8-45EA-9227-845DBD009D50

month [4]. In endemic regions where YF vaccine is provided routinely in Expanded Programme of Immunization (EPI), the vaccine is often co-administered with the EPI Measles vaccine to children between 9 and 12 months. Seroconversion rates appear to be similar regardless of vaccine sub-strain and manufacturer, though vaccine immunogenicity appears to be somewhat lower in children [4]. Two sub-strains of the 17D vaccine are currently used for vaccine production, namely 17D-204 and 17DD. A distinct sub-strain of 17D-204 (i.e. 17D-213) is also in use (Table 1).

The YF vaccine is prepared using various seed strains that were ultimately derived from the 17D strain of YF virus cultured in chicken embryonated eggs [9]. The seed virus is inoculated into 7 to 9 day-old embryonated eggs and after 3-4 days of incubation, infected embryos are aseptically harvested, homogenized and clarified by centrifugation to produce bulk vaccine. Following addition of stabilizers, the diluted bulk vaccine is filled into vials and freeze-dried [9]. As per WHO recommendations, the final vaccine vials/ampoules should contain a minimum of 1000 IU per dose [10]. However, the dose in the final vaccine vials/ampoules usually exceeds the minimum specification substantially to account for potential potency losses during manufacture and the three years shelf-life [10, 11]. The YF vaccine manufacture process is laborious and current capacity to produce increased stock in response to outbreaks is limited [9].

Table 1: Yellow Fever vaccines pre-qualified by WHO (August 2016)

Manufacturer	Commercial Name	Pharmaceutical Form	Sub-strain	Presentation	No. of Doses
Sanofi Pasteur SA	STAMARIL	Lyophilised active component to be reconstituted with excipient diluent before use	17D-204	Vial	10
Bio-Manguinhos/ Fiocruz	Yellow Fever	Lyophilised active component to be reconstituted with excipient diluent before use	17DD	Vial + Ampoule	10
					5
				Two vial set (active + excipient)	50
Institut Pasteur de Dakar	Stabilized Yellow Fever Vaccine	Lyophilised active component to be reconstituted with excipient diluent before use	17D-204	Vial	5
					20
					10
Federal State Unitary Enterprise of Chumakov Institut of Poliomyelitis and Viral Encephalitides	-	Lyophilised active component to be reconstituted with excipient diluent before use	17D-213	Ampoule	2
					5
					10

In 2015, UNICEF estimated the total country forecasts for the period from 2015 to 2017 to be 64 million doses per year, exceeding the availability of vaccines by 42%. UNICEF stated that their

annual procurement is able to cover routine immunization requirements, emergency stockpiles and some limited additional campaigns. In 2000, a global shortage of YF vaccine occurred and this led to the development of the International Coordination Group (ICG) on vaccine provision for YF. The aim of the ICG is to manage and coordinate the provision of emergency vaccine supplies during outbreaks and ensure the best allocation of limited resources(18). In 2000, a stockpile of 2 million doses was reserved for outbreak response. This was increased from 2 to 6 million doses in 2014. However, the stockpile was depleted twice in 2016.

The relatively long-time period required for vaccine production, together with poor epidemiological surveillance and reporting in at-risk countries, makes vaccine need forecasting very difficult. A review conducted by PATH identified a number of factors that limit the production of YF vaccines [12]. The first is related to the small number of YF vaccine manufactures owing, in part, to the absence of a stable demand. Competition for production capacity with other vaccines that are economically more attractive is also an issue. Other limiting factors are related to the production process and include the limited number of Specific Pathogen-Free egg suppliers, gradual depletion of existing seed stocks and a lyophilisation process that can take several days per cycle. There are limited options for developing technological advances that will solve these issues in the near future. The review by PATH concluded that, to address the current insufficient supply of YF vaccine, fractional dosing could be a short to medium term option provided that clinical evidence for non-inferiority, safety and dosages is generated [12].

5.3. Use of fractional doses as a dose-sparing strategy

In July 2016, the demand for YF vaccine in response to the large urban outbreaks occurring concurrently in different parts of Africa and the risk of further spread throughout the continent and to Asia, led WHO to develop recommendations for the use of fractional-dose of YF vaccine as a dose-sparing strategy. Although fractional dosing has recently been used in vaccination campaigns in Kinshasa [13], WHO recommendations were based on a limited number of clinical studies and important data gaps remain. These include the applicability of fractional dosing to all WHO-prequalified vaccines, the persistence of neutralizing antibodies and the performance of fractional doses in young children and in populations in Africa, including those with HIV [11].

Between July 2017 and March 2018, Brazil has had YF outbreaks with reports of over 720 confirmed human cases including over 230 deaths by February 2018. Fractional doses of YF vaccine were used in selected municipalities to respond to the outbreak following a recommendation by WHO and PAHO (http://www.who.int/csr/don/09-march-2018-yellow-fever-brazil/en/).

WHO recommendations on the use of fractional doses are limited to outbreak response when there are insufficient doses to protect the population at risk. These recommendations have been followed in response to large yellow fever epidemics occurring in the Democratic Republic of Congo in 2016 and Brazil in 2017-2018. Fractional doses have not been used in Kenya or Uganda.

In 1988, a study showed that the use of 200 plaque-forming units (PFU) induced seroconversion in 100% of participants [14]. However, the vaccine was based on older formulations, and we cannot becertain how PFU in that study relate to IU [10]. Two more recent trials have examined the immunogenicity and safety of low doses of YF vaccine. One study examined intradermal (ID)

DocuSign Envelope ID: 6B9F30A5-D2C8-45EA-9227-845DBD009D50

administration of 1/5th of the conventional subcutaneous (SC) dose and showed that this was sufficient to achieve seroconversion in 77 adult participants [15]. In a second, more recent study, YF vaccine was administered by SC route in 900 healthy adult males in de-escalating doses. The study concluded that a dose of ≥587IU was as immunogenic as a 50-fold higher dose (27,476IU/dose), and that these immune responses were sustained over an 8-year period [16, 17].

However, fractional vaccine dosing is compounded by the uncertainty surrounding minimum dose requirements. The current WHO recommendation is that YF vaccine potency should contain not less than 1,000 IU per dose [10]. A thermostability test is undertaken to demonstrate consistency of production. This should show that the geometric mean infectious titre, following incubation of the final containers at 37°C for 2 weeks, is at least 1,000 IU per dose and not have decreased by more than 1.0 $\log_{10}$ IU [10]. The minimum vaccine dose for potency was established in the 1930s and 40s based on experience with lots that varied in titre. The original work was based on mouse median lethal doses (MLD) using intracranial injection. MLD was replaced by assays for PFU, and comparisons between MLD and PFU were used to translate dose requirements. These comparisons varied by manufacturer, and the WHO introduced an international standard to allow doses to be expressed in IU [10]. Therefore, despite the apparent high vaccine effectiveness observed since thresholds were determined in the 1940s, there is uncertainty regarding the precision with which the minimum dose requirements are known.

5.4. Safety considerations for fractional doses

Reactions to YF vaccine are generally mild and include headache, myalgia, malaise and asthenia in around 10-30% of participants during the first few days after vaccination [3, 18]. Serious reactions are rare and include hypersensitivity reactions to egg protein, or to the gelatine used by some manufacturers; vaccine-associated neurological diseases (YEL-AND) and vaccine-associated viscerotropic disease (YEL-AVD), which can vary from multi-organ system failure with limited evidence of hepatitis to a fulminant hepatitis resembling wild-type YF [3, 18]. YEL-AVD is rare (1 in 100,000 vaccinees), occurs within 10 days of a first dose of YF vaccine, and is characterized by severe multi-organ failure. Known risk factors include a history of thymus disease (e.g. thymoma or thymectomy) and age ≥60 years. It has been suggested that the association between serious adverse reactions and primary vaccination may be due to the viremia that primary vaccinees experience following vaccination [18]. The viremia is short-lived, with 17D virus and viral RNA being detectable in the first week post-vaccination and disappearing with the development of neutralizing antibodies following vaccination [18]. Monkey studies have suggested an inverse relationship between YF vaccine dose and the magnitude and duration of vaccine viremia [18, 19]. However, whilst more studies are needed to assess the impact of low vaccine doses on viremia, a previous fractional dosing trial in adults in Brazil found no association between viremia levels and vaccine dose [16].

5.5. Justification

The ongoing trial (Clinicaltrials.gov number: NCT02991495- SERU protocol number 3452 and UNCST HS 2237) is designed to answer questions on the use of vaccines from current stock with doses as close as possible to the minimum release of each WHO-prequalified YF vaccine

manufacturer (Table 1). This trial was prompted by the 2016 DRC outbreak and compares the immunogenicity of standard full dose of YF vaccine to the immunogenicity of 1/5th of a current dose using vaccine from all four WHO prequalified manufacturers. Data from the trial will inform WHO recommendations on the use of 1/5th of the standard dose of vaccine to produce an immune response. However, standard doses contain excess YF vaccine such that 1/5th of a dose is likely to be substantially above the current minimum requirements [11]. Thus, these data may not be scientifically explanatory regarding the minimum dose required for further practice. The proposed study aims to influence the WHO recommendation for the minimum release dose specifications based on current data. This will be done through regulatory engagements and different pathways to impact as advised by experts within the study consortium.

The study therefore aims to determine the lowest dose (in IU/dose) that is non-inferior to the standard full dose among populations in sub-Saharan Africa. The data generated in this study will provide information regarding the re-definition of the minimal dose and potency requirements of the vaccine. The study will also provide further confidence in the use of fractional doses of YF vaccine during epidemics. In addition, we will assess the range of views and perceptions of key stakeholders in vaccine policy and implementation on reduced vaccine dose usage during YF epidemics and for routine use.

A dose response study conducted in Brazil found that a dose of 587IU resulted in seroconversion of 97.7% (95% CI=92.4-99.2%) of participants and that doses as low as 158IU/dose were able to elicit protection in a high number of vaccinees (88.5%; 95%CI 81.5-93.6). Moreover, these immune responses were sustained over an 8-year period [16, 17]. Based on these results, the 3 low dose study groups have been defined as 1000IU/dose (based on the current WHO minimum vaccine potency), 500IU/dose and 250IU/dose.

6. NAME AND DESCRIPTION OF THE INVESTIGATIONAL PRODUCT

We will use YF vaccine produced by Institut Pasteur de Dakar, Senegal. IP Dakar is a member of the study consortium and will supply the investigational product. The product will be a freeze-dried preparation of live attenuated YF virus, sub-strain 17D-204. One vial format will be provided. It will contain standard full dose of vaccine for administration in 0.5ml per dose. The standard dose here is defined as the dose produced by the vaccine manufacturer above the minimum release of 1000 IU/dose. A second vial will be prepared from a standard vial to contain vaccine diluted to approximately 1000 IU/dose for administration in 0.5ml per dose. Fractional volumes of this second vial will be administered as per manufacturer's instructions to achieve the 500 IU and 250 IU doses. A single vaccine batch will be used in this study. This will be provided specifically for the study and will be provided with dilution instructions to achieve the doses described.

7. TRIAL OBJECTIVES AND PURPOSE

7.1. Null hypothesis

Vaccine trial: The rate of seroconversion among vaccinees receiving the 1000, 500 or 250 IU dose of vaccine is lower than that in vaccinees receiving the full standard dose by >10%, as measured by plaque reduction neutralization antibody test ($PRNT_{50}$) 28 days post-vaccination.

Assessment of key vaccine policy stakeholder perceptions: This component of the study will be descriptive.

7.2. Primary objective

To determine the lowest dose (1000, 500 and 250 IU/dose) of YF vaccine that is non-inferior to the full standard dose as measured by seroconversion using the $PRNT_{50}$ assay at 28 days post-vaccination in an adult population.

7.3. Secondary objective(s)

- To describe the geometric mean $PRNT_{50}$ titre (GMT) at 10 days, 28 days, 1 year and at 2 years post-vaccination of the different doses of the YF vaccine.
- To describe the change in $PRNT_{50}$ titre (i.e. the geometric mean fold increase (GMFI) as a continuous variable) between baseline and day 28 after vaccination with the different doses of the YF vaccine.
- To map out key stakeholders' priorities and perceptions regarding a change in policy towards the use of fractional doses of YF vaccine and their potential influence on policy process.
- To measure neutralising antibody to other flaviviruses (including dengue, West Nile and zika viruses) and other locally circulating viruses e.g. SARS-CoV-2 and determine the impact of these antibodies on YF vaccine immunogenicity.
- To assess post-vaccination control of viremia by vaccine dose on samples collected at baseline, and on days 2, 3, 4, 5, 6, 7 and 10 after vaccination.
- To determine the change in T and B cell immune responses between baseline and days 10 and 28 post-vaccination.
- To determine the change in serum cytokine and chemokine levels between baseline and days 2, 3, 4, 5, 6, 7, 10 and 28 post-vaccination.
- To assess the occurrence of adverse events (AE) over 28 days after vaccination and serious adverse events throughout the duration of the study.

Once results for the main outcome are obtained, data will be reviewed by the study Data and Safety Monitoring Board (DSMB). The DSMB will then decide if the study should proceed to the second phase where the lowest non-inferior dose, as measured at day 28 in the adult study, will be selected for assessment in children aged 9-12 months co-administered with the EPI Measles vaccine. The aim of this sub-study will be to assess the non-inferiority in seroconversion of the fractional dose compared to the full standard dose (both in co-administration with the EPI measles vaccine) as measured by $PRNT_{50}$ at 28 days post-vaccination. The procedures described in this protocol include both the study on the adult population and the sub-study in children. The follow-up for the study in children, will be however limited to 1 year.

8. TRIAL DESIGN

8.1. Overall Study Design and Plan Description

This will be a parallel group, randomized, controlled, blinded, non-inferiority trial of four different doses of YF vaccine. The study will be conducted at the KEMRI CGMRC in Kilifi, Kenya and at Epicentre in Mbarara, Uganda. Both these sites are already working together in an ongoing study (Clinicaltrials.gov number: NCT02991495).

Adult participants (n=480) will be randomized for vaccination with full standard dose or with 1000, 500 or 250 IU (i.e. 4 arms) with a 1:1:1:1 allocation ratio. Results for the safety and primary outcome of the adult study will then be reviewed by the DSMB, and the lowest non-inferior dose in the adult study selected for assessment in children aged 9 to 12 months (n=420) in comparison to full standard dose (i.e. 2 arms), when co-administered with the EPI Measles vaccine, with a 1:1 allocation ratio. The determination of the non-inferior dose to use in children will be made by the sponsor in discussion with the study DSMB, vaccine manufacturer and relevant stakeholders, and the final decision communicated to the various regulatory authorities as a notification (i.e. SERU, OXTREC and PPB for the Kilifi site, MUST-REC, UNCST and NDA for the Mbarara site).

Adult vaccinees will be followed up for 2 years, and children for 1 year. There will be no gradual age de-escalation on the basis that there are few safety concerns with the full dose of YF vaccines, having been used in millions of children worldwide. The study procedures will be similar across both sites and these are summarized in Table 2.

Table 2: Study schedule

Procedure	Screening Day -30*	Day 0	Day 2, 3, 4, 5, 6, 7**	Day 10 (+/- 1 day)	Day 28 (+/- 3 days)	Day 365 (+/-14 days)	Day 730 (+/- 28 days)
Informed Consent	X						
HIV Antibody test	X						
Pregnancy test	X	X			X		
Demography	X	X					
Vital signs	X	X		X	X	X	X
History and Physical exam	X			X	X	X	X
Randomization		X					
Vaccination		X					
Blood sample (Adults, Children)		X (10ml, 6ml)	X (4ml, 4ml)	X (10ml, 6ml)	X (10ml, 6ml)	X (10ml, 6ml)	X (10ml, 0ml)
Cumulative blood volume (Adults, Children)		10ml, 6ml	14ml, 10ml	24ml, 16ml	34ml, 22ml	44ml, 28ml	54ml, 28ml
Adverse events and serious Adverse Event			X	X	X	X	X

*Before vaccination. Screening can occur between 0 and 30 days before vaccination. Screening and vaccination can occur on the same day, but participants that are not recruited within 30 days will be re-screened.

**All participants will be randomized to provide one 4ml blood sample at one of 6 time points (days 2, 3, 4, 5, 6, or 7).

8.2. Recruitment

Using existing community engagement strategies developed and successfully implemented for the ongoing trial (Clinicaltrials.gov number: NCT02991495), potential participants will be sensitized, willing volunteers screened, enrolled and vaccinated. During recruitment and consent (see below) we will make it clear that participation will not reliably result in immunity to YF. If participants are subsequently involved in an outbreak or need to travel, unless they have been informed that they received a vaccination that induces immunity in our trial feedback, they should assume that re-vaccination with the full dose YF vaccine is required to be assured of immunity. However, on completion of the study we will offer participants the possibility to receive a YF vaccination certificate. No safety concerns are reported with multiple doses of the vaccine.

8.2.1. Informed Consent and Screening

Before any study specific procedures are undertaken, a member of the study team (clinician, nurse, counselor or field worker), specially trained for the informed consent process, will go through the consent process and participants will be asked to give their individual consent to participate on an informed consent form (ICF) developed specifically for the study. The informed consent will be conducted at the study sites, in private rooms.

The informed consent process will ensure that potential participants have an understanding of the potential risks and benefits of participating in the study, the study procedures (including maintaining confidentiality and anonymity) and study assessment schedule, the use of the blood sample and their right to refuse and/or withdraw from the study at any point without affecting any of the other health services or care they receive, and without having to disclose a reason for their refusal or withdrawal.

Participants will be required to read the full consent or receive a full oral explanation (for illiterate participants) in the language of their choice. Participants will be asked individually by a trained study team member if they understand all parts of the consent and will be given the opportunity to ask any questions and seek clarification. Consent will then be obtained. All informed consent documents will be translated into Kiswahili in Kilifi and Runyankore in Mbarara. For illiterate participants, a witness, selected by the potential participant and not related to the study team, will be requested to be present during the process.

For children (9 to 12 months of age), consent to participate in the study will be requested from parents or guardians. At least one parent, or guardian, will provide written informed consent for her/his child to participate in the study. As with the adult participants, informed consent will be obtained before any studies procedures are undertaken. The child will be withdrawn from the study if the parent or guardian decides it is in the child's best interest.

During the informed consent process, participants will be given the contact details of a designated study staff member and will be advised to contact the study team (by telephone or in person at the study site) if they have a health problem. Participants will be contacted by phone by a member of the study team to be reminded about the participation in the study and the follow-up visits. The study team will take contact details of each participant at the screening visit. A copy of the consent form and participant information sheet (PIS) will also be provided to the participant before concluding the screening visit.

DocuSign Envelope ID: 6B9F30A5-D2C8-45EA-9227-845DBD009D50

This study aims to include healthy individuals who have no contraindications for receiving a YF vaccine. All screening procedures will be similar for all participants. Participants will be screened for eligibility by clinical examination, urine-based pregnancy test and blood tests for HIV serology. During the screening visit, a clinician will assess participants and check the inclusion and exclusion criteria and a rapid HIV test will be done according to Government of Kenya guidelines and the Ministry of Health, Uganda guidelines. This will include the implementation of pre-and post-test counseling as specified in national guidelines. Newly diagnosed HIV persons will be linked to comprehensive care according to the guidelines. Relevant demographic information at baseline will also be collected. Antibodies to YF virus at baseline will be used in analysis but will not be an exclusion criterion.

The screening visit may occur a maximum of 30 days before vaccination. Clinical assessments will be repeated if more than 30 days elapse between screening and proposed enrolment. However, screening and vaccination may occur on the same day.

Participants of the qualitative study evaluating vaccine policy stakeholder views and perceptions will be approached for a request to participate in an interview, scheduled at their convenience. Data collection will only be initiated upon signing an Informed Consent Form.

8.2.2. Enrollment

Participants meeting the inclusion and none of the exclusion criteria above will be enrolled in the study. Vaccination may occur on the day of screening, or deferred to a later day, depending on the timing of screening and vaccination days. Randomization will take place when a volunteer with confirmed eligibility criteria attends for vaccination and completes the enrollment pre-vaccination assessment successfully. This will include an assessment for acute febrile disease, and a urine pregnancy test for female volunteers.

The vaccine will be administered subcutaneously in the deltoid region or upper thigh of either arm or lower limb, avoiding broken skin or injuries. In children it will be administered in the alternative arm that the Measles vaccine is administered. It is recommended, but not required, that the injection be administered into the non-dominant arm. The unblinded team will not participate in further assessments or follow-up visits and will not reveal dose allocations to participants or other trial personnel. All other members of the study team will be blinded until data are locked.

Once the vaccine has been administered, the participant will remain under observation for at least thirty minutes to monitor for any immediate reactions. The necessary equipment will be in place to manage any hypersensitivity reactions. Any immediate local and systemic reactogenicity will be recorded and addressed before they leave the clinic. The participant will then be asked to return to the clinic for the next visit. Vaccine vials will be handled as per manufacturer's instructions, with cold storage verified by a temperature tracker. Vaccine vials that are opened and not used within 6 hours will be discarded.

8.2.3. Scheduled visits

At each scheduled follow-up visit, participants will have a blood sample taken (see Table 2). For adults, a total of 10ml of blood will be drawn at scheduled visits (baseline, 10, 28, 365 and 730 days). For children, a total of 6ml will be taken at scheduled visits (baseline, 10, 28 and 365 days)

The participants will all be randomized to have one additional blood sample collected at either day 2, 3, 4, 5, 6 or 7 for viremia assessment (Table 2). The blood volume for the additional blood sample at one day between days 2 and 7 post-vaccination will be of 4ml. As the transmission of HIV and other blood borne pathogens can occur through contact with contaminated needles, blood and blood products, appropriate blood and body fluid precautions will be employed by all personnel involved in drawing of blood, testing, and handling of all specimens for this study, using standard universal precautions. On visits up to day 28 post-vaccination, participants will be asked retrospectively about adverse events and serious adverse events occurring currently or since the previous visit and these will be documented in the CRF.

8.2.4. Unscheduled visits

Participants will be reminded to contact the study team if they experience symptoms of concern related to the expected vaccine reactions between scheduled study assessments. Any interventions required to treat a disease or condition in an enrolled participant will be allowed. Concomitant administration of other vaccines included in the EPI schedule is accepted. All concomitant interventions will be determined by asking the participant at the scheduled visits and recorded in the appropriate CRF pages.

The study team will provide medical care to participants during the study follow-up period for acute illnesses. The study teams will not become responsible for long-standing chronic conditions that were present before vaccination, or that are unrelated to vaccination. Medical care will be provided within the Kenyan and Ugandan Ministry of Health guidelines, respectively.

Data on adverse events and serious adverse events will be collected as described in later sections below. Female participants becoming pregnant during the follow-up period will be referred for ante-natal visits to health facilities of their choice and pregnancies will be followed-up and outcome recorded if the pregnancy is detected within 28 days of vaccination as described in section below.

8.2.5. Provision of medical care

The study teams will provide medical care to participants during the study follow-up period. For medical care needs that cannot be met by the study team, participants will be provided with access to medical care for any health problem experienced during the follow-up provided free of charge following national protocols during the duration of their study-follow-up. No specific data are available on the risks of pregnancy after yellow fever vaccination. As a precaution, the Centers for Disease Control and Prevention in the USA, recommend that a woman should wait 4 weeks after receiving the vaccine before conceiving [20]. Following this, we will inform, advice and provide access to contraception to women participating in the study for a period of 4 weeks after vaccination. A clinician will discuss individually with each participant the different contraception options and will help her decide the most appropriate method.

Female participants becoming pregnant during the first 28 days following vaccination will be referred for ante-natal visits to a pre-identified referral structure. Pregnancies will be followed-up and outcome documented.

8.2.6. End of treatment visit

To reduce loss to follow-up at the 12-month (adult and children studies) and 24-month visit (adult study only), the study team will contact participants by phone at around 6 months and 18 months after inclusion to remind them about their participation in the study and about the remaining follow-up visit. The 24-month visit will be the final study visit. A blood sample will be taken, and the participants' general health status recorded.

8.2.7. Long-term follow up

Depending on the results at the end of study visit, we may contact participants to enable long-term follow-up (i.e. >1 year for children and >2 years for adults) of immunogenicity for the different vaccine doses. The long-term follow-up will depend on the initial data from this primary study. An amendment to the protocol with details of any planned long-term follow up will be made and submitted to the various regulatory authorities (i.e. SERU, OXTREC and PPB for the Kilifi site, MUST-REC, UNCST and NDA for the Mbarara site) for review and approval.

8.2.8. Qualitative data collection

Qualitative data will be collected from stakeholders to explore the range of perceptions on the use of fractional YF vaccine doses during epidemics in areas where there is vaccine shortage or for routine use. Data will be collected primarily through semi-structured individual interviews (see Table 3). Following informed consent, the participants will be invited for individual interviews, with questions asked aimed at stakeholders' priorities and concerns regarding the use of fractional doses and their influence on different stages of the policy process (agenda setting, policy formulation and implementation). The national and sub-national regulatory bodies will be sampled from Kenya, Uganda and Senegal.

Figure 1: Policy analysis framework and key stakeholders

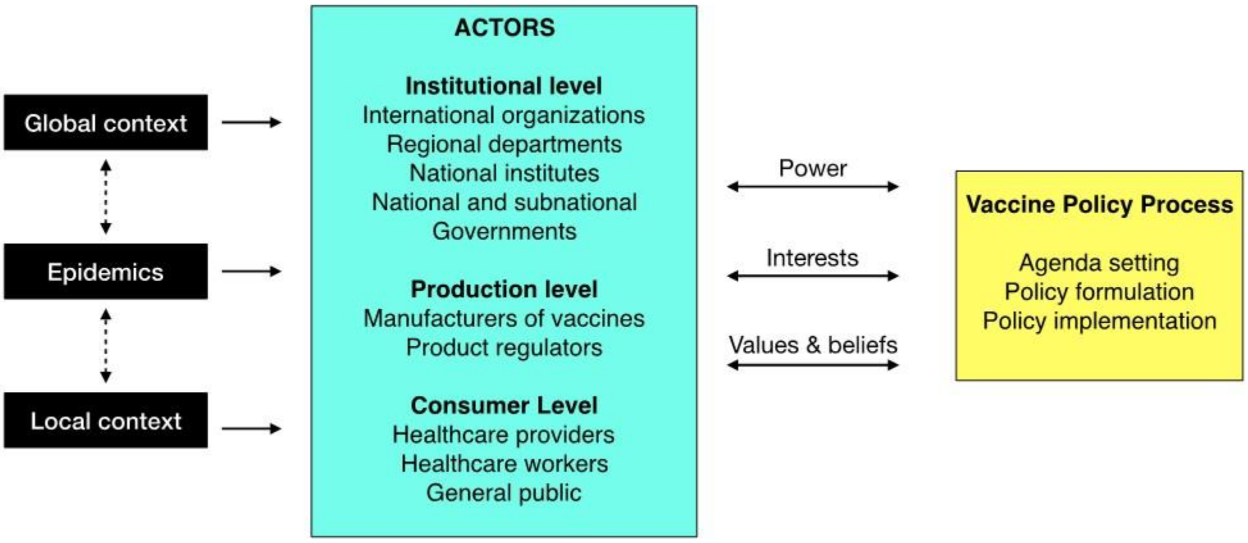

Stakeholders to be sampled in this study

Participant (n)	Affiliation of stakeholder	Method of data collection	Rationale and expertise
**Medical Officer (2)**<br>**Clinical Officer (2)**<br>**Nurse (2)**<br>**Public Health Officer (2)**	Primary Heath Care Providers	FGD, IDI	Implementors of vaccination during epidemics
**County Health Officials (6)**	Implementers of policy	FGD, IDI	Planners of vaccination during epidemics
**PPB (2), NDA (2)**	National Regulatory Authorities	IDI	Regulatory authorities
**Officials (6)**	Vaccine Manufacturers	IDI	YF vaccine manufacturers
**Official (2)**	GAVI, The vaccine Alliance	IDI	Funding of vaccines
**Official (2)**	PATH	IDI	YF strategic demand forecasting
**Official (2)**	SAGE	IDI	Subject experts
**Official (2)**	WHO	IDI	Involved with supply of YF vaccine and interactions with manufacturers, Eliminate Yellow Fever Epidemics (EYE) official
**Researcher/Academic (3)**	Research Institution/University	IDI	Subject experts

*PPB - Pharmacy & Poisons Board, Kenya; NDA - National Drug Authority, Uganda; SAGE - WHO Strategic Advisory Group of Experts on Immunization
IDI - in-depth discussion; FGD - focus group discussion

The framework shown in Figure 1 maps the key stakeholders that are involved and their link to the policy. We will draw our questions and analysis from the stakeholder analysis approach which can be useful to understand actors' perceptions, behavior, intentions, inter-relations and interests in reference to their past, present positions and future potentials. We will conduct about 25 interviews with the different stakeholders. To ensure accuracy of the data and analysis, we will feedback learning to a sub-group of interviewees as a draft report and seek their views on the key findings and interpretations. Depending on initial data and emerging themes, we may sample more stakeholders at each level of the actors.

9. SELECTION AND WITHDRAWAL OF STUDY PARTICIPANTS

9.1. Description of the population to be studied

The main study will recruit healthy adults living in Kilifi and Mbarara who are willing to participate. The sub-study will recruit healthy children in Kilifi and Mbarara whose parents/guardians are willing to consent their participation in the study.

9.2. Inclusion criteria

- Individuals aged ≥18 - <60 years of age.
    - For the sub-study, children aged between 9 to 12 months.
- HIV status

  - HIV negative on serological screening OR
  - HIV positive adults on serological testing, and no symptoms suggestive of current clinical immunosuppression and CD 4 count>200 (for adults) and CD4% > 25% (for children) within the last 6 months.
- Ability to provide informed consent to participate in the study

9.3. Exclusion criteria

- Known contraindications to YF vaccination such as allergies to egg protein and chicken products or any component of the vaccine (including gelatin, eggs, eggs products or chicken products), immunodeficiency, known thymus disorder, such as thymoma and myasthenia gravis
- Using corticosteroids or other immunosuppressive therapy
- Thymus disorder, such as thymoma and myasthenia gravis
- Acute febrile disease on the day of vaccination with temperature >37.5 degrees Celsius is a temporal contraindication.
- Previous YF vaccination
- Previous YF infection as determined from history
- Pregnancy (as determined by a urine test on the proposed day of vaccination) and lactating women
- Planning to migrate out of the study areas before the end of the study follow-up
- Planning to travel to a country requiring YF vaccination certificate within the first year after vaccination.
- Any condition or criteria, including acute or chronic clinically significant abnormality that in the opinion of the investigator might compromise the wellbeing of the volunteer or interfere with the outcome of the study.

9.4. Withdrawal criteria

In accordance with the principles of the current revision of the Declaration of Helsinki, a participant has the right to withdraw from the study at any time and for any reason and is not obliged to give his or her reasons for doing so. In addition, the participant may be withdrawn for any of the following reasons:

- Participant non-compliance with study requirements (for example follow-up visits) despite reminders and attempts to make contact
- Participant moves out of the study area and cannot be traced
- A SAE, which requires discontinuation of the study involvement or results in inability to continue to comply with study procedures

9.5. Managing withdrawals

The study team will continue to follow up all vaccine recipients, with their agreement, until the end of the study wherever possible. The reason for withdrawal will be recorded in the Case Report Form (CRF) if given. If withdrawal is due to a SAE, appropriate follow-up visits or medical care will be arranged, with the agreement of the participant, until the event has resolved, stabilized or a non-study related causality has been established. Following Good Clinical Practice (GCP) guidelines, data on participants who specifically withdraw their consent for use of their data will not be included in the data analysis. However, participants who withdraw from follow up without withdrawing consent for use of their data will be included.

9.6. Replacing withdrawn participants

Participants that withdraw from the study or withdraw their consent will not be replaced.

10. TREATMENT OF STUDY PARTICIPANTS

10.1. Treatments

The investigational product will be one of the four doses of the YF vaccine.

10.2. Identity of Investigational Product

We will use YF vaccine produced by Institut Pasteur de Dakar, Senegal. The product will be a freeze-dried preparation of live attenuated YF virus, sub-strain 17D-204. One vial format will be provided. It will contain standard full dose of vaccine for administration in 0.5ml per dose. A second vial will be prepared from a standard vial to contain vaccine diluted to approximately 1000 IU/dose for administration in 0.5ml per dose. Fractional volumes of this second vial will be administered as per manufacturer's instructions to achieve the 500 IU and 250 IU doses. A single vaccine batch will be used in this study

10.3. Storage

YF vaccine will be stored and transported at a temperature ranging between +2°C and +8°C as per manufacturers' instructions. The vaccine is sensitive to light and needs to be protected from sunlight. The vaccine vials and diluent will be transported together. Vaccine will be reconstituted solely with the diluent provided by the manufacturer. The diluent provided with the vaccine needs to be between +2°C and +8°C at the time of reconstitution. For this, diluents need to be placed in the refrigerator at least one day (24h) before its use. Reconstituted YF vaccine is heat labile hence vials will be discarded 6 hours after reconstitution. The specific manufacturer storage and reconstitution instructions indicated in the product insert will be followed.

Temperatures in the cold chain will be monitored regularly through the day to avoid any deviations. Where temperature excursions occur, they will be resolved with the sponsor following the manufacturer's recommendations. The reconstitution and discard hours will be recorded in the vaccine administration form. The YF vaccines are attached with a vaccine vial monitor type 14 (VVM14), which means the vaccines can withstand cumulative exposure to 37°C for up to a period of 14 days and still retain potency.

DocuSign Envelope ID: 6B9F30A5-D2C8-45EA-9227-845DBD009D50

10.4. Dose selection

Standard dose of vaccine will be administered in 0.5ml per dose. For the lower doses, a second vial will be prepared from a standard vial, by the manufacturer, to contain vaccine diluted to approximately 1000 IU/dose for administration in 0.5ml per dose. Fractional volumes of this second vial will be administered as per manufacturer's instructions to achieve the 500 IU and 250 IU doses. A single vaccine batch will be used in this study.

10.5. Timing of Doses

The vaccine will be administered once during the vaccination visit.

10.6. Randomization and Blinding

Each participant will be randomized to receive one of the four vaccine doses. The allocation will be to one of the four treatment arms per a computer-generated randomization schedule. Randomization will be done by randomization booklets with concealed scratch cards, allocated in order of recruitment and opened on the day of vaccination. These will be prepared by a person outside the study. To minimize vaccine wastage, randomization will be done in block sizes that match with clinic visit days. Allocations will be concealed until a member of the unblinded study team scratches the randomization booklet to reveal the participants' randomization arm.

All participants will receive the same information regarding adverse events. Participants will not be informed of the allocated vaccine. The vaccine will be prepared outside of the view of the participants.

The children will be randomized to either full dose or the lowest non-inferior dose of the vaccine. The vaccine dose to be used will be selected based on the safety and immunogenicity results at day 28 post-vaccination from the adult study. Similar randomization procedures as the adult study will be used. The children will be vaccinated in collaboration with a Ministry of Health EPI clinic for the EPI Measles vaccine.

10.7. Dispensing Procedures

The vaccinating nurse and pharmacist will be unblinded to the allocation of the vaccine doses. They shall not disclose the allocations to any of the team members and they will not participate in the follow up the participants post vaccination. The rest of the trial team will be blinded throughout the study and until data are locked.

Study vaccines will be labelled specifically for the study. Vaccines will be reconstituted once the first participant of the day has been randomized to a specific vaccine dose. The pharmacist together with the vaccinating nurse will be responsible for preparation and administration of the vaccines as randomised. They will be responsible for the accompanying documentation.

10.8. Dose Administration

The vaccine dose to be administered will be guided by the randomization process. The doses to be evaluated will be the full standard dose, 1000 IU, 500 IU and 250 IU. After reconstitution according to the manufacturer's instructions the full dose and the 1000 IU will each be in a volume of 0.5ml.

For the 500 IU and 250 IU doses, fractional volumes as per instructions from the manufacturer will be administered. Vaccine will be administered subcutaneously. As multi-dose vials will be used, the aliquot number (1,2,3, etc.) will be documented on the CRF. The YF vaccine contains no preservative; hence the repeated manipulation for vaccine reconstitution and withdrawal could lead to a greater risk contamination of the vial contents. The pharmacist and vaccinating nurse will prepare the vaccine using aseptic techniques.

10.9. Unblinding

Unblinding will be done at the end of the trial. All participants will be encouraged to have a YF vaccine regardless of the randomization arm if required for travel or in case of an epidemic. Hence unblinding will not be necessary unless as per DSMB request for SAE review.

10.10. Prior/Concomitant Therapy

Concomitant therapy will be documented. Previous YF vaccine or planned YF uptake in the next 24 months will be noted for exclusion.

11. LAB PROCEDURES

11.1. Screening assessments

HIV serology will be conducted using rapid diagnostic tests following the Governments of Kenya and Uganda guidelines. Pregnancy will be determined using a urine test kit in accordance with manufacturer's instructions.

11.2. Research assessments

Venous blood samples (10mls for adults, 6mls for children) will be collected at baseline, 10 days, 28 days, 1 year and 2 years (adults study only) post-vaccination as outlined in Table 2. Additionally, at the inclusion visit 2 ml of blood will be collected to perform the HIV test. The use of venous blood for HIV testing will avoid taking capillary blood from a finger. For difficult to bleed participants, capillary blood may be collected instead. For the children's study, study staff skilled in taking blood from children will be responsible for the phlebotomy. The 6ml blood sample volume (or 8ml at the inclusion visit) for children is lower than the limit specified by existing pediatric guidelines, ranging from 1–5% of total blood volume within 24 hours and up to 10% of total blood volume over 8 weeks (22).

Serum will be isolated from blood used for virus neutralization assays ($PRNT_{50}$). PRNT is considered the most sensitive and specific test for quantification of neutralizing antibodies and is the reference method for assessing immune response after vaccination. Viral RNA will be isolated from serum for detection YF vaccine virus by qRT-PCR [21] on days 0, 2, 3, 4, 5, 6, 7 and 10 (Table 2). For this purpose, all individuals will be sampled on days 0 and 10 and randomized to provide an additional sample on either day 2, 3, 4, 5, 6 or 7. This sparse sampling approach will allow detection and modeling of YF vaccine virus levels in blood, by study arm, whilst minimizing the number of samples taken per individual. In this study we will also characterise the cellular (T and B cell) immune responses to YF vaccination in both adults and children. Whilst these responses have been

DocuSign Envelope ID: 6B9F30A5-D2C8-45EA-9227-845DBD009D50

previously characterised, there is very little data from populations in Africa, including knowledge gaps on how the cellular immune kinetics change with vaccine dose (full vs. lower doses) and age [22]. We will therefore isolate peripheral blood mononuclear cells (PBMCs) from the blood samples collected at baseline, day 10 and day 28 post-vaccination and use these for assessment of cellular immune responses by flow cytometry. In addition, to complement the cellular immune assessments by flow cytometry, we will measure the chemokine and cytokine response to vaccination in serum samples collected at baseline and on days 2, 3, 4, 5, 6, 7, and 10 using a multiplex immunoassay system.

Processing of blood will be done within 24 hours of sample collection at the KEMRI CGMRC immunology lab and at the Epicentre laboratory in Mbarara. Samples will be stored at the KEMRI CGMRC and Epicentre Mbarara repositories. The $PRNT_{50}$ assay for yellow fever will be conducted at the WHO approved reference laboratory at Institut Pasteur de Dakar in Senegal using standard techniques. The lab is performing the $PRNT_{50}$ assays for the ongoing trial (Clinicaltrials.gov number: NCT02991495) thus allowing comparability of the data generated in this study with that from the ongoing trial using a single $PRNT_{50}$ assay.

Institut Pasteur de Dakar in Senegal will also be providing the vaccine. However, there will be no conflict of interest, as the vaccine production unit will supply the vaccine while the lab unit will conduct the $PRNT_{50}$ assays, which are two separate entities. Moreover, the lab personnel will be blinded to the vaccine dose allocations.

High seroprevalence of antibodies to flaviviruses (e.g. dengue virus) has been reported in East Africa [23, 24] raising the question whether presence of antibodies to other flaviviruses at the time of immunization has an impact of YF vaccine immunogenicity. Baseline samples will therefore be evaluated for presence of antibodies to other flaviviruses (including dengue, West Nile and Zika virus) and their association with YF vaccine immunogenicity assessed. The influence of exposure to these related flaviviruses and other locally circulating viruses (e.g. SARS-CoV-2, chikungunya and others) on the magnitude and longevity of the immune response will also be assessed.

Assessment of the cellular immune response to YF (using PBMCs and measurement of cytokines in serum) and neutralising antibodies against other flaviviruses and other locally circulating viruses will be done at KEMRI CGMRC. YF vaccine virus detection by qRT-PCR will be done in collaboration with the WHO approved reference laboratory at Uganda Virus Reseach Institute (UVRI) in Entebbe, Uganda using a standardised assay. These immunological and molecular assessments will be undertaken alongside training of research assistants at both trial sites. Permission will be sought from SERU and Ugandan ethical committees to allow sample shipment to collaborating research scientists at the WHO approved reference laboratories at Institut Pasteur de Dakar in Senegal, UVRI in Entebbe, Uganda and KEMRI CGMRC, Kenya. All specimens will be shipped in accordance with IATA specimen shipping regulations for infectious materials. Results from the $PRNT_{50}$ and qRT-PCR assays will be provided to KEMRI CGMRC for verification and for data entry into the KEMRI CGMRC database.

Serum samples will also be shipped to the Jenner Institute at the University of Oxford for the purpose of systems serology analysis. This is a specialised suite of functional and biophysical assays, optimised at the Jenner Institute, that enables the collection of population-level functional and biophysical antibody profiles. This analysis will provide a comprehensive description of specific characteristics of

YF vaccine induced humoral immune responses in antibodies via Fc effector function assays and will then link these functions back to biophysical antibody features. There are no studies that have provided a detailed characterisation of YF vaccine induced protective immunity in the absence or presence of neutralising antibodies. As demonstrated in studies evaluating Malaria, HIV and SARS-CoV-2 vaccine candidates or immune responses following infection, apart from neutralisation, antibodies can also engage Fc receptors or the complement system to induce a range of Fc-effector functions which have robustly predicted protection from infection [25-27] The degree to which the magnitude and quality of the systems serology-measured antibodies differ by vaccine arm and the respective study endpoints will be assessed.

12. ASSESSMENT OF SAFETY

12.1. Adverse Events (AEs)

SAEs and AE will be recorded in all participants up to day 28. Passive assessment of SAEs will continue during the 2-year follow-up and will be reported.

Adverse events of special interest will be monitored. These will include:

- Injection-site local reaction occurring within 7 days following vaccine administration

While the following adverse events are rare, they will be monitored during the study.

- Vaccine-associated viscerotropic disease (YEL-AVD)
- Vaccine -associated neurotropic disease (YEL-AND)

12.2. Definitions and monitoring of AEs

AEs and SAEs will be defined in accordance with the International Conference on Harmonization (ICH) Guidelines for Good Clinical Practice (38):

An adverse event is defined as any untoward medical occurrence in a clinical trial subject to whom a vaccine has been administered; it does not necessarily have a causal relationship with the vaccine/vaccination. Definitions of AEs and grading, when applicable, to be followed in both study sites will be specified in an SOP. The definition of AE indicates a change in the participant's health status 'since baseline' right before the study vaccine is administered. To assess AE, we'll consider pre-existing conditions and concomitant medication taken prior to vaccination or during the follow-up period.

A serious adverse event is any untoward medical occurrence that:

- Results in death
- Is life threatening: if the participant was at risk of death at the time of the event; it does not refer to an event that hypothetically might have caused death if it were more severe
- Results in persistent or significant disability/incapacity: if the event results in a substantial disruption of the participant's ability to carry out normal life functions. This definition is not intended to include experiences of relatively minor medical significance such as headache,

nausea, vomiting, diarrhea, influenza, injection site reactions and accidental trauma (e.g. sprained ankle)

- Requires in-patient hospitalization or prolongation of existing hospitalization: in general, hospitalization signifies that the participant has been detained (usually involving at least 24h stay) at the hospital or emergency ward for treatment that would not have been appropriate in an outpatient setting
- Is a congenital anomaly/birth defect in the offspring of a study participant
- Is an important medical event that may jeopardize the participant or may require intervention to prevent one of the other outcomes listed above should be considered serious.

Hospitalization for either elective surgery related to a pre-existing condition, which did not increase in severity, or frequency following initiation of the study, or for routine clinical procedures (including hospitalization for "social" reasons) are not considered as SAEs. When in doubt as to whether “hospitalization” occurred, or was necessary, the AE will be considered serious. The definition of a routine clinical procedure is a procedure, which may take place during the study period and should not interfere with the study vaccine administration or any of the on-going protocol specific procedures. If anything, untoward occurs during an elective procedure and satisfies any of the criteria for SAE, this will be documented and reported.

The AEs and SAEs will be assessed for linkage to the vaccination at every contact and this will follow standard operating procedures. All AEs and SAEs will be managed according to the standard care by the study team and/or referred to the most appropriate facilities for specialized care.

12.3. Causality Assessment

Each adverse event will follow a causality assessment. For this, the investigator will determine all contributing factors applicable to each event. These contributing factors will be documented and reported. Every effort will be made by the investigator to explain each AE/SAE and assess its causal relationship to administration of the study vaccine.

In case of concomitant administration of multiple drugs, it will not be possible to determine the causal relationship of SAEs to the individual drugs administered. The investigator will, therefore, assess whether the AE/SAE could be causally related to vaccination rather than to the individual drugs.

The degree of certainty with which an AE/SAE can be attributed to administration of the study vaccine (or alternative causes, e.g. natural history of the underlying diseases, concomitant therapy, etc.) will be determined by how well the event can be understood in terms of one or more of the following:

- Reaction of similar nature having previously been observed after YF vaccination
- The event having often been reported in literature for similar types of vaccines
- The event being temporally associated with vaccination

In line with WHO guidance on investigation of SAE following YF vaccination (42), for SAEs occurring within 30 days after vaccination and following a clinical presentation with generalized

febrile or neurological illness, headache, body pain, nausea, vomiting and/or jaundice or bleeding, will follow a clinical and laboratory assessment. The aim of the clinical and laboratory assessment will be to evaluate if the SAE is linked to the YF vaccination and specifically with neurological/neurotropic disease, viscerotropic disease or severe hypersensitivity reaction.

Where death occurs with similar features and there is no strong evidence for another cause of death, then an autopsy will be advised and, if possible, tissue samples will be collected and analysed.

Laboratory testing will be conducted at the study referral hospital for each study site or its reference laboratory. If needed, samples will be sent to an international laboratory for testing. For all specimens collected, handling and transport will be conducted following standard operating procedures specified by the laboratory.

Samples to be collected, analysis to be conducted and procedures for the storage and handling of samples will be specified in a Standard Operating Procedure (SOP).

The Investigator will assess the causality of all SAEs, using the following question: “Is there a reasonable possibility that the SAE may have been caused by the study vaccine(s)?” After assessment of causality, the investigator will classify the SAE as related or unrelated, as defined below:

- Related: there is suspicion that there is a relationship between vaccine and SAE (without determining the extent of probability); there is a reasonable possibility that the vaccine contributed to the SAE. For example, the SAE follows a known or expected reaction to the vaccine, follows a reasonable temporal sequence from administration of the vaccine and/or is not likely explained by disease or other drugs.
- Unrelated: there is no suspicion that there is a relationship between vaccine and SAE. For instance, the event is more likely explained by another cause, does not follow a reasonable temporal sequence from administration of the vaccine or a causal relationship is considered biologically improbable.
- Unclassified: the SAE can be left unclassified is there is insufficient or contradictory information and data cannot be supplemented or verified.

12.4. Evaluation of Expectedness

A SAE classified as related to the vaccine, will be evaluated for expectedness. A SAE will be considered as unexpected if the nature, seriousness, severity or outcome of the event is not consistent with the known vaccine’s effects as described in the medical literature and described by the manufacturer in the product specifications. Related unexpected SAE will be classified as Suspected Unexpected Serious Adverse Reaction (SUSAR).

10.4 Evaluation of Grade

Each adverse event will be graded using the following general guidelines:

1 = Mild: awareness of AE, but easily tolerated

2 = Moderate: enough discomfort to cause interference with usual activity

3 = Severe: incapacitating with inability to work or do usual activity

4 = Life-Threatening (note this category would imply an SAE which would need additional reporting details).

The term "severe" is often used to describe the intensity (severity) of a specific event (as in mild, moderate, or severe myocardial infarction); the event itself, however, may be of relatively minor medical significance (such as severe headache). This is not the same as "serious", which is based on the outcome or criteria defined under the serious adverse event definition. An event can be considered serious without being severe if it conforms to the seriousness criteria; similarly, severe events that do not conform to the criteria are not necessarily serious. Seriousness (not severity) serves as a guide for defining regulatory reporting obligations.

The "toxicity grading scale for healthy adult and adolescent volunteers enrolled in preventive vaccine clinical trials" will be used. For the paediatric participants, this guideline has been complemented with the division of AIDS (DAIDS) table for grading the severity of adult and paediatric AEs. The definition and grading are defined in the AE/SAE SOP.

12.5. Procedure for recording safety outcomes

Safety information will be actively assessed and recorded by the study doctor during the 10 and 28 days follow-up visits. Participants will be asked for local and systemic events occurring since the previous visit. Moreover, all participants will be instructed to contact the study team immediately, should they experience any signs or symptoms perceived as serious during the study follow-up period. Participants' calls will also be recorded by the study clinical staff receiving the call and will be followed at the site on scheduled or unscheduled visits as appropriate.

Any adverse event, regardless of seriousness, will be recorded on the participant's CRF. When possible, each event will be recorded in terms of diagnosis or syndrome rather than multiple symptoms that are manifestations of the same diagnosis/syndrome. In case the participant reports signs and symptoms, the investigator will obtain a medical diagnosis. If a diagnosis cannot be obtained, each sign or symptom will be recorded as a separate event. Pre-existing conditions or signs and/or symptoms (including any which are not recognized at study entry but are recognized during the study period) present in a participant prior to enrolment will be recorded in the medical history in the participant's CRF.

The diagnosis, date and time of onset, outcome, severity and relationship to vaccination will be established. Details of any treatment or concomitant interventions will be recorded.

All SAEs will be recorded and followed up to resolution, irrespective of severity or whether or not they are considered vaccination-related.

The recording of adverse events in Mbarara will include evaluation of injection-site reactions (pain, induration and erythema) and systemic events (fever, headache, nausea and allergic reactions including rash and urticaria). At the 10 and 28 days post-vaccination visits, participants will be asked for local and systemic events occurring since the previous visit. CRFs for recording adverse events will include questions about specific adverse events and will also allow the recording of unsolicited events.

12.6. Management of AEs and SAEs

Participants with an AE or SAE will be managed with best available care, following national guidelines. When necessary, participants will be referred for medical care to the pre-identified referral structure. Management of any SAE will be recorded in the CRF. For the paediatric participants, a paediatrician will be part of the study team.

12.7. Documenting AEs

Both solicited or unsolicited AEs will be recorded on the participant's CRF. The diagnosis, date and time of onset, outcome, severity and relationship to vaccination will be established. Details of any treatment or concomitant interventions will be recorded.

12.8. Reporting Serious Adverse Events (SAEs) and/or Unexpected AEs

The principal investigator (PI) (or designee) is responsible for reporting and providing updates of SAEs/SUSARs to the Sponsor. The Principal Investigator will report SAEs/SUSARs to the vaccine manufacturer. Adverse events will not be reported but will be recorded in the CRFs and summarised in a 6-monthly safety report. The Principal Investigator will coordinate the safety monitoring and reporting in the study. All SAEs identified up to day 28 will be tabulated and reported in summary form for each population. Expedited reporting of individual SAEs will only be undertaken where they are suspected to be causally linked to vaccination and within 28 days of vaccination but will be compliant with the various local requirements as outlined in Table 4. On completion of vaccination of each population (i.e. adults and children), the Sponsor (or designee) will provide summary reports. This report will summarize the SAE data, diagnosis, causality assessment and outcome. Summary reports will be sent to the DSMB members, investigators, ERCs, PPB, NDA and vaccine manufacturer as per their recommendations. AE's will be objectively monitored using a check list and SOP. The database will be activated to track and generate periodic trends of AE's and SAE's. Systemic (laboratory) AE's will not be actively monitored unless there is a clinical indication. The NDA will be periodically notified about all AE's and SAE's as part of the reporting matrix and linkage to the ministry of health.

Table 4: Expedited reporting matrix for SAEs

Reported to	Reported by	Timeline
Sponsor (or designee)	PI (or designee)	Within one working day of becoming aware of the SAE
KEMRI-SERU (Kenya) and MUST-REC and UNCST (Uganda)	PI (or designee)	Within 24 hours after the P.I learns of event occurrence and hard copies forwarded to IRB within three working days.
OXTREC (UK)	PI (or designee)	Within the timeline established by their procedures.
PPB (Kenya) and NDA (Uganda)	PI (or designee)	Within 7 calendar days of notification.

DSMB	Sponsor (or designee)	Within one working day of the sponsor becoming aware of the vaccine-linked SAE

12.9. Emergency Procedures

During vaccination, staff trained in basic life support will be available in case of adverse reactions around vaccination. An emergency kit will also be available and checked routinely.

12.10. Pregnancy

Pregnancy will be actively assessed at baseline and 28 days post vaccination. Female participants becoming pregnant during the first 28 days following vaccination will be followed until the end of the pregnancy and the outcome will be recorded. This will be reported as an AE as the vaccine is not recommended in pregnant women. The outcome of the pregnancy (live birth, still birth or abortion) will be recorded after birth. Subsequent reports containing follow-up information regarding a pregnancy is not required unless the pregnancy results in a congenital anomaly. The congenital anomaly should be promptly reported as a Serious Adverse Event.

12.11. Procedures for reporting any protocol violation(s)

Protocol violations will be reported to the sponsor, regulatory and ethics committees as specified in their guidelines.

13. STATISTICS

13.1. Determination of sample size

This study will be powered to detect non-inferiority of each lower dose of vaccine (1000 IU, 500 IU, 250 IU) compared to the full standard vaccine dose. Sample size calculations were done using art2bin on Stata version 15.

For the adult study, we assumed a 95% seroconversion rate, 90% power, 2.5% alpha for a one-sided test and a non-inferiority margin of 10%, which gave a sample size of 100 per arm. The 10% non-inferiority margin was chosen in consideration of the public health consequence of a loss of protection but a potential increase in vaccine dosages in a situation where vaccine stocks are insufficient to respond to an outbreak. The sample size was increased by 20% to account for: i) losses to follow up and, ii) unevaluable participants with a positive serological response for YF virus at baseline. Thus, a total sample size of 480 will be required for the four vaccine dose groups (i.e. full dose, 1000 IU, 500 IU and 250 IU; Table 5).

For the study in children, we assumed a 90% seroconversion rate (accounting for lower vaccine immunogenicity reported in children [4]), 90% power, 2.5% alpha for a one-sided test and a non-inferiority margin of 10%, which gave a sample size of 190 per arm. This was increased by 10% to account for 5% losses to follow up and 5% unevaluable participants with a positive serological response for YF virus at baseline. This gave a total sample size of 420 (i.e. 210 in the full dose group and 210 in the lower dose group; Table 5).

Analyses of all other efficacy and safety endpoints are secondary outcomes. Therefore, no significance adjustments will be made for multiple comparisons.

Table 5: Planned participant recruitment numbers in Kilifi and Mbarara

<table>
<tr><th>Population</th><th>Allocation</th><th>Participants in Kilifi (n)</th><th>Participants in Mbarara (n)</th><th>Total per allocation</th></tr>
<tr><td rowspan="4">Adults (N=480)</td><td>Full dose</td><td>60</td><td>60</td><td>120</td></tr>
<tr><td>1000 IU</td><td>60</td><td>60</td><td>120</td></tr>
<tr><td>500 IU</td><td>60</td><td>60</td><td>120</td></tr>
<tr><td>250 IU</td><td>60</td><td>60</td><td>120</td></tr>
<tr><td rowspan="2">Children (N=420)</td><td>Full dose</td><td>105</td><td>105</td><td>210</td></tr>
<tr><td>To be determined</td><td>105</td><td>105</td><td>210</td></tr>
</table>

13.2. Statistical and analytical plans

13.2.1. Data management

A Data Management team will be located at each study site. Data collection will occur at each study site using standardized CRFs transported by designated staff. Data entry clerks will use password-protected computers. Data will be double entered at each study site.

The CRFs in this study will be entered onto an electronic database at each study site. This will be done via secure web interface with data checks used during data entry to ensure data quality. The database will be activated for the study only after successfully passing a formal design and test procedure. Laptops and desktop computers will be used for data entry of paper forms. Management and maintenance of computers will lie with the operational support and data managers at the study site.

13.2.2. Data security, access and backup

The database will be kept in a locked server-room. Only the system administrators have direct access to the server and back-up tapes. A role concept with personal passwords (site investigator, statistician, monitor, administrator etc.) regulates permission for each user to use the system and database, as he/she requires.

All data entered onto the CRFs are transferred to the database using Secure Sockets Layer (SSL) encryption. Each data point has attributes attached to it identifying the user who entered it with the exact time and date. Retrospective alterations of data in the database are recorded in an audit table. Time, table, data field and altered value, and the person are recorded (audit trail). A multi-level back-up will be implemented. Back-ups of the entire system including the database are run internally several times per day and on external tapes once a day. The back-up tapes are stored in a secure location.

13.2.3. Analysis of immunological endpoints

Information for each of the following categories will be presented for the adult study and the children sub-study: number of individuals screened for eligibility; the number and reason of screen failures; the number and percentage of eligible individuals who consent and are randomized; the number and percentage of randomized individuals who receive a vaccine; the number and percentage of vaccinated individuals who complete a day 10 post-vaccination visit; the number and percentage of vaccinated individuals who complete a day 28 visit; the number and percentage of vaccinated individuals who complete a 12 month visit; the number and percentage of vaccinated individuals who complete a 24 month visit; the number and percentage of vaccinated individuals who complete the final analysis period (defined as completing all study activities up to 12 months for the children sub-study, and up 24 months for the adult study); and the number of individuals who discontinue and the reason for discontinuation. Participant demographics and baseline characteristics will be described and compared between vaccine groups using non-parametric tests.

The intention to treat (ITT) population will comprise all randomized participants who received a dose of a study vaccine and that have at least one post-vaccination blood sample. The per protocol (PP) population will include randomized participants who have a blood sample at baseline and 28 days (+/- 3 days) post-vaccination, who are seronegative ($PRNT_{50}$ <1:10) to YF at baseline, and for whom the eligibility criteria were correctly applied. The safety population will include all subjects who received a study vaccine.

The primary analysis will be a pairwise statistical comparison of the rate of seroconversion at day 28 between full dose and each lower dose of vaccine using a non-inferiority test with a margin of non-inferiority of 10% in the PP population. Seroconversion will be defined as a ≥4-fold rise in $PRNT_{50}$ titre between day 0 and day 28 samples. Any $PRNT_{50}$ value reported as below the Limit of Quantification (LOQ) (e.g. <1:10) will be converted to LOQ/2. Thus a 4 rise for a subject who is <1:10 at baseline, is a titre of 20. Each immunogenicity assessment will be a pairwise comparison of the full dose and each lower dose within one study population (i.e. adults or children).

Secondary analyses will include assessment of seroconversion in the ITT population as a whole, on the subset of the ITT population with baseline seropositivity to YF, and in the subset of the PP population with no reported history of flavivirus infection. Geometric mean $PRNT_{50}$ titre (GMT) and GMT fold increase (GMFI) and corresponding 95% confidence intervals (CI) on day 0 and 28 will be calculated. A test of non-inferiority will be performed for the difference in GMT and GMFI between the full dose and each lower dose group. Titres will be graphically represented by reverse cumulative distributions obtained by plotting, for each possible value of the titre (abscissa), the proportion of subjects with a titre greater than this value.

Lower vaccines doses may change the kinetics of antibody response. The assessment of seroconversion rates, GMT, and GMFI 10 days after vaccination in the ITT population will provide important information in the context of low dose vaccine usage in outbreak response. These three immunogenicity outcomes will also be assessed at 1 year and 2 years post-vaccination in the ITT population to confirm a lasting effect of full and low dose vaccination.

Relationships between seroconversion and vaccine immunogenicity ($PRNT_{50}$ GMT and GMFI) will be related to frequencies of specific T and B cell subsets measured by flow cytometry, chemokine

and cytokine levels in sera and neutralising antibody levels to other flaviviruses. Correlations between vaccine viraemia and immunogenicity will be assessed across the different vaccine dose strata. Comparisons of these immune and viraemia kinetics will be made between the adult and children trial participants, whilst accounting for the administered vaccine dose.

Adverse events occurring during the study follow up period will be analysed and compared between groups. This will be a descriptive analysis and will include all AEs up to 28 days post-vaccination, and SAEs that occurred any time during study follow-up.

A detailed statistical analysis plan (SAP) will be provided separately and finalized after the study has started (and before the database lock). This SAP will include all conventions on data, descriptive and statistical analyses to be performed on collected data during the conduct of the study.

13.2.4. Qualitative Data Analysis

Interviews will be conducted in English, audio-taped and transcribed and the subject identifying information omitted. Field notes may also be taken. Analysis will use a thematic framework aiming to identify and categorize the attitudinal, and contextual factors in regard to the policy. The semi-structured in-depth interviews will include topics that influence decision-making in policy. Analysis of qualitative data will follow the framework approach. To ensure that interpretations of quotes are consistent, and that data quality is rigorous and transparent, independent qualitative researchers will code the data; differences between coding will be resolved by group discussion involving other members of the research team. Recurring issues, concepts and patterns will be identified using ground theory and grouped according to thematic areas.

14. DIRECT ACCESS TO SOURCE DATA/DOCUMENTS

The trial team in KEMRI CGMRC and Epicentre, Mbarara will hold source documentation securely. Access will be granted to monitors responsible for quality assurance, for data entry staff and for purposes of medical care. Access will also be granted for audit or inspection by statutory authorities and other relevant persons. Non-study team members will not be granted access. Qualified staff will supervise data collection and entry on a regular basis. Data managers will support onsite data entry teams.

15. QUALITY CONTROL AND QUALITY ASSURANCE

15.1. Monitors and monitoring plan

The study will be conducted in accordance with the current approved protocol, ICH GCP, relevant regulations and standard operating procedures. Monitoring will be performed as per ICH GCP including but not limited to regular visits during the clinical study and a closeout visit. Data will be evaluated for compliance with the protocol and accuracy in relation to source documents. Following written standard operating procedures, the monitors will verify that the clinical study is conducted, and data are generated, documented and reported in compliance with the protocol, GCP and the applicable regulatory requirements. Monitoring will be performed internally at each study site. Moreover, external monitoring visits will be conducted in coordination with the sponsor. Monitors will participate in all key, planned activities, collaborating with implementation of the study. The

monitoring plan is detailed in the Study Monitoring SOP that will be prepared prior to the study. The SOPs will cover instructions for monitoring the main aspects of the implementation of the clinical studies, as follows:

- Study authorizations and approvals and communication with the ethics committees
- Duties and Responsibilities of the Investigator/Institution
- Suitable resources
- Medical care for the participants
- Clinical Study compliance with the agreed protocol
- Laboratory aspects
- Informed consent of participants
- Data management, records and reports
- Sponsor 's Responsibilities
- Responsibilities of the Monitors
- Records of GCP training

16. INTELLECTUAL PROPERTY

The vaccine product assessed in the clinical trial is fully licensed and in routine use, and therefore no product-related IP will arise from this trial. In the unexpected event that other findings lead to IP then KEMRI guidelines will be followed in terms of registering and exploiting the IP. However, any intellectual property rights that arise from the work will be safeguarded as per the KEMRI 2015 IPR guidelines and the Industrial Property Act of 2001, sections 32, 58 and 80. The scientific and intellectual contributions of all persons involved in the research will be appropriately acknowledged in all publications and presentations arising from the work.

17. TIME FRAME/DURATION OF THE TRIAL

The total study duration is 40 months. However, study timelines will be determined after receipt of approval from KEMRI SERU, PPB, OXTREC, MUST-REC, UNCST and NDA. Estimated timelines are shown below.

Activity	Time period following ethical approval
Study staff recruitment, procurement etc.	Month 1 - 6
Community engagement and mobilization	Month 1 - 3
Screening, vaccinations and follow up for adult study	Month 3 - 27

Screening, vaccinations and follow up for children sub-study	Month 12 - 30
Sample processing and laboratory assays ($PRNT_{50}$, qRT-PCR)	Month 3 - 39
Qualitative data collection and analysis	Month 6 - 30
Feedback to study participants, complete data analysis and write up	Month 30 – 40

18. ETHICS

In Kenya, ethical approval will be sought from KEMRI SERU, OXTREC and regulatory approval from PPB for Kilifi. In Mbarara, ethical approval will be sought from MUST-REC and UNCST and regulatory approval from the Uganda NDA. Individual consent will be obtained from each of the study participants.

18.1. Human Subjects

i. "First, do no harm."

The YF vaccine to be used is a licensed product. This, and all other licensed YF vaccines are all derived from the 17D YF virus strain. Tens of millions of YF vaccine doses have been used globally. Adverse reactions to the vaccine are generally mild and include headache, myalgia, malaise and asthenia in around 10-30% of participants during the first few days after vaccination.

Serious reactions are very rare and include hypersensitivity reactions generally associated with egg protein but may also implicate the gelatin used by some manufacturers, vaccine-associated neurological diseases (YEL-AND) and vaccine-associated viscerotropic disease (YEL-AVD), which can vary from multi-organ system failure without much evidence of hepatitis to a fulminant hepatitis resembling wild-type yellow fever. YEL-AVD is a very rare event occurring after a 1st dose of YF vaccine. This occurs within 10 days of vaccination and is characterized by severe multi-organ failure. Known risk factors include a history of thymus disease (e.g. thymoma or thymectomy) and age ≥60 years. We will exclude these groups from vaccination.

Monkey studies have suggested an inverse relationship between YF vaccine dose and the magnitude and duration of vaccine viremia [18, 19]. However, whilst more studies are needed to assess the impact of low vaccine doses on viremia, a previous fractional dosing trial in adults in Brazil found no association between viremia levels and vaccine dose [16]. We will monitor post-vaccination viremia as part of this study.

18.2. Community Considerations

We will use existing community engagement strategies to inform communities about the study where we will involve the local sub-national Health Management teams, local administration and the community members. A community engagement plan specific for the study will be developed between the Community Engagement teams and the investigators. Meetings with institutional heads, chiefs, community leaders and community representatives will be organised to explain the study and its aims, and to discuss concerns.

Research findings will be disseminated to participants and the participating communities upon completion of the study using the appropriate networks and specific meetings. Study participants will be encouraged to take a YF vaccine in the context of an epidemic or travel if this occurs before the unblinding of the study results. Once unblinded, subjects will be notified of the vaccine dose received and the implications for protection in future based on their allocated group. However, the study team will not provide individual-level immunogenicity data to participants, as the assays are not validated for individual-level prediction of protection. Further, the study team will not be able to provide a YF vaccination certificate for travel or as proof of protection to those who have received the full dose. However, on completion of the study we will offer to pay for the cost of re-vaccination at an authorized public health facility for any study participant who would like to receive a YF vaccination certificate. No safety concerns are reported with multiple doses of the vaccine.

18.3. Informed Consent

Meetings will be organized with potential volunteers where investigators will discuss the study objectives, including the risks and benefits of participation, and describe the inconvenience and procedures required for participation in detail. All volunteers will sign and date the informed consent form before any study specific procedures are performed in confidential spaces with a trained member of the study team. All informed consent documents will be translated into Kiswahili and Runyankore.

We will emphasize the following:

- Participation in the study is entirely voluntary.
- Declining to participate involves no penalty or loss of medical benefits.
- A volunteer may withdraw from the study at any time.
- A volunteer is free to ask questions at any time to allow him or her to understand the purpose of the study and the procedures involved.
- There is no direct benefit from participating. The benefits will be realized in the long-term for the community by contributing towards the development of understanding the use of these yellow fever vaccines in different ways.
- Volunteers will be compensated for travel, time and inconvenience of participating.

18.4. Compensation

We will reimburse participants for travel required to attend screening and to attend for vaccinations and follow up. Reimbursement for out of pocket expenses will be done in accordance with standard figures determined by the respective institutional (KEMRI CGMRC and Epicentre) community

engagement teams based on government recommendations on daily wages for skilled and non-skilled labor in Kenya and Uganda.

18.5. Patient Data Protection/Confidentiality

Clinical records will be kept in locked cabinets in the clinical trials facilities at each of the sites. All immunological and qRT-PCR data will be kept in anonymized databases linked by the study number to clinical data.

18.6. Data Sharing

The study will be conducted across two sites (Kilifi and Mbarara) and anonymised data will be entered into a database maintained by KEMRI CGMRC. Individual-level anonymised data will be shared with the vaccine manufacturer and with medical regulators including the WHO for pre-qualification, regulatory and policy purposes. For wider engagement of stakeholders, including the medical community, summary-level statistical analyses will be shared. Information collected or generated during this study may be anonymised for use to support new research and policies for YF vaccine. Any future research using information from this study will need approval from a local or national expert committee to make sure that the interests of participants and their communities are protected.

18.7. Safety

The study team will provide medical care to participants during the study follow-up period for acute illnesses. The study team will not become responsible for long-standing chronic conditions that were present before vaccination, or those that are unrelated to vaccination, and medical care will be provided within the respective (Kenyan or Ugandan) Ministries of Health guidelines.

18.8. DSMB

The trial DSMB will be independent and will meet to review the safety data and reports submitted. After the first phase of the study (adult population), the DSMB will meet to review the data and make a decision on the fractional dose to be used in the second phase (children study). The DSMB may also have ad hoc meetings convened by the DSMB chair and/or the sponsor.

The DSMB will be appointed by the sponsor and be composed of 3 independent members with expertise in clinical trials and vaccinology. The DSMB will be empowered to recommend pausing or stopping the trial to the sponsor, and to request any additional information pertaining to participant safety that is considered necessary.

A first DSMB meeting will be convened before the study commences to review the protocol, roles and responsibilities, operating guidelines and monitoring plan. The remit and functions of the DSMB are described in the DSMB Charter.

18.9. Material Transfer Agreement

A Material Transfer Agreement (MTA), when applicable, will be developed before shipping of samples to the WHO approved reference laboratories at Institut Pasteur, Dakar in Senegal, UVRI in

Entebbe, Uganda, and KEMRI CGMRC, Kenya, The Jenner Institute at the University of Oxford and for shipping of standards from NIBSC and RKI. This will include the following information.

- Identification of the provider and recipient
- Identification of the material and the volume of material
- Definition of the trial and how the material will and will not be used.
- Maintenance of confidentiality of background or supporting data or information, if any.

19. ARCHIVING AND RECORD RETENTION

19.1. Overview

A Data Management team will be located at each study site. Data collection will occur at each study site using standardized CRFs. The data managers will lead and support these activities.

19.2. Investigator site file (ISF)

The investigators will maintain appropriate medical and research records for this study, in compliance with ICH E6 GCP, GDPR, regulatory and institutional requirements for the protection of confidentiality of participants. The principal investigators, co-investigators, and clinical research staff will have access to records. The investigators will permit authorized representatives of the Sponsor, and regulatory agencies to examine (and when required by applicable law, to copy) clinical records for the purposes of quality assurance reviews, audits and evaluation of the study safety and progress.

The ISF will be maintained at the study site containing at least the following documents and information:

- Signed protocol and amendments
- CRFs
- Current informed consent form and all revisions
- Current participant information sheet and all revisions
- Any other written information given to the study team
- Financial aspects of the study
- Insurance statement
- All signed agreements/contracts
- Dated and documented approval of ethics committee and regulatory authorities
- Signed CV's of all investigators and any study personnel (updated regularly as changes occur)
- Monitoring reports
- Relevant communication
- Signed informed consent forms
- Signed, dated, and completed CRFs
- SAE reporting
- Notification by Sponsor of safety information
- Annual reports to ethics committee and regulatory authorities
- Participant screening log

- Participant identification code list (mapping patient onto anonymized study ID)
- Participant enrolment log
- Investigational product accountability
- Authorization/signature sheet
- Clinical study report

19.3. Source documents

All protocol required procedures along with information necessary to report the observations and tests described in this protocol are recorded in CRFs. Any entries captured on CRFs that are derived from source documents e.g. hospital record, will have the source documents included as part of the participant's file. Where source documents for specific entries are not available, this must be explicitly mentioned. Any requested information that is not obtained as specified in the protocol should have an explanation noted on the CRF as to why the required information was not obtained.

CRFs will be completed and signed and authorized in a timely and accurate manner by designated study staff within a month of completion of a visit. All data on the CRFs must be legibly recorded in ink. The investigator or a designated, qualified individual must review all CRFs for accuracy and consistency with any relevant source documentation and sign the CRFs upon completion. Any corrections will be made on the CRFs by striking through the incorrect entry with a single line and entering the correct information adjacent to it. The correction will be initialled and dated by the investigator or a designated, qualified individual. Any corrections made after data entry has begun will be notified to data managers for correction of electronic databases.

19.4. Record keeping and retention

The ISF including a copy of the final completed CRFs, as well as all source documentation is retained by the investigator and one copy will be maintained by the Sponsor, who will ensure that it is stored with other study documents, such as the signed informed consent forms, protocol, the investigator's brochure and any protocol amendments, in a secure place following local regulations.

Data from the interviews will be audio recorded where consent is provided, and field notes written for observations. Audio recordings will be transcribed and destroyed upon transcription. All identifiers will be removed during transcription and replaced with codes. The data will be stored in a secure place following KEMRI-Wellcome Trust Research Programme regulations. The transcriptions will be stored as part of the study database.

The Sponsor will securely store the final study database with all archive tables for at least 10 years. The Sponsor also keeps the central Trial Master File and interim and final reports both in electronic and in hard copy form for at least 10 years. Sites will archive paper CRFs and study files following local laws.

20. FINANCING AND INSURANCE

20.1. Budget

Item	USD	KES	UGX
Personnel, salaries and benefits disbursements	800,000	80,800,000	2,997,600,000
Patient costs, travel, food and/or supplies	200,000	20,200,000	749,400,000
Equipment	16,000	1,616,000	59,952,000
Community engagement	20,000	2,020,000	74,940,000
Supplies			
Laboratory supplies	220,000	22,220,000	824,340,000
Clinical supplies	100,000	10,100,000	374,700,000
Shipping costs	12,000	1,212,000	44,964,000
Travel and accommodation			
Local	10,000	1,010,000	37,470,000
International	15,000	1,515,000	56,205,000
Trial monitoring & DSMB meetings	20,000	2,020,000	74,940,000
Transportation, vehicle repairs etc.	46,000	4,646,000	172,362,000
Operating expenses postage, printing etc.	12,000	1,212,000	44,964,000
TOTAL	1,471,000	148,571,000	5,511,837,000
15% contingency	220,650	22,285,650	826,775,550
**GRAND TOTAL**	**1,691,650**	**170,856,650**	**6,338,612,550**

20.2. Justification of the Budget

This work has been funded by the European and Developing Countries Clinical Trials Partnership (EDCTP). The budget includes support for all trial related activities, including community engagement. The study will not incur any consultancy fees or additional administrative overheads. Costs for patients and supplies are based on those incurred by similar vaccine trials. We have recruited laboratory research assistants who will be trained on the relevant virological and immunological studies. The clinical staff on the project will undertake Good Clinical Practice training as well as on the job training for running clinical trials.
The vaccine will be given in kind by Institut Pasteur de Dakar.

20.3. Insurance

The vaccine manufacturer is liable for any harm arising from negligent manufacture but has not undertaken to sponsor the trial. The sponsor, University of Oxford, provides insurance to cover the clinical trial participants. KEMRI CGMRC and Epicentre, Mbarara will provide indemnity for any clinical negligence at their respective sites.

21. TRIAL MANAGEMENT

University of Oxford/KEMRI CGMRC takes responsibility for initiating, registering and conduct of the trial, and as such, will be involved in the study design, collection, management and analysis, and interpretation of data, and writing of the report. The Sponsor takes responsibility for ensuring the trial is monitored properly and results made available.

Trial PIs in each site (i.e. KEMRI CGMRC and Epicentre Mbarara) will communicate regularly to coordinate on any challenges experienced by either site or necessary amendments requiring ethical review. A DSMB will be convened by the Sponsor and will receive safety data as described above.

22. REPORTING, DISSEMINATION AND NOTIFICATION OF RESULTS

Results will be published in a journal providing an open-access option. Anonymized data on immunogenicity and other trial outcomes will be made available within these publications. We will feedback individual results with clinical relevance to participants in real-time. We will feedback individual randomization arm to participants once the trial has been completed and the study team unblinded. Summaries of the outcomes of the trial will be provided during community meetings in the areas from which participants are recruited.

23. APPENDICES

23.1. Roles of Investigators

INVESTIGATOR	INSTITUTION	ROLE
George Warimwe	KEMRI CGMRC	PI. Design, conduct, data quality control and assurance. Analysis, interpretation of results and publication.
Philip Bejon	KEMRI CGMRC	Sponsor’s representative.
Sassy Molyneux	KEMRI CGMRC	Qualitative study, design, analysis, interpretation of results and publication.
Mainga Hamaluba	KEMRI CGMRC	Head of Clinical Trials facility, data quality control, assurance and analysis, interpretation of results and publication.
Henry Karanja	KEMRI CGMRC	Sample preparation and storage, lab assays and analysis, interpretation of results and publication.

John Gitonga	KEMRI CGMRC	Sample preparation and storage, lab assays and analysis, interpretation of results and publication.
Marianne Munene	KEMRI CGMRC	Regulatory affairs contact, interpretation of results and publication
Maria Namulwana	Epicentre, Mbarara	Site investigator (Mbarara) and study team lead. Design, conduct, recruitment, safety monitoring, data collection, site coordination, interpretation of results and publication.
Juliet Mwanga	Epicentre, Mbarara	Director, MSF Epicentre, Mbarara. Data quality control, assurance and analysis, interpretation of results and publication.
Dan Nyehangane	Epicentre, Mbarara	Sample preparation and storage, lab assays and analysis, interpretation of results and publication.
Edgar Mulogo	MUST, Mbarara	Oversight of conduct of the study

23.2. References

1. Shearer FM, Longbottom J, Browne AJ, Pigott DM, Brady OJ, Kraemer MUG, Marinho F, Yactayo S, de Araújo VEM, da Nóbrega AA *et al*: **Existing and potential infection risk zones of yellow fever worldwide: a modelling analysis**. *Lancet Glob Health* 2018, **6**(3):e270-e278.
2. Barrett AD, Higgs S: **Yellow fever: a disease that has yet to be conquered**. *Annu Rev Entomol* 2007, **52**:209-229.
3. Monath TP, Barrett AD: **Pathogenesis and pathophysiology of yellow fever**. *Adv Virus Res* 2003, **60**:343-395.
4. Gotuzzo E, Yactayo S, Córdova E: **Efficacy and duration of immunity after yellow fever vaccination: systematic review on the need for a booster every 10 years**. *Am J Trop Med Hyg* 2013, **89**(3):434-444.
5. Garske T, Van Kerkhove MD, Yactayo S, Ronveaux O, Lewis RF, Staples JE, Perea W, Ferguson NM, Yellow Fever Expert C: **Yellow Fever in Africa: estimating the burden of disease and impact of mass vaccination from outbreak and serological data**. *PLoS Med* 2014, **11**(5):e1001638.
6. Shearer FM, Moyes CL, Pigott DM, Brady OJ, Marinho F, Deshpande A, Longbottom J, Browne AJ, Kraemer MUG, O'Reilly KM *et al*: **Global yellow fever vaccination coverage from 1970 to 2016: an adjusted retrospective analysis**. *Lancet Infect Dis* 2017, **17**(11):1209-1217.

DocuSign Envelope ID: 6B9F30A5-D2C8-45EA-9227-845DBD009D50

7. World Health Organization (WHO): **Yellow Fever Situation Report**. In.; 2016.
8. Pan American Health Organization (PAHO): **Epidemiological Update: Yellow Fever**. In.; 2018.
9. Beck AS, Barrett AD: **Current status and future prospects of yellow fever vaccines**. *Expert Rev Vaccines* 2015, **14**(11):1479-1492.
10. World Health Organization (WHO): **Recommendations to assure the quality, safety and efficacy of live attenuated yellow fever vaccines (Annex 5)**. In.; 2013.
11. Vannice K, Wilder-Smith A, Hombach J: **Fractional-Dose Yellow Fever Vaccination - Advancing the Evidence Base**. *N Engl J Med* 2018.
12. PATH: **Yellow Fever vaccination: The potential of dose-sparing to increase vaccine supply and availability**. In.; 2013.
13. Ahuka-Mundeke S, Casey RM, Harris JB, Dixon MG, Nsele PM, Kizito GM, Umutesi G, Laven J, Paluku G, Gueye AS *et al*: **Immunogenicity of Fractional-Dose Vaccine during a Yellow Fever Outbreak - Preliminary Report**. *N Engl J Med* 2018.
14. Lopes Ode S, Guimarães SS, de Carvalho R: **Studies on yellow fever vaccine. III--Dose response in volunteers**. *J Biol Stand* 1988, **16**(2):77-82.
15. Roukens AH, Vossen AC, Bredenbeek PJ, van Dissel JT, Visser LG: **Intradermally administered yellow fever vaccine at reduced dose induces a protective immune response: a randomized controlled non-inferiority trial**. *PLoS One* 2008, **3**(4):e1993.
16. Martins RM, Maia Mde L, Farias RH, Camacho LA, Freire MS, Galler R, Yamamura AM, Almeida LF, Lima SM, Nogueira RM *et al*: **17DD yellow fever vaccine: a double blind, randomized clinical trial of immunogenicity and safety on a dose-response study**. *Hum Vaccin Immunother* 2013, **9**(4):879-888.
17. de Menezes Martins R, Maia MLS, de Lima SMB, de Noronha TG, Xavier JR, Camacho LAB, de Albuquerque EM, Farias RHG, da Matta de Castro T, Homma A *et al*: **Duration of post-vaccination immunity to yellow fever in volunteers eight years after a dose-response study**. *Vaccine* 2018, **36**(28):4112-4117.
18. Monath TP: **Review of the risks and benefits of yellow fever vaccination including some new analyses**. *Expert Rev Vaccines* 2012, **11**(4):427-448.
19. Fox JP, Penna HA: **Behavior of 17D yellow fever virus in rhesus monkeys: relation to substrain, dose and neural or extraneural inoculation.** *Am J Epidemiol* 1943, **38**(2):152-172.
20. Staples JE, Gershman M, Fischer M, Centers for Disease C, Prevention: **Yellow fever vaccine: recommendations of the Advisory Committee on Immunization Practices (ACIP)**. *MMWR Recomm Rep* 2010, **59**(RR-7):1-27.
21. Roukens AH, Soonawala D, Joosten SA, de Visser AW, Jiang X, Dirksen K, de Gruijter M, van Dissel JT, Bredenbeek PJ, Visser LG: **Elderly subjects have a delayed antibody response and prolonged viraemia following yellow fever vaccination: a prospective controlled cohort study**. *PLoS One* 2011, **6**(12):e27753.
22. Watson AM, Klimstra WB: **T Cell-Mediated Immunity towards Yellow Fever Virus and Useful Animal Models**. *Viruses* 2017, **9**(4).
23. Ochieng C, Ahenda P, Vittor AY, Nyoka R, Gikunju S, Wachira C, Waiboci L, Umuro M, Kim AA, Nderitu L *et al*: **Seroprevalence of Infections with Dengue, Rift Valley Fever and Chikungunya Viruses in Kenya, 2007**. *PLoS One* 2015, **10**(7):e0132645.
24. Demina AV, Lutwama J, Hertz T, Lobel L: **Assessing the serological antibody repertoire to Flaviviruses in the endemic population of the Zika forest in Uganda**. *J Immunol* 2017, **198**(1 Supplement).

DocuSign Envelope ID: 6B9F30A5-D2C8-45EA-9227-845DBD009D50

25. Chung AW, Alter G: **Systems serology: profiling vaccine induced humoral immunity against HIV**. *Retrovirology* 2017, **14**(1):57.
26. Selva KJ, van de Sandt CE, Lemke MM, Lee CY, Shoffner SK, Chua BY, Davis SK, Nguyen THO, Rowntree LC, Hensen L *et al*: **Systems serology detects functionally distinct coronavirus antibody features in children and elderly**. *Nature Communications* 2021, **12**(1):2037.
27. Suscovich TJ, Fallon JK, Das J, Demas AR, Crain J, Linde CH, Michell A, Natarajan H, Arevalo C, Broge T *et al*: **Mapping functional humoral correlates of protection against malaria challenge following RTS,S/AS01 vaccination**. *Sci Transl Med* 2020, **12**(553).

Summary of Changes

Protocol Title: **Non-inferiority fractional doses trial for yellow fever vaccine**

Protocol version	Date	Modifications
1.0	23rd Oct 2018	
1.1	7th Nov 2018	Clarifications and edits following institutional review by the KEMRI-Wellcome Trust Research Programme Centre Scientific Committee:<br>• Updated introduction section with background on potential generalizability of the trial results, longevity of immunity from fractional doses and how the trial differs from previous studies<br>• Deletion of repeated content in section 8.2.2
1.2	14th Jan 2019	Clarifications and edits following ethics review by the KEMRI Scientific and Ethics Review Unit (SERU):<br>• Inclusion of DSMB plan; section 18.7<br>• Clarification of secondary objectives and endpoints; sections 7.3, 11.2, 12 and 13.2.3<br>• Updates to the introduction section regarding aims of the study<br>• Updates to SAE reporting timelines in Table 4<br>• Clarification of site responsibilities; Section 21<br>• Addition of an investigator at the Mbarara site
1.3	4th Mar 2019	• Table 3 updated to reflect the changes in the stakeholders identified for the interviews. Change was made because of the logistical difficulties we anticipate having the targeted stakeholders in a focus group discussion.<br>• Clarified the record-keeping plan; Section 19.4<br>• Clarified that it is the responsibility of the PI to report SAEs to the vaccine manufacturer and the DSMB, and to co-ordinate the safety monitoring and reporting in Section 12.8.
1.4	26th Nov 2020	• Inclusion of ClinicalTrials.gov registration number, NCT04059471<br>• Amended to only include children between 9 and 12 months instead of 9 months to 5 years with the Yellow

		Fever vaccine co-administered with the routine EPI Measles vaccine; section 8.1, 10.6<br>• Amended to include measurement of antibodies against other locally circulating viruses and their impact of immunogenicity; section 7.3, 11.2<br>• Change of the Site Investigator at the Mbarara site
1.5	11th Jun 2021	• Inclusion of Jenner Institute as part of the collaborating labs where serum would be shipped for systems serology analysis; section 11.2<br>• Addition of two investigators at the Kilifi site<br>• Amendment to remove one investigator at the Kilifi site who is no longer involved with study activities
1.6	5th Jan 2023	• Amendment to remove 4 study investigators who are no longer involved with study activities

Statistical Analysis plan for the randomized, blinded non-inferiority trial on the immunogenicity and safety of fractional doses of yellow fever vaccines (NIFTY)

Version: 1.0 Date: March 24, 2021

This document has been written based on information contained in the study protocol version 1.4, dated November 26, 2020. The information contained herein is confidential and therefore are provided in confidence as a potential investigator.

	NAME	ROLE	SIGNATURE	DATE
Written by:				
Reviewed by:				
Approved by:				

SAP Version History

Protocol version	SAP version no	Version Date	Changes/Revisions
1.4	1.0	24.03.2021	First version

General Information

**Protocol Number:**	**SERU3797**
**Trial Registration Number:**	**ClinialTrials.gov NCT04059471**
**Investigational Product(s):**	Yellow Fever Vaccine
**Funder:**	European & Developing Countries Clinical Trials Partnership (EDCTP)
**Tel:**	+31 70 344 0880
**Email:**	info@edctp.org
**Sponsor:**	University of Oxford, UK
**Tel:**	Tel: +254(0)709983549
**Email:**	pbejon@kemri-wellcome.org
**Drug/Product Manufacturer:**	Institut Pasteur de Dakar, Sénégal

Signature Page

I give my approval for the attached SAP entitled Non-inferiority trial on the immunogenicity and safety of fractional doses of yellow fever vaccines dated 24/Mar/2021

Data Manager

Name:

Signature:

Date:

Statistician

Name:

Signature:

Date:

Co-Principal Investigator

Name:

Signature:

Date:

Principal Investigator

Name:

Signature:

Date:

Table of Contents

Signature Page ***1***

1. Introduction ***4***

1.1 Purpose and Scope of the Statistical Analysis Plan **4**

1.2 Trial Summary **4**

2. Study Objectives ***5***

2.1 Primary Objectives **5**

2.2 Secondary Objectives **5**

3. Study Design and Assessment ***6***

3.1 Study Design **6**

3.1.1 Study Cohorts 6

3.1.2 Inclusion Criteria 7

The inclusion criteria will be used at screening to identify participants eligible for the study and will be checked prior to vaccination to confirm ongoing eligibility. 7

3.1.3 Exclusion Criteria 7

3.1.4 Withdrawal/Discontinuation Criteria 7

3.2 Sample Size and Power Considerations **8**

3.3 Randomization and Blinding **9**

Consenting participants who have satisfied all the eligibility criteria and completed the baseline assessment will be randomized to receive one of the four vaccine doses. The allocation will be to one of the four treatment arms per a computer-generated randomization schedule. Randomization will be done by randomization booklets with concealed scratch cards, allocated in order of recruitment and opened on the day of vaccination. These will be prepared by a person outside the study. To minimize vaccine wastage, randomization will be done in block sizes that match with clinic visit days. Allocations will be concealed until a member of the unblinded study team scratches the randomization booklet to reveal the participants' randomization arm. **9**

All participants will receive the same information regarding adverse events. Participants will not be informed of the allocated vaccine. The vaccine will be prepared outside of the view of the participants. **9**

3.4 Selection of lowest non-inferior dose group for sub-study 9

3.5 Definition of Analysis Populations 10

3.6 Protocol Deviations 10

4. Assessment of Objectives 11

4.1 Immunogenicity assessments 11

4.1.1 Primary non-inferiority outcomes 11

4.1.2 Secondary non-inferiority outcomes 11

4.2 Safety Assessments 12

4.2.1 Serious Adverse Events 12

4.2.2 Adverse Events 12

4.2.3 Immediate post vaccination reactions 13

4.2.4 Other Assessments 13

5. Statistical Methods 13

5.1 Analysis timepoint 13

5.1.1 Primary Analysis Time Point, Interim Analysis and Data Monitoring Committee 13

5.1.2 Qualitative Data Analysis 14

5.2 Methods for Handling Missing Data 14

5.3 Statistical Analysis and Tests 14

5.3.1 Baseline Characteristics 14

5.3.2 Immunogenicity Analysis 15

5.3.3 Safety Analysis 16

5.3.4 Statistical Software 16

6. Statistical Tables to be Generated 16

6.1 Demographic and Baseline 16

6.2 Immunogenicity Analysis 17

6.2 Safety Analysis 19

7. Statistical Listings to be Generated 21

8. References 21

1. Introduction

1.1 Purpose and Scope of the Statistical Analysis Plan

This statistical analysis plan (SAP) details the proposed statistical analysis of the primary and secondary variables and other data resulting from the European & Developing Countries Clinical Trials Partnership (EDCTP) funded randomized, blinded non-inferiority trial on the immunogenicity and safety of fractional doses of yellow fever vaccines (NIFTY) in Kenyan and Ugandan adults and children. The results reported in the main paper(s) reporting results from this trial should follow the strategy set out in this document. Subsequent analyses of a more exploratory nature will not be bound by this strategy, though they are expected to follow the broad principles laid down here. The principles are not intended to curtail exploratory analysis, nor to prohibit accepted practices (such as data transformation prior to analysis), but they are rather intended to establish the rules that will be followed, as closely as possible, when analysing and reporting the trial.

The analysis strategy will be available on request when the principal papers are submitted for publication in a journal. Suggestions for analyses by journal editors or referees will be considered carefully and carried out, as far as possible, in line with the principles of this analysis plan; if reported, the source of the suggestion will be acknowledged.

Any deviation(s) from the SAP will be described and justified in the final report of the trial. The analysis should be carried out by an identified, appropriately qualified and experienced statistician, who should also ensure the integrity of the data during their processing through quality control and evaluation procedures.

1.2 Trial Summary

In July 2016, the demand for yellow fever (YF) vaccines in response to the large urban outbreaks occurring concurrently and the risk of further spread through Africa and to Asia was larger than the available global supply. In this situation, the World Health Organization (WHO) developed recommendations for the use of fractional doses of YF vaccine as a dose-sparing strategy. These recommendations were based on data from a limited number of clinical trials, none of which had been conducted in Africa. Additional studies were initiated to assess the applicability of fractional doses to all four WHO-prequalified YF vaccines with respect to vaccine immunogenicity in adults and children in Africa, including HIV positive adults. One such study, comparing full standard dose to 1/5th of standard dose of all four WHO-prequalified YF vaccines in adults (Clinicaltrials.gov number: NCT02991495), is currently ongoing at KEMRI CGMRC (see SERU protocol 3452) and Epicentre, Mbarara (UNCST HS 2237) and is designed to answer questions on the use of current stock of YF vaccines with a potency as close as possible to each manufacturers' minimum release. Data from this trial will inform a WHO recommendation on using 1/5th of the current standard dose of vaccine for outbreak control. However, since many vials will contain excess YF vaccine such that 1/5th of a vial is

likely to be substantially above the current minimum potency requirements, these data may not be scientifically explanatory regarding the minimum dose required for preventive use. Here, in this new complementary study, we aim to determine the lowest YF vaccine dose that is non-inferior to the current standard full dose among populations in sub-Saharan Africa (SSA). The study will be conducted in Kenya (KEMRI CGMRC, Kilifi) and Uganda (Epicentre, Mbarara) with trial participants recruited at both sites, using vaccine from one WHO-prequalified manufacturer (Institut Pasteur de Dakar, Senegal). Our primary aim is to compare the immunogenicity of full standard dose of vaccine with three lower doses in adults, using seroconversion at 28 days post-vaccination, measured by plaque reduction neutralization assay ($PRNT_{50}$), as the endpoint. Adult participants (n=480) will be randomized for vaccination with full standard dose or with approximately 1000, 500 or 250 IU of vaccine (i.e. 4 arms, 1:1:1:1 allocation ratio). Safety and immunogenicity results will then be reviewed by the study Data and Safety Monitoring Board (DSMB), and the lowest non-inferior dose selected for assessment in a sub-study in children (n=420) in comparison to full standard dose (i.e. 2 arms, 1:1 allocation ratio) co-administered with the Expanded Program for Immunisation (EPI) Measles vaccine. Secondary objectives will include immunogenicity at 10 days, 1 year and 2 years post-vaccination, assessment of the influence of cross-reactive antibodies to other flaviviruses on vaccine immunogenicity, assessment of T and B cell immune responses, assessment of virologic and immunological kinetics and occurrence of serious adverse events (SAE). In addition, we will assess the range of views and perceptions of key stakeholders in vaccine policy and implementation on lower vaccine dose usage during YF epidemics and routine use. We expect the data from this qualitative study to inform the implementation of policies relating to low-dose vaccine usage for the control of YF and other diseases (e.g. pneumococcal vaccines) where such strategies are in consideration.

2. Study Objectives

2.1 Primary Objectives

To determine the lowest dose (1000, 500 and 250 IU/dose) of YF vaccine that is non-inferior to the full standard dose as measured by seroconversion using the $PRNT_{50}$ assay at 28 days post-vaccination.

Seroconversion is defined as a ≥4-fold rise in neutralizing antibody titre between pre-immunization (Day 0) and post-immunization samples. Any patient who appears seronegative ($PRNT_{50}$ value below the Limit of Quantification, 1:10) will be considered to have a baseline titre of LOQ/2, 1:5. Noninferiority will be assessed by comparing the lower dose to the full dose of vaccine. Since baseline antibody levels can modulate response to the vaccine, the primary analysis will be based on the per-protocol (PP) population, which must be seronegative to YF at baseline. Secondary analysis of seroconversion at Day 28 will be performed in the intent-to-treat (ITT) population, regardless of baseline YF seropositive, and for the subset of adults which are seropositive to YF at baseline. The primary analysis time point will be when each participant has provided a Day 28 post-vaccination blood sample or has surpassed the window (+/- 3 days) to provide this sample.

2.2 Secondary Objectives

There are multiple secondary objectives to assess non-inferiority, safety, and immunogenicity in specific populations. These are:

- To describe the geometric mean $PRNT_{50}$ titre (GMT) at 10 days, 28 days, 1 year and at 2 years post-vaccination of the different doses of the YF vaccine.
- To describe the change in $PRNT_{50}$ titre (i.e. the geometric mean fold increase (GMFI) as a continuous variable) between baseline and day 28 after vaccination with the different doses of the YF vaccine.
- To measure neutralizing antibody to other flaviviruses (including dengue, West Nile and zika viruses) on the baseline sample and determine the impact of these antibodies on YF vaccine immunogenicity.
- To assess post-vaccination control of viremia by vaccine dose on samples collected at baseline, and on days 2, 3, 4, 5, 6, 7 and 10 after vaccination.
- To determine the change in T and B cell immune responses between baseline and days 10 and 28 post-vaccination.
- To determine the change in serum cytokine and chemokine levels between baseline and days 2, 3, 4, 5, 6, 7, 10 and 28 post-vaccination.
- To assess the occurrence of adverse events (AE) over 28 days after vaccination and serious adverse events throughout the duration of the study.
- Map out key stakeholders' priorities and perceptions regarding a change in policy towards the use of lower doses of YF vaccine and their potential influence on policy process.

Once results for the main outcome are obtained, data will be reviewed by the study DSMB. The DSMB will then decide if the study should proceed to the second phase where the lowest non-inferior dose, as measured at day 28 in the adult study, will be selected for assessment in children aged 9-12 months co-administered with the Measles vaccine. The aim of these studies will be to: assess the non-inferiority in seroconversion of the lower dose compared to the full standard dose as measured by $PRNT_{50}$ at 28 days post-vaccination.

3. Study Design and Assessment

3.1 Study Design

3.1.1 Study Cohorts

We will use YF vaccine produced by the Institut Pasteur de Dakar, Senegal. One vial format will be provided. It will contain standard full dose of vaccine for administration in 0.5ml per dose. A second vial will be prepared from a standard vial to contain vaccine diluted to approximately 1000 IU/dose for administration in 0.5ml per dose. Fractional volumes of this second vial will be administered as per manufacturer's instructions to achieve the 500 IU and 250 IU doses. There will be two study cohorts of interest:

A: General adult population (n=480); and

B: Children 9-12 months (n=420)

Results for the safety and primary outcome of the adult study will then be reviewed by the DSMB, and the lowest non-inferior dose in the adult study selected for assessment in children aged 9 -12

months sub-study. Should there be evidence of inferiority of the lower dose vaccine, the sub-studies in populations (B) will not be conducted.

3.1.2 Inclusion Criteria

The inclusion criteria will be used at screening to identify participants eligible for the study and will be checked prior to vaccination to confirm ongoing eligibility.

- For cohort (A) Individuals aged ≥18 - <60 years of age.
- For cohort (B) (i.e. children): Individuals 9-12 months
- HIV negative on serological screening OR
- HIV positive adults on serological testing, and no symptoms suggestive of current clinical immunosuppression and CD 4 count>200 (for adults) and CD4% > 25% (for children) within the last 6 months.
- Ability to provide informed consent to participate in the study

3.1.3 Exclusion Criteria

- Known contraindications to YF vaccination such as allergies to egg protein and chicken products or any component of the vaccine (including gelatin, eggs, eggs products or chicken products), immunodeficiency, known thymus disorder, such as thymoma and myasthenia gravis
- Using corticosteroids or other immunosuppressive therapy
- Thymus disorder, such as thymoma and myasthenia gravis
- Acute febrile disease on the day of vaccination with temperature >37.5 degrees Celsius is a temporal contraindication.
- Previous YF vaccination
- Previous YF infection as determined from history
- Pregnancy (as determined by a urine test on the proposed day of vaccination) and lactating women
- Planning to migrate out of the study areas before the end of the study follow-up
- Planning to travel to a country requiring YF vaccination certificate within the first year after vaccination.
- Any condition or criteria, including acute or chronic clinically significant abnormality that in the opinion of the investigator might compromise the wellbeing of the volunteer or interfere with the outcome of the study.

3.1.4 Withdrawal/Discontinuation Criteria

In accordance with the principles of the Declaration of Helsinki, participants have the right to withdraw from the study at any time and for any reason and are not obliged to give their reason for doing so.

Participants may be discontinued for the following reasons:

- SAE which requires discontinuation of study involvement or which results in inability to continue to comply with study procedures;
- Protocol violation requiring discontinuation from study;

- Withdrawal of consent;
- Participant moves out of the study area and/or cannot be traced
- Any other reason determined by participant or study staff

The study team will continue to follow up all participants, with their agreement, until the end of the study. The reason for withdrawal will be recorded in the Case Report Form (CRF). If withdrawal is due to a SAE, appropriate follow-up visits or medical care will be arranged, with the agreement of the participant, until the event has resolved. Following Good Clinical Practice (GCP) guidelines, data on participants who specifically withdraw their consent for use of their data will not be included in the data analysis. However, participants who withdraw from follow up without withdrawing consent for use of their data will be included.

3.2 Sample Size and Power Considerations

This study will be powered to detect non-inferiority of each lower dose of vaccine (1000 IU, 500 IU, 250 IU) compared to the full standard vaccine dose.

For the adult study, assuming a 95% seroconversion rate, 90% power, 2.5% level of significance for a one-sided test and a non-inferiority margin of 10%, which gave a sample size of 100 per arm. The 10% non-inferiority margin was chosen in consideration of the public health consequence of a loss of protection but a potential increase in vaccine dosages in a situation where vaccine stocks are insufficient to respond to an outbreak. The sample size was increased by 20% to account for: i) losses to follow up and, ii) unevaluable participants with a positive serological response for YF virus at baseline. Thus, a total sample size of 480 will be required for the four vaccine dose groups (i.e. full dose, 1000 IU, 500 IU and 250 IU).

For the study in children, we assumed a 90% seroconversion rate (accounting for lower vaccine immunogenicity reported in children [4]), 90% power, 2.5% alpha for a one-sided test and a non-inferiority margin of 10%, which gave a sample size of 190 per arm. This was increased by 10% to account for 5% losses to follow up and 5% unevaluable participants with a positive serological response for YF virus at baseline. This gave a total sample size of 420 (i.e. 210 in the full dose group and 210 in the lower dose group.

Analyses of all other efficacy and safety endpoints are supporting only. Therefore, no significant adjustments will be made for multiple comparisons.

3.3 Randomization and Blinding

Consenting participants who have satisfied all the eligibility criteria and completed the baseline assessment will be randomized to receive one of the four vaccine doses. The allocation will be to one of the four treatment arms per a computer-generated randomization schedule. Randomization will be done by randomization booklets with concealed scratch cards, allocated in order of recruitment and opened on the day of vaccination. These will be prepared by a person outside the study. To minimize vaccine wastage, randomization will be done in block sizes that match with clinic visit days. Allocations will be concealed until a member of the unblinded study team scratches the randomization booklet to reveal the participants' randomization arm.

All participants will receive the same information regarding adverse events. Participants will not be informed of the allocated vaccine. The vaccine will be prepared outside of the view of the participants.

The children will be randomized to either full dose or the lowest non-inferior dose of the vaccine. The vaccine dose to be used will be selected based on the safety and immunogenicity results at day 28 post-vaccination from the adult study. Similar randomization procedures as the adult study will be used. The children will be vaccinated in collaboration with a Ministry of Health EPI clinic for the Measles vaccine.

3.4 Selection of lowest non-inferior dose group for sub-study

Following the primary analysis time point, the DSMB will determine whether there is sufficient evidence of efficacy and safety to continue the trial in Cohort(B) and will offer a recommendation on which lowest non-inferior dose group to use in the sub-studies.

First, the DSMB shall assess non-inferiority at Day 28 of seroconversion rates (primary analysis), GMT, and GMFI for each dose groups in the adult PP and ITT populations. Any vaccine dose group (i.e.1000 IU, 500 IU, 250 IU) that is shown to be non-inferior in seroconversion and for which no concerns regarding significant variation are noted on secondary analysis will be considered for use in the sub-studies. If the lower doses are inferior to the full dose of all four vaccines, the sub-studies in populations (B) will not be conducted.

The primary endpoint will be assessed 28 days post-vaccination, but study visits will continue for up to two years after vaccination. Venous blood samples for YF titre quantification by PRNT will be collected at baseline, Viremia visit (2-7 days),10 days (+/- 1 day), 28 days (+/- 3 days), 1 year (+/- 14 days) and 2 Years (+/- 28 days) after vaccination. PRNT for paired samples will be run at the same time to assure comparability of measurements from samples at baseline, 10 days, 28 days, 365 days and 2 years post-vaccination. The quantification of antibodies will be done at the Institut Pasteur in Dakar, Senegal. PRNT is considered the most sensitive and specific test for quantification of neutralizing antibodies and is the reference method for assessing immune response after vaccination.

Table 1: Study activities timeline

Procedure	Screening Day -30*	Day 0	Day 2, 3, 4, 5, 6, 7**	Day 10 (+/- 1 day)	Day 28 (+/- 3 days)	Day 365 (+/-14 days)	Day 730 (+/- 28 days)
Informed Consent	X						
HIV Antibody test	X						
Pregnancy test	X	X			X		
Demography	X	X					
Vital signs	X	X		X	X	X	X
History and Physical exam	X			X	X	X	X
Randomization		X					
Vaccination		X					
Blood sample (Adults, Children)		X (10ml, 6ml)	X (4ml, 4ml)	X (10ml, 6ml)	X (10ml, 6ml)	X (10ml, 6ml)	X (10ml, 0ml)
Cumulative blood volume (Adults, Children)		10ml, 6ml	14ml, 10ml	24ml, 16ml	34ml, 22ml	44ml, 28ml	54ml, 28ml
Adverse events and serious Adverse Event			X	X	X	X	X

*Before vaccination. Screening can occur between 0 and 30 days before vaccination. Screening and vaccination can occur on the same day, but participants that are not recruited within 30 days will be re-screened.

**All participants will be randomized to provide one 4ml blood sample at one of 6 time points (days 2, 3, 4, 5, 6, or 7).

**Note that vaccination will generally occur on same day of enrolment, but under certain circumstances may necessarily be delayed to a later date. Day 0 will always be considered date of vaccination.*

3.5 Definition of Analysis Populations

The per-protocol (PP) population will include randomized participants who have a blood sample at baseline and 28 days (+/- 3 days) post-vaccination, who are seronegative ($PRNT_{50}$ <1:10) to YF at baseline and for whom the eligibility criteria were correctly applied. The intention-to-treat (ITT) population will comprise all randomized participants who received a dose of a study vaccine and that have at least one post-vaccination blood sample. We will also assess immunogenicity endpoints for the subset of the ITT population which has a pre-existing YF neutralizing antibodies ($PRNT_{50}$≥1:10) at baseline (ITT YF+). As above, the same definitions will be applied to the sub-population to generate, Children ITT and Children ITT YF+ populations. The same noninferiority and safety endpoints assessed for the PP and ITT populations will be analyzed for cohort (B).

The safety (SAF) population will include all participants who received a study vaccine.

3.6 Protocol Deviations

Any deviation from protocol-specified procedures and study-related SOPs occurring during the conduct of the trial will be documented and reported as protocol violations (major deviations) or minor deviations, as defined in the protocol. Protocol violations will be reported to the Sponsor and

reviewing ethical committees, as appropriate and in accordance with the requirements of the involved committees. A final Protocol Deviation Listing will be generated and reviewed blindly by sponsor personnel prior to freezing the database to ensure that all important deviations, including those that may lead to exclusion from analysis, are captured and summarized. No formal statistical testing will be undertaken.

Subjects will be excluded entirely from analysis if they have a protocol deviation defined as a full exclusion that affects the validity of all their data (e.g. failure to obtain informed consent). Subjects with a partial protocol deviation (e.g. moving away from study area) will be included up until the time of the deviation; from this point onwards, their data will be excluded from analysis. If a deviation affects data at a specific time point (e.g. blood sample not taken within defined time window), only data affected by the deviation will be removed from analysis.

4. Assessment of Objectives

4.1 Immunogenicity assessments

4.1.1 Primary non-inferiority outcomes

The primary outcome is non-inferiority of the proportion of participants in the PP population who seroconvert by Day 28 for the lower dose (i.e.1000 IU, 500 IU, 250 IU) compared to the full dose. Seroconversion is defined as a ≥4-fold rise in neutralizing antibody titre between pre-immunization Day 0 and post-vaccination samples. Any $PRNT_{50}$ value reported as seronegative (below the Limit of Quantification, <1:10) will be converted to LOQ/2. Thus a 4-fold rise for a subject who is <1:10 at baseline, is a titre of 20.

4.1.2 Secondary non-inferiority outcomes

Geometric mean $PRNT_{50}$ titre (GMT) on Day 0 and Day 28 will be calculated in the PP population. A test of non-inferiority will be performed for the difference in $\log_{10}$ GMT from baseline to Day 28 between full-dose and lower dose. Geometric fold increase in $PRNT_{50}$ titre (GMFI) between Day 0 and Day 28 in the PP population will also be calculated and a similar test of non-inferiority performed. Again, any $PRNT_{50}$ value reported as below the Limit of Quantification (LOQ) will be converted to a titre of 1:5.

The above non-inferiority assessments of seroconversion, GMT and GMFI at Day 28 will also be assessed in the ITT population, in the subset of the ITT population with pre-existing YF neutralizing antibodies at baseline (ITT FV+), and in the subset of the PP population with no reported history of flavivirus infection (PP FV-).

Lower vaccines doses may change the kinetics of antibody response. The assessment of seroconversion rates, GMT, and GMFI 10 days after vaccination in the ITT population will provide important information in the context of fractional dosing in outbreak response. The three immunogenicity outcomes will also be assessed in 2 years post-vaccination in the ITT population to confirm a lasting effect of full and lower dose vaccination.

Each of the above primary and secondary immunogenicity analyses will be performed in the primary study cohort (A) and replicated in the Children (B) cohort, should the sub-studies proceed.

4.2 Safety Assessments

The safety of lower and full dose vaccines will be assessed by recording adverse events (AEs) and serious adverse events (SAEs) occurring in the safety population (i.e., those who are randomized and receive a vaccine). Safety outcomes will be actively monitored at the scheduled visits (2-7),10 and 28 days post-vaccination. Participants will also be advised to report any reactions that concern them to research staff throughout the study.

Any safety event, regardless of seriousness, will be recorded in the CRF. A detailed clinical description of the event using standardized forms will be provided, including the diagnosis, the date of onset and resolution of episode, and outcome. Each event will be coded using MedDRA dictionary, Version 20.0 System Organ Classes and Preferred Terms. The exact version of the dictionary will be mentioned in the footnote of the respective Listing and/or Table.

Each event will be assessed for the degree of certainty with which it can be attributed to vaccination based on previous reports of similar events after YF vaccination or other similar types of vaccination and the temporal association of the event with vaccination. If there is a reasonable possibility that vaccination contributed to the adverse event, the event will be classified as related to vaccination. If there is no suspicion that the event is related to vaccination (e.g, the event does not follow a reasonable temporal trajectory), it will be classified as unrelated. Any event for which a determination cannot be made will be considered unclassified.

4.2.1 Serious Adverse Events

A serious adverse event (SAE) is any untoward medical occurrence that either results in death; is life threatening; requires in-patient or extended hospitalization; results in persistent or significant disability or incapacity, or in a congenital anomaly in participant’s offspring; or is a medically important event that may jeopardize the participant or require intervention to avoid one of the above outcomes.

SAEs will be actively monitored in the entire safety population at the Day (2-7), Day 10 and Day 28 post vaccination visits. Participants will be instructed to contact the study team to report any possible SAEs (passive assessment) up until the conclusion of the study. All SAEs will be recorded and followed up to resolution, irrespective of the severity and relationship to vaccination.

In addition to the assessments above, investigators will evaluate whether a SAE is an expected reaction to YF vaccination. A SAE will be considered unexpected if the nature, seriousness, severity, or outcome of the event is not consistent with the vaccine’s known effects as described in the medical literature and described by the manufacturer in the product specifications. An unexpected SAE determined to be related to vaccination will be clarified as a Suspected Unexpected Serious Adverse Reaction (SUSAR).

4.2.2 Adverse Events

An adverse event (AE) is defined as any untoward medical occurrence in a clinical trial participant to whom a vaccine has been administered; it does not necessarily have a causal relationship with the vaccine/vaccination. An AE can therefore be any unfavorable and unintended sign (including an abnormal laboratory finding), symptom, or disease temporally associated with the use of a medicinal (investigational) product, whether related to the medicinal (investigational) product. AEs include all

events including but not limited to injection-site reactions (pain, induration, and erythema) and systemic events (fever, headache, nausea, allergic reactions).

Adverse events will be followed for the first 28 days post-vaccination. At the Day (2-7), Day10 and day 28 post vaccination visits, participants will be asked to describe local and systemic events occurring since the previous visit. In addition to the nature, outcome, and relatedness, each AE will be graded for severity: mild (Grade 1), moderate (Grade 2), severe (Grade 3), or life-threatening or death (Grade 4; note this category would imply a SAE, which would need additional reporting details).

4.2.3 Immediate post vaccination reactions

Immediate Adverse Events (IAEs) are defined as adverse events occurring within 30 minutes post vaccination with either a full or lower dose. All participants in the Safety population will be observed for 30 minutes after vaccination to monitor for any immediate adverse events. A physician equipped with advanced life support drugs and resuscitation equipment will be available in case of anaphylactic shock. The nature, outcome, relatedness, and severity of each IAE will be recorded.

4.2.4 Other Assessments

Data on age at randomisation, sex, area of residence, prior flavivirus infection, and body temperature will be collected at the initial eligibility screening and vaccination (Day 0). Pregnancy testing will be required of all female participants at the initial visit. Pregnancy at time of enrolment is an exclusion criterion. HIV testing will be required for participation. Any individual found to be HIV-positive will complete a CD4 count to determine their baseline CD4 level for analysis. As noted above, participants with a verified CD4 count over 200 cells/mm^3 within the last 6 months may be enrolled based on this test, but CD4 count must still be quantified at baseline for use in analysis.

5. Statistical Methods

All statistical methods will be based on the International Conference on Harmonisation (ICH) E9 document "Statistical Principles for Clinical Trials".

5.1 Analysis timepoint

The participants will participate in this study to a maximum of two years. The primary endpoint will be assessed 28 days post-vaccination. The two-year visit will mark the end of the study for most participants.

5.1.1 Primary Analysis Time Point, Interim Analysis and Data Monitoring Committee

The primary analysis time point for each study cohort is 28 days post-vaccination, at which point non-inferiority of seroconversion will be assessed among those vaccinated with a lower vaccine dose compared to those vaccinated with a full dose in each study cohort.

One formal statistical interim analysis is planned on the primary outcome (or endpoint) at the primary analysis time point for review by the DSMB. This will utilize immunogenicity data at Day 0 and Day 28, and safety data collected within 28 days of vaccination for AEFIs and throughout the study for SAEs.

The DSMB will assess non-inferiority in seroconversion among adults (cohort (A) at the primary analysis time point before beginning sub-studies of lower dosing in cohort (B) children 9 months to 5 years of age. Children are potentially at higher risk of an adverse event or lower neutralizing titres (and therefore decreased protection against disease) following vaccination with a lower dose. Therefore, it is important to assess immunogenicity and safety in the primary analysis population before beginning these sub-studies. No adjustment for multiple comparisons will be made as only a single confidence interval will be constructed for each lower dose vaccine at Day 28. The DSMB will also assess safety endpoints and select secondary outcomes.

5.1.2 Qualitative Data Analysis

Analysis of qualitative data will follow the framework approach. To ensure that interpretations of quotes are consistent, and that data quality is rigorous and transparent, independent qualitative researchers will code the data; differences between coding will be resolved by group discussion involving other members of the research team. Recurring issues, concepts and patterns will be identified using ground theory and grouped according to thematic areas.

5.2 Methods for Handling Missing Data

The numbers (with reasons) of losses to follow-up (dropouts and withdrawals) over the course of the trial will be reported by trial arm. Valid inferences will be made under the missing at random assumption. No imputation will be done in any way.

5.3 Statistical Analysis and Tests

Categorical data will be summarised by numbers and percentages. Continuous data will be summarised by mean, standard deviation (SD), and range if data are normally distributed and median, interquartile range (IQR) and range if data are skewed. Minimum and maximum values will also be presented for continuous data. Data will be summarised for each study cohort and each study arm (full or lower dose). All applicable statistical tests will be performed using a 5% significance level. All confidence intervals presented will be 95%.

5.3.1 Baseline Characteristics

Information for each of the following categories will be presented for the adult study and the children sub-study:

- number of individuals screened;
- the number and reason of screen-outs;
- proportion of eligible individuals who consent and are randomized;
- the proportion of randomized individuals vaccinated;
- the proportion of randomized to viremia follow up per day (2-7);
- the proportion of vaccinated individuals who complete a day 10 post-vaccination visit;
- the proportion of vaccinated individuals who complete a day 28 visit;
- the proportion of vaccinated individuals who complete the final analysis period (defined as completing all study activities up to 12 months for the children sub-study, and up 24 months for the adult study);
- the proportion of individuals who discontinue and the reason for discontinuation.

Participant demographics and baseline characteristics such as temperature, age at randomisation, sex, area of residence, pre-existing antibodies to YF, reported history of YFV infection, and medical illness will be described both overall and separately for each vaccine dose groups (lower and full). Tests of statistical significance will not be undertaken for these demographic and baseline characteristics; rather the clinical importance of any imbalance will be noted.

5.3.2 Immunogenicity Analysis

All immunogenicity analyses will be pairwise comparisons of study arms (full vs lower dose group) within each cohort, and only a single lower dose vaccine group will be used for the sub-study in children.

Primary Non-Inferiority Analysis

The proportion of subjects who seroconvert will be presented by study arm for each study cohort, along with 95% confidence intervals. The primary analysis will be a pairwise statistical comparison of the rate of seroconversion at day 28 between full dose and each lower dose of vaccine using a non-inferiority test with a margin of non-inferiority of 10% in the PP population. Each immunogenicity assessment will be a pairwise comparison of the full dose and each lower dose within one study population (i.e. adults or children) using Dunnett's Test.

Secondary Non-Inferiority Analysis

Secondary analyses will include assessment of seroconversion in the ITT population as a whole, on the subset of the ITT population with baseline seropositivity to YF, and in the subset of the PP population with no reported history of flavivirus infection. Geometric mean $PRNT_{50}$ titre (GMT) and GMT fold increase (GMFI) and corresponding 95% confidence intervals (CI) on day 0 and 28 will be calculated. Non-inferiority will be calculated by the difference in GMT and GMFI between the full dose and each lower dose group. Titres will be graphically represented by reverse cumulative distributions obtained by plotting, for each possible value of the titre (abscissa), the proportion of subjects with a titre greater than this value.

Lower vaccines doses may change the kinetics of antibody response. The assessment of seroconversion rates, GMT, and GMFI 10 days after vaccination in the ITT population will provide important information in the context of low dose vaccine usage in outbreak response. These three immunogenicity outcomes will also be assessed at 1 year and 2 years post-vaccination in the ITT population to confirm a lasting effect of full and low dose vaccination.

Relationships between seroconversion and vaccine immunogenicity ($PRNT_{50}$ GMT and GMFI) will be related to frequencies of specific T and B cell subsets measured by flow cytometry, chemokine and cytokine levels in sera and neutralising antibody levels to other flaviviruses. Correlations between vaccine viraemia and immunogenicity will be assessed across the different vaccine dose strata. Comparisons of immune and viraemia kinetics will be made between the adult and children trial participants using non-parametric tests, whilst accounting for the administered vaccine dose.

Adverse events occurring during the study follow up period will be analyzed and compared between groups. This will be a descriptive analysis and will include all AEs up to 28 days post-vaccination, and SAEs that occurred any time during study follow-up.

Pre-existing immunity interference

We will perform a logistic regression to evaluate the impact of vaccine dose group and pre-existing immunity to YF on rate of seroconversion at Day 28. A logistic regression analysis will be conducted within each study cohort, with adjustment for age at randomization, site, sex, and baseline YF titre (as a continuous variable) and vaccine dose group as covariates. This analysis will be conducted in the subset of the ITT population which is seropositive to YF at baseline (ITT YF+) for each study cohort.

5.3.3 Safety Analysis

Safety outcomes will be assessed in the Safety population (i.e., any participant who was vaccinated) by study cohort for full and lower doses. All adverse events, serious adverse events, or deaths will be summarised as n (%) by study cohort and by study arm, where n is the number of participants that experienced any event post-vaccination and as a percentage of the total safety population within the study arm. All events (AEs, SAEs, and deaths) will also be presented by MedDRA coding (both System Organ Class and Preferred Term), relatedness to study vaccine, and outcome.

The proportion of participants in each study arm reporting any local reaction will be compared using the Chi-squared test and the difference in proportions with 95% confidence intervals will be presented. This will be repeated for systemic reactions.

All SAEs will be described in detail for each participant. The proportion of participants in each study arm reporting at least one SAE will be compared using Chi-squared test or Fisher's exact test, depending on the distributional assumptions.

5.3.4 Statistical Software

The analysis will be performed using Stata. Other packages such as R or SAS may be used if deemed necessary.

6. Statistical Tables to be Generated

6.1 Demographic and Baseline

Table 1.1	Summary of Screening and Vaccination
Table 1.2	Summary of Subject by Study Arm, Randomized Subjects and study cohort
Table 1.3	Summary of Premature Discontinuation by Study Arm, Study Cohort
Table 1.4	Summary of Protocol Deviations by Study Arm and Study Cohort.

Table 1.5	Summary of Demographics and Baseline Characteristics by Study Arm and Study Cohort

6.2 Immunogenicity Analysis

Seroconversion

Table 2.1	Non-inferiority of seroconversion rate in each lower dose vs. full dose of YF vaccine at Day 28 (Primary outcome) in adult PP population
Table 2.2	Non-inferiority of seroconversion rate in in each lower dose vs. full dose of YF vaccine at Day 28 in adult ITT population
Table 2.3	Non-inferiority of seroconversion rate in each lower vs. full dose of YF vaccine at Day 28 in adult ITT population with baseline sero-positivity to YF.
Table 2.4	Non-inferiority of seroconversion rate in each of each lower vs. full dose of YF vaccine at Day 28 in adult PP population with no reported history of flavivirus infection.
Table 2.5	Non-inferiority of seroconversion rate in each lower vs. full dose of YF vaccine at Day 10 in adult ITT population.
Table 2.6	Non-inferiority of seroconversion rate with each lower vs. full dose of YF vaccine at Day 365 in adult ITT population.
Table 2.7	Non-inferiority of seroconversion rate with each lower vs. full dose of YF vaccine at 2 years follow up in adult ITT population.
Table 2.8	Non-inferiority of seroconversion rate in each lower vs. full dose of YF vaccine at Day 28 in Children PP population
Table 2.9	Non-inferiority of seroconversion rate in each lower vs. full dose of YF vaccine at Day 28 in Children ITT population
Table 2.10	Non-inferiority of seroconversion rate in each lower vs. full dose of YF vaccine at Day 28 in Children ITT population with baseline seropositivity to YF
Table 2.11	Non-inferiority of seroconversion rate in each lower vs. full dose of YF vaccine at Day 28 in Children PP population with no reported history of flavivirus infection
Table 2.12	Non-inferiority of seroconversion rate in each lower vs. full dose of YF vaccine at Day 10 in Children ITT population
Table 2.13	Non-inferiority of seroconversion rate in each lower vs. full dose of YF vaccine at Day

	365 in Children ITT population

Geometric Mean Titre

Table 2.14	Non-inferiority of GMT in each lower vs full dose of YF vaccine at Day 28 in adult PP population
Table 2.15	Non-inferiority of GMT in each lower vs. full dose of YF vaccine at Day 28 in adult ITT population
Table 2.16	Non-inferiority of GMT in each lower vs. full dose of YF vaccine at Day 28 in adult ITT population with baseline seropositivity to YF
Table 2.17	Non-inferiority of GMT in each lower vs. full dose of YF vaccine at Day 28 in adult PP population with no reported history of flavivirus infection
Table 2.18	Non-inferiority of GMT in each lower vs. full dose of YF vaccine at Day 10 in adult ITT population.
Table 2.19	Non-inferiority of GMT in each lower dose vs. full dose of YF vaccine at Day 365 in adult<br>ITT population
Table 2.20	Non-inferiority of GMT in each lower vs. full dose of YF vaccine at 2 years in adult ITT population
Table 2.21	Non-inferiority of GMT in each lower vs. full dose of YF vaccine at Day 28 in Children<br>PP population
Table 2.22	Non-inferiority of GMT in each lower vs. full dose of YF vaccine at Day 28 in Children<br>ITT population
Table 2.23	Non-inferiority of GMT in each lower vs. full dose of YF vaccine at Day 28 in Children<br>ITT population with baseline seropositivity to YF
Table 2.24	Non-inferiority of GMT in each lower vs. full dose of YF vaccine at Day 28 in Children<br>PP population with no reported history of flavivirus infection
Table 2.25	Non-inferiority of GMT in each lower vs. full dose of YF vaccine at Day 10 in Children<br>ITT population
Table 2.26	Non-inferiority of GMT in each lower vs. full dose of YF vaccine at Day 365 in Children<br>ITT population

Geometric Mean Fold Increase

Table 2.27	Non-inferiority of GMFI in each lower vs. full dose of YF vaccine at Day 28 in adult PP population
Table 2.28	Non-inferiority of GMFI in each lower vs. full dose of YF vaccine at Day 28 in adult ITT population
Table 2.29	Non-inferiority of GMFI in each lower vs. full dose of YF vaccine at Day 28 in adult ITT population with baseline seropositivity to YF
Table 2.30	Non-inferiority of GMFI in each lower vs. full dose of YF vaccine at Day 28 in adult PP population with no reported history of flavivirus infection
Table 2.31	Non-inferiority of GMFI in each lower vs. full dose of YF vaccine at Day 10 in adult ITT population
Table 2.32	Non-inferiority of GMFI with fractional vs. full dose of YF vaccine at Day 365 in adult ITT population
Table 2.33	Non-inferiority of GMFI with fractional vs. full dose of YF vaccine at Day 2 years in adult ITT population
Table 2.34	Non-inferiority of GMFI in each lower vs. full dose of YF vaccine at Day 28 in Children PP population
Table 2.35	Non-inferiority of GMFI in each lower vs. full dose of YF vaccine at Day 28 in Children ITT population
Table 2.36	Non-inferiority of GMFI in each lower vs. full dose of YF vaccine at Day 28 in Children ITT population with baseline seropositivity to YF
Table 2.37	Non-inferiority of GMFI in each lower vs. full dose of YF vaccine at Day 28 in Children PP population with no reported history of flavivirus infection
Table 2.38	Non-inferiority of GMFI in each lower vs. full dose of YF vaccine at Day 10 in Children ITT population
Table 2.39	Non-inferiority of GMFI in each lower vs. full dose of YF vaccine at Day 365 in Children ITT population

6.2 Safety Analysis

Adverse Events

Table 3.1	Summary of adverse events up to Day 28 post-vaccination in Safety population by study cohort and study arm
Table 3.2	Adverse events up to Day 28 post-vaccination by MedDRA coding (SOC and PT) by study arm in adult SAF population

Table 3.4	Adverse events (AEs) up to Day 28 post-vaccination by MedDRA coding (SOC and PT) by study arm, Children SAF population
Table 3.5	Severity of adverse events up to Day 28 post-vaccination, by study cohort and study arm
Table 3.6	Relatedness of adverse events up to Day 28 post-vaccination, by study cohort and study arm, Safety population
Table 3.7	Outcome of adverse events up to Day 28 post-vaccination, by study cohort and study arm Safety population

Immediate Adverse Events

Table 3.8	Summary of immediate adverse events (IAEs) in Safety population by study cohort, study arm.
Table 3.9	Immediate adverse events (IAEs) by MedDRA coding (SOC and PT) by study arm and, adult SAF population.
Table 3.11	Immediate adverse events (IAEs) by MedDRA coding (SOC and PT) by study arm, Children SAF population
Table 3.12	Severity of immediate adverse events (IAEs), by study cohort, study arm in Safety population
Table 3.13	Relatedness of immediate adverse events (IAEs), by study cohort, study arm in Safety population
Table 3.14	Outcome of immediate adverse events (IAEs), by study cohort, study arm in Safety population

Serious Adverse Events

Table 3.15	Summary of serious adverse events (SAEs) by study cohort, study arm
Table 3.16	Summary of suspected unexpected serious adverse reactions (SUSARs) by study cohort, study arm, Safety population
Table 3.17	Serious adverse events (SAEs) by MedDRA coding (SOC and PT) by study arm adult SAF population
Table 3.19	Serious adverse events (SAEs) by MedDRA coding (SOC and PT) by study arm, Children SAF population
Table 3.20	Relatedness of serious adverse events (SAEs), by study cohort and study arm, Safety population

Table 3.21	Outcome of serious adverse events (SAEs), by study cohort, and study arm in Safety population
Table 3.22	Summary of deaths by study cohort and study arm, Safety population

7. Statistical Listings to be Generated

Subject Data Listings

Listing 1.1 Subjects who are screen-failures

Listing 1.2 Subjects who discontinued study after randomizations

Listing 1.3 Listing of protocol deviations

Listing 1.5 Exclusion of Data from PP populations

Safety Listings

Listing 2.1 Listing of Serious adverse events (SAEs)

Listing 2.2 Listing of Suspected unexpected serious adverse reactions (SUSARs)

Listing 2.3 Listing of Adverse events (AEs)

Listing 2.4 Listing of Immediate adverse events (IAEs)

8. References

Gamble, C., Krishan, A., Stocken, D., Lewis, S., Juszczak, E., Doré, C., ... & Loder, E. (2017). Guidelines for the content of statistical analysis plans in clinical trials. *Jama*, ***318*****(23)**, 2337-2343.

NIFTY Study Data Management Plan version 1.2 dated 25th Feb 2020

Approved NIFTY protocols version 1.4 dated 26th Nov 2020
